# MPP6 stimulates both RRP6 and DIS3 to degrade a specified subset of MTR4-sensitive substrates in the human nucleus

**DOI:** 10.1093/nar/gkac559

**Published:** 2022-07-29

**Authors:** Naoko Fujiwara, Maki Shigemoto, Mizuki Hirayama, Ken-ichi Fujita, Shigeto Seno, Hideo Matsuda, Masami Nagahama, Seiji Masuda

**Affiliations:** Graduate School of Biostudies, Kyoto University, Kyoto, Kyoto 606-8502, Japan; Graduate School of Biostudies, Kyoto University, Kyoto, Kyoto 606-8502, Japan; Graduate School of Biostudies, Kyoto University, Kyoto, Kyoto 606-8502, Japan; Graduate School of Biostudies, Kyoto University, Kyoto, Kyoto 606-8502, Japan; Division of Gene Expression Mechanism, Institute for Comprehensive Medical Science, Fujita Health University, Toyoake, Aichi 470-1192, Japan; Graduate School of Information Science and Technology, Osaka University, Suita, Osaka 565-0871, Japan; Graduate School of Information Science and Technology, Osaka University, Suita, Osaka 565-0871, Japan; Laboratory of Molecular and Cellular Biochemistry, Meiji Pharmaceutical University, Kiyose, Tokyo 204-8588, Japan; Graduate School of Biostudies, Kyoto University, Kyoto, Kyoto 606-8502, Japan; Department of Food Science and Nutrition, Faculty of Agriculture Kindai University, Nara, Nara 631-8505, Japan; Agricultural Technology and Innovation Research Institute, Kindai University, Nara, Nara 631-8505, Japan; Antiaging center, Kindai University, Higashiosaka, Osaka 577-8502, Japan

## Abstract

Recent *in vitro* reconstitution analyses have proven that the physical interaction between the exosome core and MTR4 helicase, which promotes the exosome activity, is maintained by either MPP6 or RRP6. However, knowledge regarding the function of MPP6 with respect to *in vivo* exosome activity remains scarce. Here, we demonstrate a facilitative function of MPP6 that composes a specific part of MTR4-dependent substrate decay by the human exosome. Using RNA polymerase II-transcribed poly(A)^+^ substrate accumulation as an indicator of a perturbed exosome, we found functional redundancy between RRP6 and MPP6 in the decay of these poly(A)^+^ transcripts. MTR4 binding to the exosome core via MPP6 was essential for MPP6 to exert its redundancy with RRP6. However, at least for the decay of our identified exosome substrates, MTR4 recruitment by MPP6 was not functionally equivalent to recruitment by RRP6. Genome-wide classification of substrates based on their sensitivity to each exosome component revealed that MPP6 deals with a specific range of substrates and highlights the importance of MTR4 for their decay. Considering recent findings of competitive binding to the exosome between auxiliary complexes, our results suggest that the MPP6-incorporated MTR4-exosome complex is one of the multiple alternative complexes rather than the prevailing one.

## Graphical Abstract

**Graphical Abstract:**
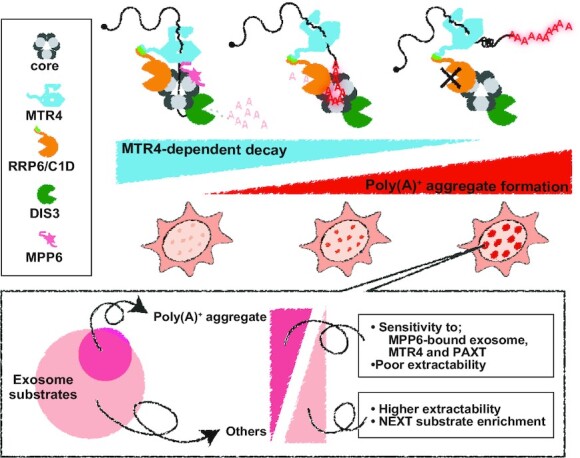
MMP6-bound nuclear exosome decays a specific subset of poly(A)^+^ substrates. MPP6 ensures the proper positioning of a specific cofactor within the exosome complex, hence the optimal decay of the substrates.

## INTRODUCTION

Transcription from eukaryotic genomes is pervasive and generates not only classical protein-coding mRNAs and stable, functional noncoding RNAs, but also short-lived transcripts. These have been shown to functionally regulate a variety of cellular processes, including antisense RNA transcriptional promotion, chromatin regulation, DNA break repair, controlling cell-fate decisions and pluripotency (1–4). Moreover, degradation activities that promote proper RNA processing and rapidly target short-lived RNAs are essential for the accurate expression of genomic information and involve multiple types of nuclease (3–7). Of these, the exosome is an essential and highly conserved multimeric 3’-5’ exonuclease complex responsible for various nucleolytic processes (4,6,8). The exosome's enzymatically inactive ‘core’ is a barrel-shaped structure consisting of nine components, six of which—referred to as RNase PH-like proteins—form a hexameric ring. The remaining three S1/KH domain-containing proteins bind to the top of this ring to form a central channel structure (9–12).

The catalytic nuclease itself binds to the PH-like domain at the bottom of the barrel via a cysteine-rich motif in its N-terminal domain (13–20). In yeast (*Saccharomyces cerevisiae*), Rrp44p binds to the core both in the nucleus and in the cytoplasm, while in humans, DIS3 binds in the nucleus and DIS3L1 in the cytoplasm (19,20). DIS3 and DIS3L1 are homologues of Rrp44p, an enzyme capable of both processive exonuclease activity derived from its C-terminal RNase II/R (RNB) domain and endonuclease activity exhibited by its N-terminal PilT N-terminal (PIN) domain (12,14,19–21). These two activities function cooperatively *in vivo*, and in yeast this can lead to synthetic growth defects caused by mutations that inactivate both activities (14,15). In the cytoplasm of human cells, another Rrp44p homologue, DIS3L2, also exists, but it lacks the PIN domain and does not bind to the exosome (22–24). In addition to these Rrp44p homologues, another nuclease—known as Rrp6p in yeast and RRP6 in humans—is a distributive exonuclease belonging to the RNase D family, and binds to the core in the nucleus in both species (25–27). RRP6 interacts with the core via its C-terminal region, while its N-terminus is coordinated on the S1/KH cap of the core, opposite to the position of DIS3 within the complex (28–31).

In *S. cerevisiae*, a relatively broad range of substrates has been observed for both Rrp44p and Rrp6p, as well as synthetic lethality for *rrp6Δ* and *rrp44exo-*, suggesting that these molecules act on common substrates (10,32). In humans, DIS3 is excluded from the nucleolus and is distributed in the nucleoplasm, while RRP6 is enriched in the nucleolus (19,33). Furthermore, DIS3 is responsible for most of the nucleoplasmic substrate degradation by the exosome, whereas the involvement of RRP6 has been proven only for a limited number of substrates such as for rRNA and snoRNA processing (34–37).

The broad substrate specificity of the exosome is achieved not only by compartment-specific nucleases but also by various cofactors (4,6,8). In various nucleolytic processes in the cytoplasm, including RNA turnover, surveillance, and interference, the Ski2–Ski3–Ski8 (Ski) complex is known to function with the core (38). In *S. cerevisiae*, the Trf4/5-Air1/2-Mtr4 polyadenylation (TRAMP) complex is involved in a wide range of nuclear substrate degradation (39–45), while in humans, reflecting a greater diversity of substrates, two additional complexes known as NEXT (nuclear exosome targeting) and PAXT [poly(A) tail exosome targeting] have also been identified as nuclear cofactors (46,47). The human TRAMP complex has been shown to be enriched in the nucleolus and is involved in the clearance of 5' ETS during rRNA processing and the decay of telomerase RNAs (hTRs) (48–52). The continuous addition of adenines to the 3' end of either the 5' ETS or hTRs by PAPD5 in TRAMP is essential for these processes. PAPD5 (also known as TRF4-2 and TENT4B) is described as a ‘non-canonical’ poly(A) polymerase (PAP), in contrast to canonical PAPs such as PAPOLA and PAPOLG (PAP A and PAP G), which function in adenylation coupled transcription termination mediated by polyadenylation signals (PAS) embedded in transcripts (53–57). Relative to canonical PAPs, the tailing of transcripts by PAPD5 is more prone to incorporate guanosines as well as adenines (58).

In the human nucleoplasm, the degradation of molecules with an unprotected 3' end, such as promoter upstream transcripts/upstream antisense RNAs (PROMPTs/uaRNAs), bidirectional enhancer RNAs (eRNAs), hTRs, spliced introns and intron-encoded snoRNAs, is carried out soon after transcription by NEXT, which contains a zinc-finger protein, ZCCHC8, as well as RNA-binding motif protein 7 (RBM7), which has an affinity for polypyrimidines (46,59–63). On the other hand, nucleoplasmic degradation of molecules whose 3' ends have been processed and polyadenylated by canonical PAPs is carried out by the PAXT complex, which contains ZFC3H1, a zinc-finger protein, and PABPN1, which has an affinity for polyadenines (47,64–68). An analogous poly(A)^+^ tail-mediated decay system has also been found in *Schizosaccharomyces pombe* (69–72). The polyadenylated forms of PROMPTs, eRNAs and variant snRNAs, products of premature cleavage and polyadenylation/intronic polyadenylation (PCPA/IPA), subsets of pre-mRNAs, and relatively long noncoding RNAs such as snoRNA host gene (*SNHG*)-derived molecules, have been identified as substrates for this pathway in humans (65–68,73–81). In addition, it has been observed that these substrates have longer poly(A)^+^ tails than exosome-insensitive poly(A)^+^ transcripts when stabilized under exosome-inhibited conditions (66). It is also known that PAXT tethers stabilized substrate molecules within distinct domains of the nucleus to achieve efficient transcript silencing (82,83). In these domains, ZC3H3 and RBM26/RBM27 have been recently identified as novel PAXT-composing candidates (84). Although such distinctive characteristics can be captured for substrates specific to NEXT or PAXT, the sensitivity of a substrate to cofactors is not always mutually exclusive. As seen in PROMPT degradation, when a substrate bypasses the NEXT pathway, the downstream PAS is sometimes utilized and molecules that acquire a poly(A)^+^ tail are subsequently degraded by PAXT (85). Perhaps reflecting the complexity in substrate sensitivity to cofactors, many substrates identified as targets of the nuclear exosome show ambiguous sensitivity to cofactors, despite the presence of a poly(A)^+^ tail at their 3' ends (86,87).

MTR4, an RNA helicase, is a common component found in many nuclear cofactors, including NEXT, PAXT and TRAMP (46,47,49,88,89). MTR4 competes with RNA maturation/export factors for binding to the cap-binding complex (CBC) at the 5' end of RNAs and thereby determines whether transcripts are matured or decayed (79,86,90–93). NRDE2, a protein enriched in nuclear speckles, inhibits MTR4 recruitment onto transcripts and negatively regulates exosome function by binding to MTR4, which inhibits its interactions with the CBC and the exosome (94). Competition for binding to MTR4 is also observed among RNA decay cofactors (95,96). Hence, depending on the complex it forms, MTR4 plays a pivotal role in determining the fate of RNA.

Recent detailed structural analyses have revealed how MTR4 is recruited to the top of the core barrel—i.e. the S1/KH domain side of the exosome. The N-terminal PMC2NT domain of RRP6, located at the upper S1/KH cap, binds to cofactor C1D and provides a binding surface for the N-terminus of MTR4 (97,98). Furthermore, MPP6 (also known as MHOPSPH6), a cofactor whose internal region interacts with the S1/KH domain, binds to the RecA domains of MTR4 at its N-terminus (99–102). The interaction surface of MTR4 with MPP6 overlaps with ZCCHC8 in the NEXT complex, and competition is anticipated between the formation of MPP6-incorporating and NEXT-binding exosomes (103). Detailed *in vitro* reconstitution experiments have suggested that both MPP6-bound and RRP6-bound MTR4 support substrate decay by DIS3, which is located on the opposite side of the MTR4-binding surface, at the bottom PH domain of the core, as well as substrate decay by RRP6 (99–102).

Despite vigorous *in vitro* analyses of the functional significance of MPP6 in supporting MTR4-core binding, *in vivo* evidence for this is quite limited. MPP6 was first identified in humans as an exosome cofactor that is essential for rRNA processing and has subsequently been reported to function in degrading a wide range of nuclear substrates in yeast (33,104). In *S. cerevisiae*, *mpp6Δ* exhibits synthetic lethality with a deletion of either *RRP6* or *RRP47*, a C1D homologue whose protein expression is interdependent with Rrp6p; this reflects the functional redundancy between Mpp6p and Rrp6p (33,97,104–106). It has been also reported that yeast Mpp6p promotes degradation by Rrp44p, but not by Rrp6p, of transcripts whose transcription has been terminated by the Nrd1p-Nab3p-Sen1p complex (44,45,107). This indicates that Mpp6p differs in its extent of contribution to Rrp44p and Rrp6p functions. The N-terminal deletion of Mpp6p, which prevents it from binding to Mtr4p, in combination with *rrp6Δ*, resulted in synthetic lethality of yeast cells, thereby suggesting that Mpp6p has physiological importance involved in maintaining interactions between Mtr4p and the core (100). However, to date, we do not yet understand the range of substrates handled by MPP6, the determinants of its substrate specificity, or the functional differences between the MPP6-mediated MTR4-core complex and complexes sustained by RRP6, especially in humans. Furthermore, it is also unclear to what extent the synthetic lethality of *rrp6Δ* and *mpp6Δ* observed in yeast reflects their Mtr4p-core supporting abilities, since Rrp6p is a nuclease as well as a bridge molecule between Mtr4p and the core.

In this paper, we demonstrated that MPP6, through its binding to MTR4, facilitates the degradation of a certain class of poly(A)^+^ substrates by RRP6 and DIS3 in the human nucleus. Rather than functioning on whole exosome substrates, MPP6 acts on a limited subset of substrates with specific features, including transcripts with lower extractability emerging from short transcription units and higher sensitivity to the PAXT cofactor rather than the NEXT. In addition, MPP6 seems essential for the decay of these substrates by DIS3 even in situations where a substantial interaction between MTR4 and the exosome core is maintained by RRP6. Our findings provide important insight into the mechanisms by which MPP6 influences the substrate specificity of the *in vivo* exosome complex by regulating the mechanism of MTR4 recruitment and the type of cofactors it binds.

## MATERIALS AND METHODS

### Reagents and antibodies

Primers, siRNAs, oligoprobes, expression plasmids and antibodies used in this study are described in Supplementary Table S1. Plasmid construction was basically performed as follows: Fragments containing genes of interest were obtained by PCR amplification using the human cDNA library as a template. Amplified fragments digested with adequate restriction enzymes were ligated with expression vectors indicated in Supplementary Table S1. pcDNA5/FRT/TO/FLAG vector was generated by ligation of the fragment of 3xFLAG Coding Sequence (CDS), which was amplified from p3xFLAG-CMV-10 (MilliporeSigma, Burlington, MA) by PCR and digested by HindIII, with pcDNA5/FRT/TO (Thermo Fisher Scientific, Waltham, MA) using a HindIII site. To construct pcDNA5/FRT/TO/RRP41-FLAG expressing vectors, 3xFLAG CDS cut out from pcDNA5/FRT/TO/FLAG using HindIII restriction sites was first ligated with the HindIII digested pCMV-Tag4 a (Agilent Technologies, Santa Clara, CA), and using this vector as a template, PCR to add a stop codon at 3’ end of 3xFLAG CDS was performed. Resulting 3xFLAG CDS fragment (digested by EcoRI and XhoI) and PCR amplified RRP41 CDS fragment (digested by KpnI and EcoRI) were ligated into KpnI and XhoI digested pcDNA5/FRT/TO. For generating pcDNA5/FRT/TO/EGFP-DIS3 expressing vectors, DIS3 CDS was first inserted into BglII and SalI digested pEGFP-C1 using BamHI and XhoI restriction sites then PCR amplified EGFP-DIS3 CDS was inserted into HindIII and XhoI digested pcDNA5/FRT/TO using In-Fusion HD Cloning Kit (Takara Bio Inc., Shiga, Japan). All the constructs obtained were verified by sequencing.

Animal experiments to obtain antisera were performed according to the guidelines of Animal Committee at Kyoto University (permission number: Lif-K14004).

### Cell culture and cell lines

U2OS, HeLa, A549, MCF7 and their derivative cell lines expressing recombinant proteins were maintained in Dulbecco's modified Eagle's medium (DMEM, FUJIFILM Wako Pure Chemical Corporation, Osaka, Japan) supplemented with 10% heat-inactivated fetal bovine serum (FBS) at 37°C in a humidified chamber (5% CO_2_). Plasmid and siRNA transfections were performed using Lipofectamine 2000 according to the manufacturer's protocol (Thermo Fisher Scientific). Experiments were basically performed at 72 h post-transfection, and modifications are noted in the figure captions.

Flp-In T-REx cell lines stably expressing each gene of interest were established by transfecting both pcDNA5/FRT/TO vector containing each CDS of interest and pOG44 (Thermo Fisher Scientific). Transfected cells were selected in DMEM containing 100 μg/ml Hygromycin B (FUJIFILM Wako Pure Chemical Corporation) to obtain resistant clones. To obtain the EGFP expressing HeLa Flp-In T-REx cell line, pEGFP-C1 transfected cells were selected in media containing 10 μg/ml Puromycin (InvivoGen, San Diego, CA).

Doxycycline (dox, MilliporeSigma) was supplemented to DMEM at a concentration of 1000 ng/ml to induce the expression of each gene of interest in Flp-In T-REx cells. In rescue experiments combined with siRNA transfection, dox was added at 6 h after transfection, except for the rescue experiments with expressed FLAG-RRP6, dox was supplemented 48 h before siRNA transfection, removed during transfection and supplemented again 6 h after transfection.

Leptomycin B (Cayman Chemical, Ann Arbor, MI) was supplemented at a concentration of 10 ng/ml into the medium and cells were cultured in it for 10 h prior to fixation.

### Immunofluorescence staining and poly(A)^+^ RNA fluorescence *in situ* hybridization

Cells cultured on glass coverslips were fixed with 10% formaldehyde in PBS at room temperature for 20 min and permeabilized in PBS containing 0.1% Triton X-100 at room temperature for 10 min. Cells were then washed three times with PBS for 10 min each and blocked with 6% bovine serum albumin (BSA) in PBS at room temperature for 1 h. Samples were incubated with primary antibodies in PBS containing 1% BSA overnight at 4°C in a humidified chamber, washed three times with PBS for 10 min each, and subsequently incubated with secondary antibodies conjugated with either Alexa-488, Alexa-594 or Alexa-405 diluted in PBS containing 1% BSA. Stained samples were briefly fixed with 4% formaldehyde dissolved in PBS for 10 min at room temperature, washed with PBS, and subjected to the following procedure.

Poly(A)^+^ RNA fluorescence *in situ* hybridization [poly(A)^+^ FISH] was performed as previously described (108). For beta-globin mRNA detection simultaneously with poly(A)^+^ RNAs, *in situ* hybridization using a specific DNA oligoprobe labeled with Alexa-594 was performed as previously (109), and followed by the poly(A)^+^ FISH procedure. Sequences of oligoprobes used for *in situ* hybridization are provided in Supplementary Table S1. Chromosomal DNA was stained with 4',6-diamidino-2-phenylindole (DAPI, Thermo Fisher Scientific).

Fluorescence images were obtained with FV10i (Olympus, Tokyo, Japan), a laser scanning confocal microscopy, using the ×60 objective lens. Segmentation of the nuclei and the cytoplasm and subsequent quantification of poly(A)^+^ RNA signals was performed using the CellProfiler software v3.1.5 (110). Classification of the cells based on the fluorescent signals from FLAG-staining was carried out by the CellProfiler Analyst v2.2.1 (111) using a Random Forest Classifier. Line Plot analysis was performed using FV10-ASW v4.1 software (Olympus).

### Total RNA isolation, reverse transcription and real-time PCR

Total RNAs were extracted with Sepasol-RNA I Super G (Nacalai Tesque, Kyoto, Japan) according to the manufacturer's protocol with a modification in the extraction step. The modification is as follows. Cells (∼2 × 10^6^) were trypsinized, collected by centrifugation and washed with PBS. 250 μl of Sepasol-RNA I Super G was added and the extraction cycle of the 30-s incubation at 55°C followed by the 30-s vortexing was repeated 10 times. Extracted RNAs were treated with RNase-free RQ DNase I (Promega, Madison, WI) and extracted by citrate-buffered phenol/chloroform/isoamyl alcohol and ethanol precipitated. 250 ng of DNase I treated total RNAs mixed with 25 pmol dT_25_ primer were incubated at 65°C for 5 min and then cooled on ice. To this mixture, reverse transcription was performed by the steps of 23°C for 10 min, 55°C for 60 min and 75°C for 15 min, using Superscript III (Thermo Fisher Scientific). Random9 primed cDNA synthesis was performed using ReverTraAce (TOYOBO, Osaka, Japan) according to the manufacturer's instructions. Real-time PCR was performed with TB Green Premix Ex Taq II (Tli RNaseH Plus) (Takara Bio Inc.) and analyzed by Thermal Cycler Dice real time system II (Takara Bio Inc.). Primer sets and real-time PCR conditions are described in Supplementary Table S1.

### Immunoblotting of cytoplasmic and nuclear extracts

Cell extracts were prepared based on the method described previously (112). Cells were washed with PBS, digested with trypsin and collected by centrifugation. Packed cell volume (PCV) was estimated. Cell pellets were suspended carefully in ×5 PCV volume of solution A (10 mM HEPES–KOH at pH, 7.9, 1.5 mM MgCl_2_, 10 mM KCl, 0.2 mM PMSF and 0.5 mM DTT) and immediately centrifuged at 100 × *g* for 10 min. After removing the supernatant, cell pellets were suspended again carefully in ×3 PCV volume of solution A and incubated on ice for 10 min. Suspended cells were homogenized by 10-s vortexing, and centrifuged at 1500 × *g* for 10 min. The supernatant was removed and stored as the cytoplasmic extract. Nuclei pellets were resuspended in ×1/2 PCV volume of Low Salt buffer (20 mM HEPES–KOH at pH 7.9, 1.5 mM MgCl_2_, 20 mM KCl, 0.2 mM EDTA, 25% glycerol, 0.2 mM PMSF and 0.5 mM DTT) and ×1/2 PCV volume of High Salt buffer (20 mM HEPES–KOH at pH 7.9, 1.5 mM MgCl_2_, 1.4 M KCl, 0.2 mM EDTA, 25% glycerol, 0.2 mM PMSF and 0.5 mM DTT) was then added. Samples were rotated at 4°C for 30 min and centrifuged to obtain the supernatant nuclear extract. The total protein concentration in the cytoplasmic and the nuclear extract was determined by the Bradford assay (Nacalai Tesque).

Protein samples were mixed with 4× SDS sample buffer (190 mM Tris–HCl at pH 6.8, 40% glycerol, 0.8% SDS, 0.2% bromophenol blue, 40 mM DTT) and boiled for 2 min. Denatured samples were separated by SDS-PAGE and electro-transferred onto PVDF membrane using Trans-Blot Turbo System (Bio-Rad, Hercules, CA). The blotted membranes were blocked with 5% skim milk dissolved in PBS containing 0.1% Tween-20 (PBS-T) at room temperature for 1 h, rinsed with PBS-T and incubated with the primary antibodies at 4°C overnight, with gentle rotation. The membranes were washed three times with PBS-T for 10 min each and then incubated with HRP conjugated secondary antibodies at room temperature for 90 min, rotating, followed by three washes with PBS-T for 10 min each. PVDF membranes were developed with chemiluminescence reagent (MilliporeSigma) and detected with LAS 4000 mini (GE Healthcare Bioscience, Piscataway, NJ). ACTIN and GAPDH were used as loading controls. Quantification was performed using Image J v1.53a (National Institutes of Health).

### Immunoprecipitation

Immunoprecipitations were performed as previously described (113). Briefly, nuclear extract of 150–200 μg was mixed with a 2-fold volume of Nuclear Extract dilution buffer (20 mM HEPES–KOH at pH 7.9, 0.2 mM EDTA, 20% glycerol, 0.1% Triton X-100, 0.2 mM PMSF, 0.5 mM DTT). Diluted extracts were mixed with 5 μl antibody-conjugated beads, rotated continuously at 4°C overnight. The beads were washed four times with PBS containing 0.1% Triton X-100, 0.2 mM PMSF and 0.5 mM DTT, eluted with 4× SDS sample buffer and boiled for 2 min. Immunoblotting was performed as described above.

### Pulldown assay

Recombinant glutathione S-transferase (GST) fusion proteins were produced in *Escherichia coli* and purified with glutathione beads as described previously (113). Briefly, nuclear extracts diluted in PBS containing 0.1% Triton X-100, 0.2 mM PMSF and 0.5 mM DTT were preincubated with GST beads to remove proteins that interact with GST, and then reacted with GST fusion protein-bound beads for 3 h at 4°C, with gentle rotation. The beads were washed four times with PBS containing 0.1% Triton X-100, 0.2 mM PMSF, 0.5 mM DTT, eluted with 4× SDS sample buffer and boiled for 2 min. Immunoblotting was performed as described above.

### Prediction of secondary structures and alignment of MPP6

Secondary structure prediction of MPP6 was performed using a web tool, PSIPRED v4.0 (114), using human MPP6 sequence (NP_005783.2) as an input. Alignment of MPP6 homolog sequences among multiple species was performed with Multalin v5.4.1 (115), using MPP6 sequences from *Homo sapiens* (NP_005783.2), *Xenopus tropicalis* (NP_001016703.1), *Drosophila melanogaster* (NP_001286116.1), *Saccharomyces cerevisiae* (NP_014421.3) and *Schizosaccharomyces pombe* (NP_593967.1) as inputs.

### Preparation of nuclear RNA samples for Next-Generation Sequencing

To prepare Next-Generation Sequencing (NGS) samples, HeLa cells transfected with the siRNA of interest were trypsinized and split into a 10 cm dish as well as on a coverslip in a 12-well dish at 6 h post-transfection. At 72 h post-transfection, cells on the coverslip were fixed and subjected to *in situ* hybridization as described above. Simultaneously, cells in the 10 cm dish were collected by trypsinization, washed with PBS and divided into two samples. One of the samples was subjected to the nuclear extract preparation as described above to check the knockdown efficiency. The other sample was incubated with 250 μl ice-cold Nonidet P-40 (NP-40) containing buffer [50 mM Tris–Cl at pH 8.0, 100 mM NaCl, 5 mM MgCl_2_, 0.5% NP-40, 1 mM DTT, 20 units/ml Recombinant RNase Inhibitor (Takara Bio Inc.)] on ice for 5 min and centrifuged at 4°C, at 300 × *g* for 5 min. The supernatant was carefully removed and preserved as the cytoplasmic fraction protein sample. Nuclei pellets were resuspended in 250 μl ice-cold NP-40 containing buffer and 50 μl of the suspension was transferred to another tube and preserved as the nuclear fraction protein sample. Cytoplasmic and nuclear protein samples from equal numbers of cells were denatured by 4× SDS sample buffer and subjected to immunoblot analysis. Nuclei were collected by centrifugation at 4°C, at 300 × *g* for 5 min and suspended in 250 μl Sepasol-RNA I Super G. RNAs were extracted according to the manufacturer's protocol with a modification in the extraction step. The extraction step of the sonication-employing method (sonic-method) was carried out by repeating 20 cycles of sonication at 50% output for 1 s and standing still for 1 s using a Q125 sonicator (Qsonica, Newtown, CT), followed by 10 times repeated cycles of the incubation at 55°C for 30 s and vortexing for 30 s. The extraction step of the hot-phenol method (hp-method) without sonication was performed as described above. Control KD and RRP6/DIS3 KD samples were prepared in duplicate amounts to provide samples from both the hp-method and the sonic-method. Extracted RNAs were DNase I treated and extracted again by citrate-buffered phenol/chloroform/isoamyl alcohol and ethanol precipitated.

### NGS library preparation and sequencing

Samples were prepared in two biological replicates. Nuclear total RNAs prepared as indicated above were analyzed by Agilent 2100 Bioanalyzer (Agilent Technologies) using Agilent RNA 6000 Nano kit (Agilent Technologies). To 10 μl of 50 ng/μl total nuclear RNA, 1 μl of 1:100 diluted ERCC Spike-in Control (Thermo Fisher Scientific) was added. cDNA libraries with barcoding were generated using QuantSeq 3'mRNA-Seq Library Prep Kit (FWD) (LEXOGEN, Vienna, Austria) according to the manufacturer's instructions. Generated libraries were analyzed by Agilent 2100 Bioanalyzer using High sensitivity DNA kit (Agilent Technologies) and also quantified by qPCRs. We mixed 3 nM of each library, and this pooled library (containing 18 samples in total) was 150 nucleotide pair-end sequenced on a Hiseq2500 (Illumina inc., San Diego, CA) at GENEWIZ (South Plainfield, NJ) to yield 149.605 Gbases (498,683,027 reads) in total. Obtained sequences were demultiplexed based on the index information. Sequence data have been deposited at GEO: GSE184274.

### Sequence quality control and mapping

Sequences were trimmed using TrimGalore v0.6.5 (https://doi.org/10.5281/zenodo.5127899) to remove low-quality base calls from the 3’end of the reads, to detect and remove i7 index (AGATCGGAAGAGC) and to discard trimmed reads shorter than 20 bases. Remaining R1 reads were mapped to the ERCC sequence combined GRCh37.75 as single-end reads, using STAR aligner v2.5.3a (116) with options of –outFilterType BySJout –outFilterMultimapNmax 1, to generate bam files. Bam files were indexed using samtools v1.3.1 (117).

### Read count and differential expression analysis

Mapped reads were counted by HTseq htseq-count v0.9.5 (118) with options of -m intersection-nonempty -s yes -f bam –r pos (feature type to count: default, exon), and the genome annotation for GRCh37.75 from ENSEMBL combined with the ERCC spike-in annotation from Thermo Fisher Scientific. After the cut-off filter of at least 10 reads in total across all samples to each gene, read counts from each replicate were separately subjected to the subsequent analyses using DESeq2 v1.24.0 (119). Regularized log transform (rlog) counts, normalized using ERCC counts as a control, were subjected to the principal component analysis (PCA). Raw counts were used as input counts for DESeq2 differential expression analysis. Genes with adjusted *P*-value <0.05 and log_2_ FC >0.85 were filtered as genes with a significant upregulation. Data visualization was performed with ggplot2 (120) in R software [R Development Core Team (2020) R: A Language and Environment for Statistical Computing. *R Found. Stat. Comput*.]. SparK v2.6.2 (121) visualized mapped reads within the genome region of interest using bedgraphs generated from bam files by deeptools v3.4.3 (122).

### Reanalysis of CLIP data

DIS3 PAR-CLIP data (37) and RRP6 iCLIP data (36) were obtained from GEO: GSE64332 and GSE120574, respectively. RRP6 bigwig files were converted into bedgraphs and liftovered to hg19. Obtained bedgraphs and genome annotation for GRCh37.75 from ENSEMBL were used as the inputs for ChIPseeker (123), an R package, to annotate CLIP peaks. Peak scores overlapping the annotated genes were summed by gene [regions annotated as 5’ UTR, 3’UTR, exon, intron and downstream (≤300 bp) were analyzed] to be compared between the exosome substrates and the non-substrates.

### *In vitro* DIG-labeled probe synthesis

Specific probes to detect*RNVU1-14*, *RNVU1-20* and *RPL27*A were synthesized by *in vitro* transcription using T7 RNA polymerase (Takara Bio Inc.) and DIG RNA labeling mix (MilliporeSigma). T7 promoter-attached templates of *RNVU1-14 and RPL27*A were amplified by PCR from cDNA of RRP45 depleted HeLa cells and sequenced. T7 attached *RNVU1-20* template was amplified from *RNVU1-20* gene cloned in pcDNA5/FRT/IRES-dsRed vector. DNA templates were purified using DNA Clean & Concentrator-25 columns (Zymo Research, Irvine, CA). Synthesized labeled probes were purified using NucleoSpin RNA Clean-up XS columns (Takara Bio Inc.).

### Specific RNA fluorescence *in situ* hybridization

Fixed and permeabilized cells were prepared as described for poly(A)^+^ RNA FISH samples in the above section. Specific RNA FISH experiments were conducted following the procedure previously described, except that we used the tyramide signal amplification to detect hybridized DIG-labeled probes (124). The detection of hybridized DIG-labeled probes was conducted as follows; samples were rinsed once with PBS, blocked with 6% BSA in PBS at room temperature for one hour and incubated with mouse monoclonal antibody against DIG in PBS containing 1% BSA at room temperature for 3 h. Then, samples were washed three times with PBS for 10 min each and subsequently incubated with secondary antibodies conjugated with HRP diluted in PBS containing 1% BSA. After washing twice with PBS, samples were washed once with 100 mM Tris–HCl buffer at pH 7.4 containing 150 mM NaCl and incubated with Alexa Fluor 594 Tyramide Reagent (Thermo Fisher Scientific) diluted in 100 mM Tris–HCl buffer at pH 7.4 containing 150 mM NaCl and 0.0015% hydrogen peroxide (Nacalai Tesque) at room temperature for 10 min. Samples were rinsed three times with PBS and once with 2× SSC, then subjected to the poly(A)^+^ RNA FISH procedure using the Alexa-488 dT_45_ probe as described above.

### Analysis on NEXT/PAXT-sensitivity annotated PROMPTs

Annotations of PROMPTs were obtained from previously published data (85). Data was converted using LiftOver to generate GTF in hg19. Our mapped reads were counted by HTseq htseq-count v0.12.4 (118) using this GTF. Pseudocount + 1 applied read counts were subjected to differential expression analysis using DESeq2 as described above. Analyses were focused only on PROMPTs significantly stabilized by RRP6/DIS3 KD in our NGS analysis.

### Nuclear RNA profile changes by mRNA export block

The nuclear RNAs from Control KD HeLa cells and TAP KD HeLa cells were prepared using the hp-method as described above. cDNA libraries were prepared using TruSeq Stranded mRNA Library Prep Kit (Illumina Inc.), following the manufacturer's instruction and 150 nucleotide pair-end sequenced on NovaSeq 6000 (Illumina inc.). Obtained sequences were demultiplexed based on the index information. Sequence data were deposited at Japanese Genotype–Phenotype Archive (JGA): JGAS000294. Reads were further trimmed, mapped, counted and subjected to DESeq2 differential expression analysis as described above, except that reads were treated as pairs.

## RESULTS

### RRP6 and DIS3, two nucleases specifically associated with the nuclear exosome, redundantly degrade a subset of Pol II-transcribed nucleoplasmic poly(A)^+^ substrates

**Figure 1.**
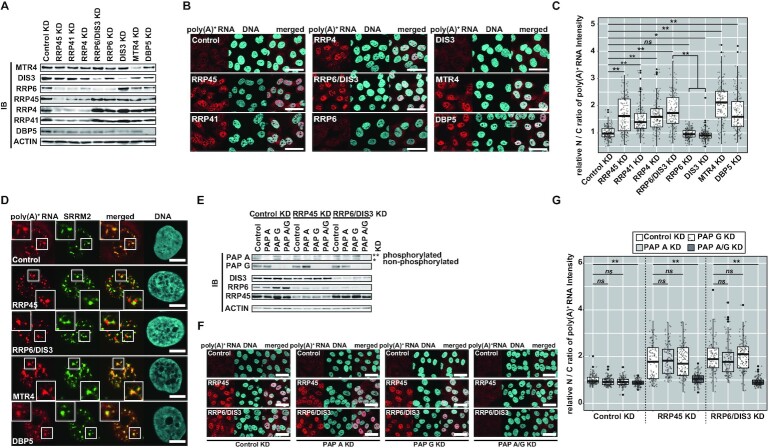
Characteristics of nucleoplasmic poly(A)^+^ aggregates resulting from nuclear exosome impairment. (**A**–**C**) A robust nuclear poly(A)^+^ RNA accumulation is elicited in U2OS cells by nuclear exosome inhibition. (**D**) Subnuclear localization of poly(A)^+^ aggregates in U2OS cells. (**E**–**G**) PAP A and PAP G redundantly adenylate substrates responsible for poly(A)^+^ foci emergence upon exosome inhibition. (A, E) Immunoblot analysis of the nuclear extracts to confirm the specific depletion of each exosome component. Conditions of transfected siRNAs and used cell lines are as indicated in the upper notes. In (E), the asterisk (*) indicates signals from non-phosphorylated PAP A, whereas a double asterisk (**) indicates signals from phosphorylated PAP A. (B, F) The subcellular distribution of poly(A)^+^ RNAs was visualized by *in situ* hybridization using Alexa594 labeled dT_45_ probe [poly(A)^+^ FISH]. Conditions of transfected siRNAs and used cell lines are indicated at the bottom of and in the panels. Scale bar = 50 μm. (C, G) Quantification of the nuclear/cytoplasmic (N/C) ratio of poly(A)^+^ FISH signal intensity from the experiment in (B), (F), respectively. Relative values normalized by the mean value of Control KD within each cell line are shown. Statistical analysis was performed using Steel–Dwass test. *P < 0.05, **P < 0.01, *ns*: not significant, *n* = 100. (D) Immunostaining followed by poly(A)^+^ FISH visualized SRRM2 simultaneously with poly(A)^+^ RNAs in U2OS cells. Factors depleted are indicated in panels. Magnified pictures are shown in insets. Scale bar = 10 μm.

As previously found, a remarkable nuclear poly(A)^+^ RNA accumulation is induced when the exosome is inhibited in human cells (83,87,108). To elucidate how RRP6 and DIS3, two nucleases that specifically associate with the nuclear exosome, function in the decay of these poly(A)^+^ substrates, using the U2OS cell line as a model, we depleted several components of the exosome core, as well as RRP6 and DIS3, and analyzed the subcellular localization of poly(A)^+^ RNAs under each condition (Figure 1A-C).

In contrast to control cells (Control KD cells), where poly(A)^+^ RNA distribution is predominantly cytoplasmic and weakly found in the nuclear speckles, poly(A)^+^ RNAs strikingly accumulated as punctate foci in the nucleus upon depletion of either component of the exosome core, RRP45, RRP41 or RRP4 (Figure 1B–D). When either RRP6 or DIS3 was solely knocked down, we observed no significant nuclear accumulation of poly(A)^+^ RNAs, while the simultaneous knockdown of RRP6 and DIS3 (RRP6/DIS3 KD) resulted in the emergence of robust nuclear poly(A)^+^ RNA foci. These remarkable poly(A)^+^ aggregates resided in discrete domains from the nuclear speckles [visualized by SRRM2 staining (125)], consistent with the characteristics of stabilized nuclear exosome substrates observed in the previous reports [Figure 1D, contrastly, in cells deprived of DBP5, an essential factor for mRNA export (126), poly(A)^+^ RNAs accumulated in the speckles (Figure 1A–D)] (83,87). These data suggest that RRP6 and DIS3 redundantly degrade these poly(A)^+^ substrates.

Next, we tried to identify the polymerase responsible for generating the poly(A)^+^ foci forming substrates. The depletion of PAPD5, a TRAMP-associated non-canonical PAP known to adenylate RNA polymerase I-transcribed rRNAs and facilitate their processing, did not restore nuclear poly(A)^+^ RNA accumulation in RRP45 KD and RRP6/DIS3 KD cells (Supplementary Figure S1A and B) (87). Conversely, depleting both PAP A and PAP G (two canonical PAPs) almost completely abolished nuclear poly(A)^+^ RNA accumulation caused by the exosome inhibition; this was not found under conditions where only PAP A or PAP G were depleted alone (Figure 1E–G). These data suggest that two canonical PAPs adenylate poly(A)^+^ substrates redundantly, implying that exosome substrates forming poly(A)^+^ aggregates are mainly transcribed by RNA polymerase II (Pol II), although rRNAs are one of the most abundant substrates of the nuclear exosome. This interpretation is also supported by the fact that the poly(A)^+^ foci that emerged due to exosome dysfunction were excluded from the nucleolus (Supplementary Figure S1C).

### MTR4 inactivation resulted in poly(A)^+^ substrate stabilization

The phenotypes derived from MTR4 KD in human cells are more enigmatic. Recently, two groups have reported that different phenotypes are derived from MTR4 KD in human cells, including one with robust nuclear poly(A)^+^ RNA accumulation, and another with diminished poly(A)^+^ RNAs in the nucleus, thought to be explained by leakage of stabilized poly(A)^+^ substrates into the cytoplasm under MTR4-depleted conditions (82,83,87). In the above experiment, we observed a remarkable nuclear poly(A)^+^ RNA accumulating phenotype resulting from MTR4-depletion in U2OS cells (Figure 1A–C).

**Figure 2.**
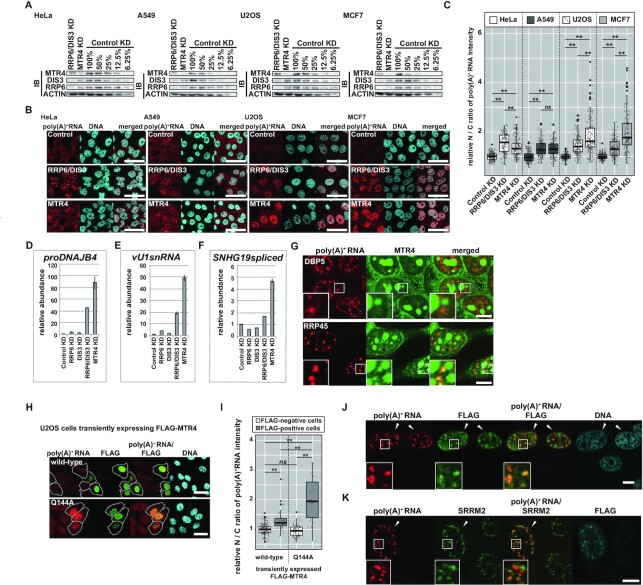
Substrates responsible for the nucleoplasmic poly(A)^+^ aggregates formed by exosome inhibition are also stabilized by MTR4 dysfunction. (**A**–**C**) The effects of MTR4 KD on the nuclear poly(A)^+^ RNA accumulation in HeLa, A549, U2OS and MCF cell lines. (**D**–**F**) MTR4 KD in HeLa cells causes a robust stabilization of exosome substrates in their adenylated forms. (**G**) Accumulation of MTR4 proteins to the exosome-inhibition induced poly(A)^+^ foci in U2OS cells. (**H**–**K**) Characterization of poly(A)^+^ aggregates in U2OS cells derived from the transient expression of Q144A, a helicase-null mutant of MTR4. (A) Immunoblot analyses were performed for the nuclear extracts to confirm specific depletion of the factors indicated at the top of the panels. For Control KD samples, nuclear extracts were also loaded in the indicated proportions. (B, H) Subcellular localization of poly(A)^+^ RNAs. Conditions of cell lines, expressed proteins and depleted factors are stated at the top of and in panels. In (H), transiently expressed FLAG-MTR4 proteins were visualized by FLAG-staining, and cells were categorized as either ‘FLAG-positive’ or ‘FLAG-negative’ cells using the Random Forest classifier in CellProfiler Analyst. White lines outline ‘FLAG-positive’ cells. Scale bar = 50 μm. (C, I) Quantification of (B), (H), respectively. Nuclear/cytoplasmic (N/C) ratios of poly(A)^+^ FISH signal were normalized by the mean value of Control KD within each cell line in (C), by the mean value of wild-type MTR4 expressing cells sorted as ‘FLAG-negative’ cells in (I). Statistical analysis was performed within each cell line using Steel–Dwass test following Kruskal–Wallis test. **P < 0.01, *ns*: not significant, *n* = 150 in (C), FLAG-negative wild-type MTR4; *n* = 157, FLAG-positive wild-type MTR4; *n* = 42, FLAG-negative Q144A MTR4; *n* = 82, FLAG-positive Q144A MTR4; *n* = 24 in (I). (D–F) RT-qPCRs were performed to quantify poly(A)^+^ tailed (D) *proDNAJB4*, (E) *vU1snRNA*, and (F) spliced *SHNG19*. Values are relative abundances of each transcript normalized to *GAPDH* and Control KD sample. Bars and error bars represent mean values ± *SD*. *n* = 3. (G) MTR4 was visualized by immunostaining simultaneously with poly(A)^+^ RNAs. Factors depleted are described in panels. Magnified pictures are shown in the insets. Scale bar = 10 μm. (J, K) Transiently expressed Q144A visualized simultaneously with poly(A)^+^ RNAs. In (K), SRRM2 was also immunostained. Arrowheads indicate FLAG-staining positive cells. Magnified pictures are shown in insets. Scale bar = 10 μm.

These conflicting data prompted us to examine the effect of MTR4-depletion on several cell lines, including HeLa, U2OS, A549 and MCF7 (Figure 2A–C). While RRP6/DIS3 KD resulted in obvious nuclear poly(A)^+^ RNA accumulation in all cell lines tested, the MTR4 KD-induced poly(A)^+^ RNA accumulating phenotype was more robust in U2OS and MCF7 cells than in HeLa and A549 cells (Figure 2B and C). Notably, accumulated poly(A)^+^ RNAs in MTR4-depleted U2OS cells tended to co-localize with nuclear speckles, in contrast to RRP6/DIS3 KD-derived poly(A)^+^ RNA foci (Figure 1D). A similar SRRM2-associated poly(A)^+^ RNA accumulation was also observed for exosome-deprived U2OS cells when they were simultaneously depleted in ZFC3H1 or PABPN1 (Supplementary Figure S2A-J). These data imply that, under PAXT-depleted conditions, a system to retain poly(A)^+^ substrates in the nucleus as punctate foci is disrupted in all cell lines tested, and also that a substantial portion of stabilized poly(A)^+^ substrates are still nuclear-retained in a speckle-associated manner in some cell lines, including U2OS and MCF7. In these cells, another mechanism, such as a specific retention system or less effective nuclear export, is anticipated, although further investigation is required to elucidate its structure and function.

Despite different phenotypes induced by MTR4 KD in each cell line, we found several lines of evidence that support MTR4’s involvement in degrading poly(A)^+^ substrates included in poly(A)^+^ RNA foci derived from exosome KD. Even in MTR4-depleted HeLa cells, which do not exhibit a prominent poly(A)^+^ RNA accumulating phenotype, substrates previously detected in the poly(A)^+^ RNA foci that resulted from impaired exosome activity—namely *proDNAJB4*, *vU1snRNA*, and spliced *SNHG19*—were stabilized as robustly as in RRP6/DIS3 KD cells (Figure 2D–F) (83). In addition, we found that MTR4 proteins accumulated at poly(A)^+^ foci in exosome-depleted cells but not in poly(A)^+^ sediments in DBP5 KD cells (Figure 2G). We also found that the overexpression of Q144A, an MTR4 mutant that lacks its helicase activity (88,89), provoked a dominant-negative phenotype to develop Q144A-containing poly(A)^+^ foci (Figure 2H–J) (87). The localization of these foci was distinct from nuclear speckles and analogous to the pattern observed for poly(A)^+^ foci in exosome KD cells (Figure 2K, Figure 1D, RRP45 KD and RRP6/DIS3 KD), suggesting that Q144A-caused poly(A)^+^ foci are derived from perturbed exosome function. Moreover, these transcripts accumulated via MTR4 dysfunction are redundantly adenylated by canonical PAPs (Supplementary Figure S2K–N). Taken together, we conclude that these manifested poly(A)^+^ substrates in exosome KD cells are degraded, at least partly, in an MTR4-dependent fashion, although the degree of nuclear poly(A)^+^ RNA accumulation induced by MTR4 KD differs between cell lines.

Given that these poly(A)^+^ substrates come through MTR4 helicase, how do these RNAs reach the DIS3 nuclease, which sits on the opposite side of MTR4 in the complex? It has been suggested that some substrates reach DIS3 through the channel structure, while others do so directly (127–133). To assess the contribution of the channel structure to poly(A)^+^ substrate digestion, we utilized a channel-occluding mutant (17,128,129) (Supplementary Figure S3A–I). We established HeLa Flp-In T-REx cell lines that stably expressed C-terminal FLAG-fused and RNAi-resistant RRP41s of either wild-type or R62E/A63D/R94E/R95E quadruple mutant (4M) form. Both expressed RRP41-FLAGs exhibited proper subcellular localization and complex formation (Supplementary Figure S3A, C and D) (17,29,128,129,134). Introducing the 4M-mutation to the exosome complex efficiently occluded the central channel, thereby inhibiting RNA access to DIS3 through the channel both *in vitro* and *in vivo*, while presumably maintaining the integrity of the surface within the S1/KH cap that has been shown to be utilized by substrate RNAs to reach RRP6 (17,29,128,129). In RRP6-depleted 4M-expressing cells, we observed a marked accumulation of nuclear poly(A)^+^ RNA, even though no obvious poly(A)^+^ substrate accumulation was found in RRP6 KD in either control or wild-type RRP41-expressing cells (Supplementary Figure S3A and B). These results suggest that RRP6 dominantly degrades these substrates in 4M-expressing cells, which means that most substrates depend on the channel to reach DIS3. Further support for this idea came from the fact that the nuclear poly(A)^+^ RNA accumulation derived from RRP41 KD was restored by 4M-mutant expression; this proved to be just as efficient as wild-type RRP41-expression. The likely mechanism was associated with maintaining the abundance of RRP6 since wild-type RRP41 but not 4M could restore the phenotype induced by RRP6/RRP41 KD (Supplementary Figure S3A, B and E–I).

### MPP6 facilitates both RRP6- and DIS3-mediated degradation of nucleoplasmic poly(A)^+^ substrates

**Figure 3.**
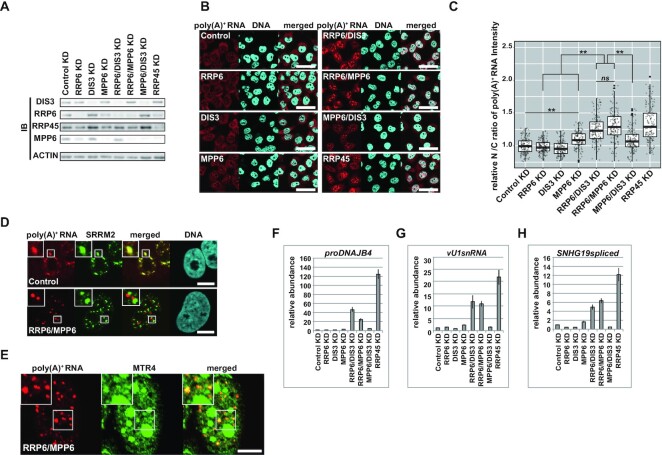
MPP6 facilitates both DIS3 and RRP6 to degrade nucleoplasmic poly(A)^+^ substrates. (**A**–**C**) Effect of MPP6 depletion on the decay of bulk poly(A)^+^ substrates in U2OS cells. (**D**, **E**) Characteristics of poly(A)^+^ aggregates formed in the U2OS nucleus by RRP6/MPP6 KD. (**F**–**H**) RT-qPCRs to evaluate the effect of MPP6 depletion on individual substrate stabilization. (A) Immunoblot analysis of U2OS nuclear extracts to confirm specific knockdown of factors described at top of the panels. (B) Poly(A)^+^ FISH under conditions of MPP6 depletion solely or simultaneously with RRP6 or DIS3. Knocked-down factors are stated in the panels. Scale bar = 50 μm. (C) Quantification of (B). Relative nuclear/cytoplasmic (N/C) ratio of poly(A)^+^ FISH signal normalized to the mean value of Control KD cells. Statistical analysis was performed using Steel–Dwass test following Kruskal–Wallis test. *P < 0.05, **P < 0.01, *ns*: not significant, *n* = 150. (D, E) Poly(A)^+^ RNAs were visualized simultaneously with SRRM2 in (D) and MTR4 in (E). Factors depleted are shown in the panels. Magnified pictures are shown in insets. Scale bar = 10 μm. (F–H) PCR was performed on dT_25_-primed cDNA synthesized using total RNA from whole cells to detect (F) *proDNAJB4*, (G) *vU1snRNA* and (H) spliced *SNHG19*. Values are shown as relative abundances of each transcript normalized to *GAPDH* and the value of the Control KD sample. Bars and error bars denote mean values ± SD. *n* = 3.

Using the poly(A)^+^ RNA accumulating phenotype as an indicator of the impaired nuclear exosome, we next explored the function of MPP6 in nuclear exosome activity. We depleted exosome-associating factors, including MPP6, either solely or simultaneously, and examined whether nuclear poly(A)^+^ RNA accumulation was induced under each condition in HeLa cells as well as U2OS cells (Figure 3A–C, Supplementary Figure S4A). As shown above, RRP6/DIS3 KD led to a strong increase in nuclear poly(A)^+^ RNA abundance (Figure 1B and C, Figure 3A–C, Supplementary Figure S4A). We also observed prominent nuclear poly(A)^+^ RNA accumulation induced by RRP6/MPP6 KD, similar to that observed under the RRP6/DIS3 KD condition. Diagnostic features of poly(A)^+^ foci resulting from exosome inhibition, such as discordance in the subnuclear localization with SRRM2, PAP A/G-dependent adenylation, and association with MTR4 proteins, were also observed for RRP6/MPP6 KD-derived poly(A)^+^ RNAs (Figure 3D and E, Supplementary Figure S4B), suggesting that MPP6 is essential for DIS3 to degrade these substrates in the absence of RRP6. The depletion of MPP6 alone resulted in only modest nuclear poly(A)^+^ RNA accumulation, and these levels were lower than those observed in either the RRP45- or RRP6/DIS3-depleted cells. Because RRP6 and DIS3, at least in the presence of MPP6, degrade these substrates redundantly and efficiently without assistance from the other, these data imply that MPP6 has a facilitative function for both RRP6 and DIS3. In contrast to strong poly(A)^+^ RNA accumulation in RRP6/MPP6 KD cells, MPP6/DIS3 KD cells showed only mild nuclear poly(A)^+^ RNA accumulation, indicating that RRP6 is not completely inhibited by the depletion of MPP6. The notion that RRP6 has residual activity in the absence of MPP6 is also supported by the mild accumulation of poly(A)^+^ substrates observed in 4M channel-occluded cells deprived of MPP6 (Supplementary Figure S4C and D). When the individual MTR4-sensitive poly(A)^+^ substrates evaluated in Figure 2D–F were used as reporters, we observed a similar MPP6 sensitivity as that of bulk poly(A)^+^ substrates (Figure 3F–H). RRP6/MPP6 KD cells showed an increased abundance of these transcripts, comparable to RRP6/DIS3 KD conditions, while depletion of either RRP6 or MPP6 resulted in a negligible or much lower effect. We also evaluated the effect of depleting C1D on poly(A)^+^ substrate decay. C1D is an obligatory factor for RRP6 expression and *vice versa* (33,105,106). As expected, C1D knockdown resulted in a diminished RRP6, while RRP6 knockdown almost abolished C1D (Supplementary Figure S4E). Reflecting on the interdependent expression of RRP6 and C1D, we also obtained similar results when C1D was knocked down instead of RRP6 (Supplementary Figure S4F). These data confirm some redundant functions between MPP6 and RRP6/C1D during the decay of poly(A)^+^ substrate RNAs. Nevertheless, the detailed mechanism, such as whether RRP6 functions as a nuclease and/or a binding surface for MTR4 cooperatively formed with C1D (97,98), remains unclear with the data thus far.

In the above experiments, we found that prolonged depletion of MPP6 led to a diminished amount of nuclear RRP6, but not to differences in DIS3 abundance (Figure 3A, Supplementary Figure S4G). This phenomenon was neither due to changes in the subcellular localization of RRP6, nor a decrease in the abundance of its transcript (Supplementary Figure S4H and I), which suggests that the regulation of RRP6 expression occurs at the post-transcriptional level. To minimize the effect of this phenomenon, we performed our experiments at 72 h after siRNA transfection—i.e. at a time when the co-depletion of RRP6 induced by MPP6 KD is not prominent. However, it is still possible that a subtle decrease in RRP6 elicited by MPP KD itself resulted in attenuated RRP6 activity on poly(A)^+^ substrate decay. Therefore, with the results reported thus far, we could not define the actual contribution of MPP6 in facilitating RRP6 as a nuclease (see the section below). However, it is notable that RRP6 KD did not result in a significant nuclear poly(A)^+^ RNA accumulating phenotype despite the fact that it also induces a decrease in MPP6 protein levels. This suggests that remarkable nuclear poly(A)^+^ RNA accumulation is caused only when a substantial population of MPP6 is depleted, even in RRP6 KD cells.

### MPP6 interacts with MTR4 in a region distinct from its core-binding site

**Figure 4.**
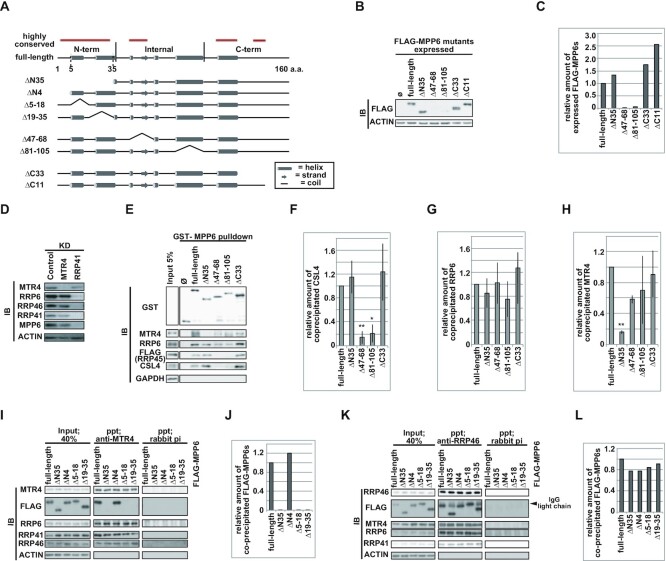
Domains of MPP6 required for interaction with MTR4 and the core. (**A**) Construction of MPP6 mutants. (**B**, **C**) Immunoblot analysis of nuclear extracts from HeLa Flp-In T-REx cell lines stably expressing FLAG-MPP6 mutants. (**D**) Co-depletion of MPP6 protein by knockdown of RRP41, an exosome core component. (**E**–**H**) Pulldown assay of GST-MPP6 mutants. (**I**–**L**) Immunoprecipitation (IP) experiments to identify the MPP6 domain responsible for the interaction with exosome components. (A) Structures predicted by PSIPRED are shown in the cartoon as stated in the inset. Highly conserved regions are indicated in red lines at the top of the structure cartoon. According to its predicted structure and its sequence conservation, MPP6 was divided into three domains, N-terminal (1–35 a.a.), internal (36–105 a.a.) and C-terminal (105–160 a.a.) domains. a.a. = amino acid. (B) Used cell lines are noted at the top. Ø represents FLAG only. (C) Quantification of chemiluminescent signals from (B). Represented values are relative amounts of each MPP6 mutant normalized to ACTIN signal and to FLAG-MPP6 full-length value. (D) Immunoblot analysis of nuclear extracts from HeLa cells deprived of indicated factors at the top. (E) Pulldown assay was performed on the nuclear extracts from FLAG-RRP45 expressing HeLa Flp-In T-REx cells. GST-MPP6 proteins were indicated at the top. (F–H) Quantification of chemiluminescent signals from three independent GST-pulldown assays including (E). Normalization was applied to (F) CSL4, (G) RRP6, and (H) MTR4 by the amount of each GST-MPP6 protein and by the value of GST-MPP6 full-length to obtain their relative coprecipitated amounts. Bars and error bars denote mean values ± SD. Statistical analysis was performed by Dunnett test. *P < 0.05, **P < 0.01. (I, K) IPs were performed to nuclear extracts from HeLa Flp-In T-REx cells expressing FLAG-fused MPP6 mutants using anti-MTR4 antiserum in (I) and anti-RRP46 antibody in (K), respectively. Rabbit pre-immune antiserum was used as a control in the precipitation step. (J, L) Quantification of chemiluminescent signals from (I) and (K), respectively. Relative precipitated amounts of the corresponding FLAG-MPP6 mutant were obtained as follows. The ratio of FLAG-MPP6 either to MTR4 in (I) or to RRP46 in (K) in each precipitate was normalized by the respective ratio in each input extract and by the value of the FLAG-MPP6 full-length sample.

To elucidate in greater detail the mechanism by which MPP6 stimulates the exosome to degrade nucleoplasmic poly(A)^+^ substrates, we constructed several MPP6 mutants and dissected their complex profiles. Because no three-dimensional structure of MPP6 was available when we started these analyses, we used a predicted secondary structure combined with alignments between human MPP6 and yeast MPP6 (Figure 4A) (114,115). Although the sequence of MPP6 is poorly conserved between species, the alignment of human and yeast MPP6 sequences demonstrated relatively high conservation within the N-terminal region (Figure 4A, Supplementary Figure S5A). This fact prompted us to create a mutant that contained a deletion of 35 amino acids in the N-terminal region. Based on the predicted secondary structure of this region, we also constructed ΔN4, Δ5–18 and Δ19–35 mutants, as well as several C-terminal truncated mutants and internal region-deleted mutants (Figure 4A).

We first tried to establish the cell lines that stably expressed each constructed MPP6 mutant. However, internal region-deleted mutants failed to express or expressed poorly in human cells (Figure 4B and C). Because core components are necessary for maintaining the expression of MPP6 (Figure 3A, Figure 4D), we speculated that the internal regions of MPP6 are critical for the interaction with the exosome core. To dissect the complex formation of these mutants, we performed a GST-pulldown assay. Each GST-fused MPP6 mutant was produced in *E. coli* and was purified to incubate with the nuclear extract prepared from FLAG-RRP45 expressing HeLa Flp-In T-REx cells. As a result, both N-terminal and C-terminal truncations did not alter MPP6 binding to either RRP6 or exosome core components, FLAG-RRP45 and CSL4 (Figure 4E–G). On the contrary, deletion of internal regions (47–68,81–105) significantly diminished the interaction between MPP6 and the core components, while a substantial proportion of RRP6 and MTR4 were still bound to each mutant protein (Figure 4E–H). These data suggest that the internal region of MPP6 is critical for binding to the core and that MPP6 associates with core-unbound RRP6 and MTR4 in a discrete domain that is distinct from its core-interacting region (33,99–102).

In the above experiment, we also found that the ΔN35 mutant failed to bind to MTR4 (Figure 4E and H). To verify this finding, we performed immunoprecipitation experiments using antisera against MTR4 or RRP46 with nuclear extracts prepared from cells expressing FLAG-fused full-length, ΔN35, ΔN4, ΔN5–18 or ΔN19–35 MPP6 mutant proteins, respectively (Figure 4I–L). In accordance with the experimental results of our pulldown assay (Figure 4E and H), the MTR4-MPP6 interaction disappeared when N-terminal 35 residues were deleted in MPP6 (Figure 4I and J). Both ΔN5–18 and ΔN19–35 also failed to interact with MTR4, whereas ΔN4 bound to MTR4 as efficiently as full-length MPP6 did, suggesting that the 5–35 residues in the N-terminal region of MPP6 are critical for its binding to MTR4. On the other hand, all N-terminal truncates as well as full-length MPP6 associated efficiently with the exosome core, and this finding was also in accord with the results of the pulldown experiment (Figure 4E, F, K and L). These results further establish that MPP6 binds to both MTR4 and the core in distinct regions.

Highly conserved residues between yeast and humans are concentrated in the 5–35 region, prompting us to further examine whether these residues, L9, S10, M16, K17, F18 and M19, are important for the association of MPP6 with MTR4 (Supplementary Figure S5A). Alanine substitutions were introduced to make L9A/S10A (LS)- and M16A/K17A/F18A/M19A (MKFM)- mutants. Both mutants failed to associate with MTR4 while they efficiently bound to exosome core components, suggesting that these conserved residues are significant for MTR4–MPP6 interaction (Supplementary Figure S5B). C-terminal deletions did not exhibit any obvious effect on the interaction between MPP6 and either MTR4 or the exosome core, as predicted from the pulldown results (Supplementary Figure S5C–F).

Taken together, these results showed that MPP6 binds both MTR4 and the exosome core in distinct regions, and is in excellent agreement with the recently resolved structure of the reconstituted exosome complex accompanied by both MPP6 and MTR4 (101).

### MPP6 sustains the interaction between the core and MTR4

A previous report suggested that the interaction between MTR4 and the exosome core is impaired by MPP6-depletion in human cells (135). In that study, MPP6-depletion also caused a great reduction in RRP6-MTR4 interaction, and the authors concluded that MPP6 was an auxiliary factor for the RRP6-MTR4 interaction. However, their complex formation analyses were performed on forcibly expressed proteins, making it difficult to evaluate the precise contributions of RRP6 and MPP6 to maintaining the MTR4-core interaction.

**Figure 5.**
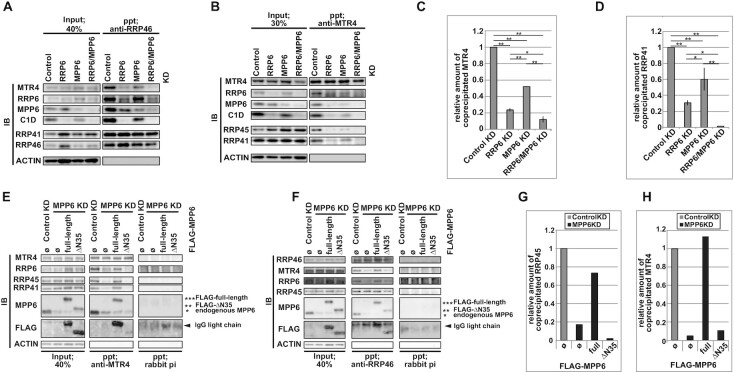
Contribution of RRP6 and MPP6 to maintain the MTR4-core interaction. (**A**–**D**) Immunoprecipitation (IP) analyses to assess the effect of either RRP6 KD, MPP6 KD, or RRP6/MPP6 KD on the MTR4-core interaction. (**E**–**H**) N35 of MPP6 is essential to maintain the interaction between MTR4 and the core. (A), (B) IP experiments were performed on nuclear extracts from HeLa cells deprived of factors noted above the panels using the anti-RRP46 antibody in (A) and the anti-MTR4 antiserum in (B). In these experiments, since the high background of the immunoblotting analyses derived from rabbit antibodies used during the immunoprecipitation step made it difficult to distinguish the genuine RRP6 signal, we used C1D to confirm the RRP6 abundance. Since RRP6 binds to C1D at a 1:1 ratio, which is essential for its stability, C1D should exhibit similar stoichiometric behavior to RRP6 in both the cellular extracts and purified complexes. (C), (D) Quantification of two independent anti-RRP46 IP experiments and anti-MTR4 IP experiments, including (A) and (B), respectively. The ratio of either MTR4 to RRP46 in (C) or of RRP41 to MTR4 in (D) in each precipitate was normalized by the respective ratio in each input extract and by the value of the Control KD sample to render the relative coprecipitated amount of the protein in each sample. Bars and error bars denote mean values ± SD. Statistical analysis was performed using Holm test following one-way ANOVA. *P < 0.05, **P < 0.01, *ns*: not significant. (E, F) IPs were performed on nuclear extracts using the anti-MTR4 antiserum in (E) and using the anti-RRP46 antibody in (F). Extracts were prepared from HeLa Flp-In T-REx cells under the conditions described in the upper part of the panel. Ø represents FLAG only. Rabbit pre-immune antiserum was used as a control in the IP step. (G, H) Quantification of (E) and (F), respectively. The ratio of either RRP45 to MTR4 in (E) or of MTR4 to RRP46 in (H) in each precipitate was normalized by the respective ratio in each input extract and by the value of the Control KD FLAG only expressing sample.

To elucidate the extent to which either endogenous protein, RRP6 or MPP6, contributes to the MTR4-core interaction *in vivo*, we performed immunoprecipitation experiments using antisera against endogenous MTR4, RRP46 (Figure 5A–D, Supplementary Figure S6A and B), and RRP45 (Supplementary Figure S6C and D). We observed a significant reduction in MTR4-core binding under the MPP6 KD condition, as well as the same strong decrease in MTR4-core interaction by RRP6 KD that has been described before (Figure 5A-D) (46), while DIS3-depletion provoked a moderate increase in MTR4-containing exosomes rather than an attenuated MTR4-core interaction (Supplementary Figure S6A–D). We failed to detect DIS3 in either MTR4- or exosome core-precipitates, and thus could not estimate the effect of depleting each exosome cofactor on the DIS3-core interaction (Supplementary Figure S6A-D). This difficulty likely reflects, as previously demonstrated, the low affinity of DIS3 to the exosome core (19,136).

Exogenously expressed full-length MPP6, but not ΔN35, rescued the reduced MTR4-core interaction elicited by endogenous MPP6-deprivation (Figure 5E–H), indicating that the interaction between the core and MTR4 is maintained via binding of MPP6’s N-terminal region to MTR4. We also observed a larger decrease in MTR4–core interaction in RRP6 KD cells than resulted from MPP6 KD alone, but the strongest decrease was observed under the RRP6/MPP6 KD condition (Figure 5A–D). Knocking down either RRP6 or MPP6 often influenced the abundance of exosome components, which makes it difficult to precisely quantify co-precipitated factors. To address this problem, we employed a GST-MTR4 pulldown assay and observed that MPP6-depletion leads to a decrease in the MTR4–core interaction, albeit to a lesser extent than resulted from RRP6-deprivation, thereby confirming our immunoprecipitation results (Supplementary Figure S6E and F). Recent *in vitro* reconstitution experiments suggest that RRP6 and MPP6 contribute to the recruitment of MTR4 to the core cooperatively, but to different degrees, which is in accordance with our results (100). Together, these results demonstrate that MPP6, albeit to a lesser extent than RRP6, sustains the interaction between MTR4 and the core in human cells.

### The interaction with MTR4 is crucial for MPP6’s stimulatory function on both RRP6 and DIS3 to degrade poly(A)^+^ substrates

**Figure 6.**
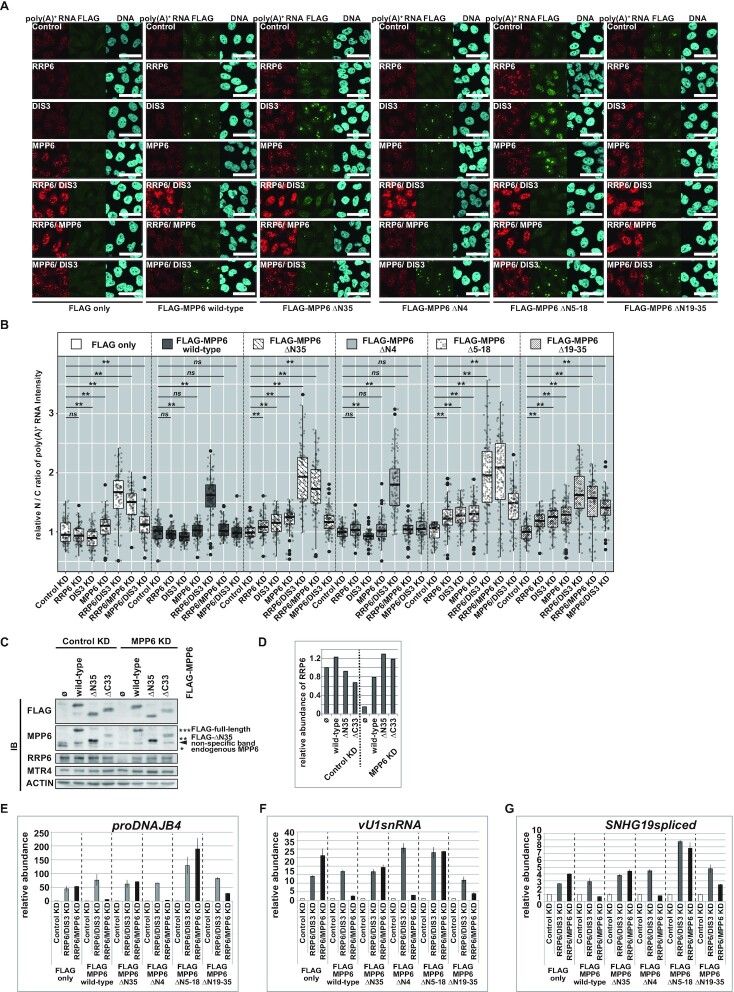
MTR4 binding is essential for MPP6 to function redundantly with RRP6 to mediate poly(A)^+^ substrate decay. (**A**, **B**) Poly(A)^+^ FISH analysis of HeLa Flp-In T-REx cells expressing FLAG-MPP6 mutants. (**C**, **D**) Exogenously expressed *Δ*N35, as well as ΔC33, maintains the expression level of RRP6 in MPP6 KD cells. (**E**–**G**) RT-qPCRs to evaluate the functional significance of each sub-region within the N-terminal of MPP6 in degrading individual poly(A)^+^ substrates. (A) Cell lines tested are stated at the bottom of the panels and depleted factors in the panels. Scale bar = 50 μm. (B) Quantification of (A). Relative nuclear/cytoplasmic (N/C) ratio of poly(A)^+^ FISH signal normalized to the mean value of Control KD cells within each cell line. Statistical analysis was performed using Steel test following Kruskal–Wallis test. *P < 0.05, **P< 0.01, *ns*: not significant, *n* = 100. (C) Immunoblot analysis of nuclear extracts from HeLa Flp-In T-REx cell lines expressing FLAG-MPP6 mutants under Control KD and MPP6 KD conditions. Ø represents FLAG only. (D) Quantification of RRP6 in (C). Values shown are relative abundances of RRP6 normalized by ACTIN and by the value of Control KD FLAG only expressing sample. (E–G) PCR was performed on dT_25_-primed cDNA synthesized using total RNA from whole cells to quantify (E) *proDNAJB4*, (F) *vU1snRNA* and (G) spliced *SNHG19*. Conditions are indicated at the bottom. Values shown are relative amounts of each transcript normalized by *GAPDH* and by the value of the Control KD sample within each cell line.

Next, we attempted to clarify the functional significance of the MPP6–MTR4 interaction in nuclear exosome-mediated poly(A)^+^ substrate decay. The effect of each mutation on MPP6’s stimulatory function of bulk poly(A)^+^ substrates was examined first (Figure 6A and B, Supplementary Figure S7A). All expressed RNAi-resistant MPP6 mutants exhibited similar subcellular localization and were found to be concentrated mainly in the nucleolus, as observed in a previous study (Figure 6A, Supplementary Figure S7A) (33).

While the wild-type and ΔN4 MPP6s effectively restored nuclear poly(A)^+^ substrate accumulation under MPP6 KD conditions, the ΔN35, ΔN5–18 and Δ19–35 mutants, as well as LS and MKFM, did not rescue poly(A)^+^ RNA accumulating phenotypes (Figure 6A and B, Supplementary Figure S7A). Both ΔN35 and ΔC33, as well as wild-type MPP6, efficiently restored decreased RRP6 protein levels caused by endogenous MPP6-depletion (Figure 6C and D). Thus, a prominent phenotype observed in MPP6 N-terminal mutant-expressing cells under MPP6 KD conditions does not seem to be derived from the co-depletion of RRP6 protein. This confirms the direct role of MPP6, as well as the functional significance of the N-terminal region of MPP6, in stimulating both RRP6 and DIS3, thereby substrate decay in human cells as described in recent *in vitro* experiments (99–102).

The mild nuclear poly(A)^+^ RNA accumulation observed in MPP6/DIS3 KD and MPP6 KD 4M-expressing cells indicates that MPP6 is essential for the optimal substrate decay conducted by RRP6, despite the residual activity of RRP6 even in the absence of MPP6 (Figure 3A–C, Supplementary Figure S4C and D). The ΔN35, ΔN5–18 and Δ19–35 mutants, as well as LS and MKFM, failed to rescue the MPP6/DIS3 KD-elicited phenotype, while the wild-type and the ΔN4 mutant both efficiently restored it (Figure 6A and B, Supplementary Figure S7A), suggesting that MTR4, recruited by MPP6, can stimulate RRP6-mediated poly(A)^+^ substrate decay. These MPP6 mutants lack the capacity to bind MTR4 and failed to restore the nuclear poly(A)^+^ RNA accumulating phenotype found in RRP6/MPP6 KD cells, suggesting that binding to MTR4 is also essential for MPP6 to exert its functional redundancy with RRP6 (Figure 6A, E–G, Supplementary Figure S7A–D). We also observed a considerable nuclear accumulation of poly(A)^+^ RNA when either ΔN35, ΔN5–18, Δ19–35, LS or MKFM was expressed in RRP6 KD cells, as well as in DIS3 KD cells, even without depleting endogenous MPP6 protein, which further supports that the importance of MTR4 recruitment by MPP6 for a stimulatory function of MPP6 on both RRP6 and DIS3 (Figure 6A and B, Supplementary Figure S7A).

In these experiments, we observed the exclusion of FLAG-MPP6s from the nucleolus when RRP6 was depleted (Figure 6A). However, this phenomenon was not derived from a decrease in the expression of FLAG-fused proteins and was induced irrespective of whether the expressed FLAG-fused MPP6 was wild-type or mutant-type (Supplementary Figure S7E, Figure 6A and B). In addition, poly(A)^+^ substrate accumulation was observed even when FLAG-MPP6 mainly resided in the nucleolus—e.g. under MPP6- and DIS3-depleted conditions—implying that the overall change in the localization of exogenously expressed MPP6 is not a direct cause of a perturbation in exosome activity (Figure 6A and B); however, it is possible that the increased nucleoplasmic quantity of the loss-of-function mutants contributes to a severer phenotype.

The amino acid residues involved in the contact of MPP6 with MTR4’s two RecA domains are mostly concentrated within the 5–18 domain rather than in the 19–35 domain, which might explain why a stronger phenotype was exhibited by Δ5–18-expressing cells than was observed in Δ19–35 cells (Figure 6A, B and E–G) (100,101). In contrast to these observations, which demonstrated the significance of the N-terminal region of MPP6 for substrate decay, we did not find any obvious defects in MPP6 function when we performed similar experiments for ΔC11 and ΔC33 MPP6s, which could efficiently bind to both MTR4 and the exosome core (Supplementary Figure S7F–I, Figure 4E–H, Supplementary Figure S5C–F).

### Functional nonequivalence between MTR4s recruited by MPP6 and by RRP6 in human cells

Our data so far clearly demonstrate that the physical association of MPP6 with MTR4 is essential for its functional redundancy with RRP6. However, since RRP6 is a nuclease as well as a bridge molecule between MTR4 and the core, in the above experiments it was difficult to estimate the genuine contribution of MTR4 recruitment via RRP6 with respect to substrate decay. Recent *in vitro* reconstitution analyses have suggested that the binding surface provided by RRP6-C1D is sufficient for bound MTR4 to form a channel-threading path to DIS3 without the aid of MPP6 (99–102). Accordingly, a catalytically inert RRP6 can facilitate MTR4-dependent decay of the substrates by the nucleolytic activity of DIS3 (100,101). If such a functional equivalence between RRP6-bound MTR4 and MPP6-bound MTR4 is also the case *in vivo*, expressing a catalytically inert RRP6 should effectively restore the poly(A)^+^ substrate accumulation derived from RRP6/MPP6 KD.

**Figure 7.**
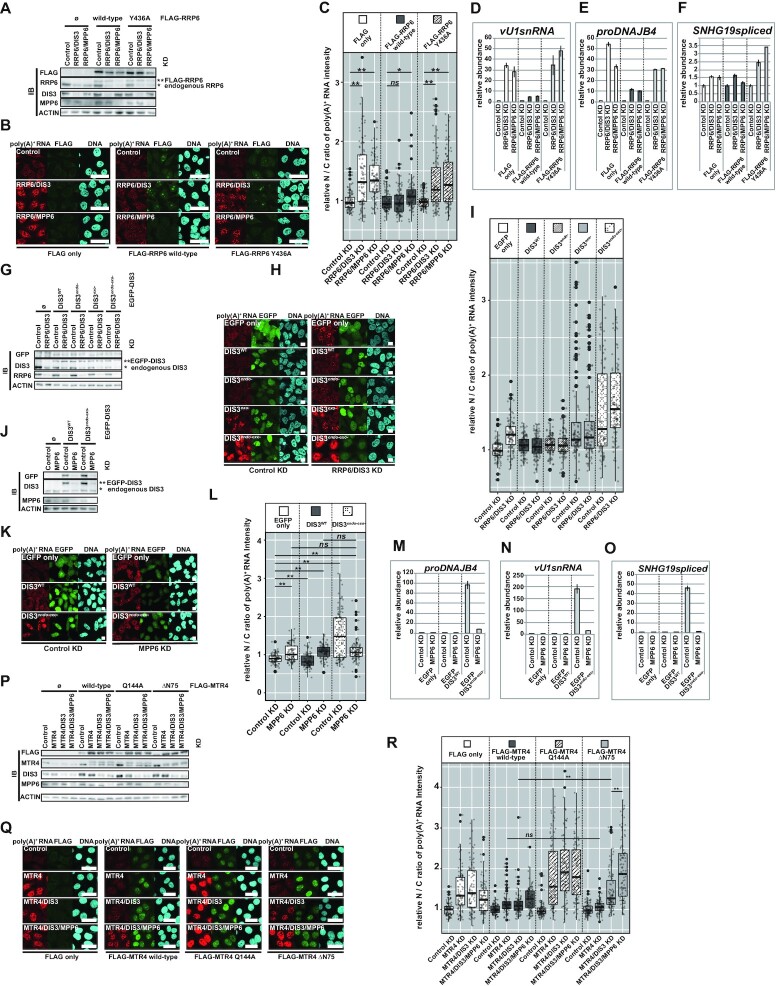
Functional differences between MPP6-recruited MTR4 and RRP6-recruited MTR4 in human cells. (**A**–**F**) Y436A, a catalytically inactive RRP6, cannot substitute for the function of RRP6 in the decay of poly(A)^+^ substrates where RRP6 and MPP6 function redundantly. (**G**–**I**) A DIS3*^endo-exo^^−^*, a DIS3 mutant that lacks both endonuclease and exonuclease activities, exhibits a nuclear poly(A)^+^ RNA accumulating dominant-negative phenotype. (**J**–**O**) Nuclear poly(A)^+^ RNA accumulation induced by the expression of DIS3*^endo-exo^^−^* is abrogated by MPP6 KD. (**P**–**R**) RRP6–MTR4 interaction is indispensable for optimal substrate decay by RRP6. (A, G, J, P) Immunoblot analysis of nuclear extracts. Cell lines and depleted factors are indicated above the panels. Ø means FLAG only in (A) and (P), EGFP only in (G) and (J). (B, H, K), Q) Poly(A)^+^ FISH experiments. Conditions of cell lines and depleted factors are stated both in and at the bottom of the panels. Scale bar = 50 μm in (B) and (Q), and 10 μm in (H) and (K). (C, I, L, R) Quantification of (B), (H), (K), (Q), respectively. Relative nuclear/cytoplasmic (N/C) ratio of poly(A)^+^ FISH signal normalized to the mean value of Control KD cells within each cell line in (C) and (R), and to the mean value of Control KD EGFP only expressing cells in (I) and (L). Statistical analysis was performed using Kruskal–Wallis test followed by Steel test in (C) and Steel-Dwass test in (L, R). *P < 0.05, **P < 0.01, *ns*: not significant, *n* = 100. (D–F, M–O) PCR was performed on dT_25_-primed cDNA synthesized using total RNA from whole cells to quantify *proDNAJB4* in (D) and (M), *vU1snRNA* in (E) and (N), and spliced *SNHG19* in (E) and (N). Values shown are relative amounts of each transcript normalized by *GAPDH* and by the value of Control KD sample within each cell line in (D)–(F), and by the value of Control KD EGFP only expressing cells in (M)–(O).

To examine this hypothesis, we first constructed FLAG-fused RNAi-resistant RRP6s, wild-type and a catalytically inert mutant, Y436A, and tested their ability to support the substrate decay attributed to DIS3 (Figure 7A–F) (27). When stably expressed in HeLa Flp-In T-REx cells, wild-type RRP6 readily dissolved nuclear poly(A)^+^ substrate accumulation derived not only from RRP6/DIS3 KD, but also from RRP6/MPP6 KD. On the other hand, expressed Y436A rescued neither the RRP6/DIS3 KD- nor the RRP6/MPP6 KD-induced phenotype. However, Y436A could restore the RRP6 KD-derived reduction of MTR4-core association to a comparable extent as the wild-type (Supplementary Figure S8A and B). A similar result was observed when expressing another catalytically inert mutant, E315A (Supplementary Figure S8C) (27). These results suggest that the functional redundancy of RRP6 with MPP6 observed in our poly(A)^+^ substrate decay assays is related to its nuclease function, rather than it being a bridge molecule between MTR4 and the core. In other words, DIS3 cannot degrade nucleoplasmic poly(A)^+^ substrates without the aid of MPP6 even when a considerable amount of MTR4 is recruited to the core via its interaction with RRP6 [Supplementary Figure S8D, (x) MPP6 KD RRP6*Δ*exo].

To further elucidate the indispensability of MTR4 recruited by MPP6 for poly(A)^+^ substrate decay by DIS3, we used the dominant-negative phenotype exhibited by a DIS3 mutant lacking both exo- and endonuclease activities (37,137). Because DIS3 is a processive exonuclease, when this activity does not function properly, the 3’end of the substrate should be stuck to this enzyme. Such a substrate can be cut out and liberated again by the endonuclease activity of DIS3. If the decay of the substrate depends solely on DIS3, it should therefore be stabilized when the exonuclease activity of DIS3 is continuously perturbed. On the other hand, if this substrate can be processed not only by DIS3 but also by RRP6, then the liberated substrate can bind to RRP6 and be decayed even if the exonuclease activity of DIS3 is absent. Since, as we have observed, poly(A)^+^ substrates are degraded redundantly by RRP6 and DIS3, we expected that inhibiting only the exonuclease activity of DIS3 would not stabilize the substrate, but preventing both exo- and endonuclease activity should do so. In agreement with this hypothesis, we observed a robust nuclear poly(A)^+^ RNA accumulation by transiently expressing FLAG-fused DIS3*^endo-exo^^−^*, while an equivalently dominant-negative phenotype was not provoked by expressing DIS3^WT^, DIS3*^endo^^−^*, or DIS3*^exo-^* (Supplementary Figure S8E). Such stabilization of nucleoplasmic substrates by expressing DIS3*^endo-exo^^−^* was also demonstrated in a previous report (37,137).

Analogous results were obtained in established HeLa Flp-In T-REx cell lines that stably expressed RNAi-resistant N-terminal EGFP tagged DIS3 mutants, except for EGFP-DIS3*^exo^^−^*, which elicited a subtle dominant-negative poly(A)^+^ RNA accumulating phenotype (Figure 7G-I, Control KD). These results implied that N-terminal EGFP tagging somewhat disturbs DIS3’s endonuclease activity. Depletion of MPP6 from EGFP-DIS3*^endo-exo^^−^* expressing cells resulted in diminished nuclear poly(A)^+^ substrate accumulation at a level comparable to that induced solely by MPP6 depletion (Figure 7J–O). We also observed a markedly reduced expression of EGFP-DIS3 under MPP6 KD conditions, regardless of whether the expressed DIS3 was the wild-type or DIS3*^endo-exo^^−^* mutant. However, even when doxycycline was added to equalize the expression level of the EGFP- DIS3*^endo-exo^^−^* mutant to the levels found in MPP6-depleted cells, the poly(A)^+^ RNA accumulating phenotype was still observed (Supplementary Figure S8F–I). Hence, these results also suggest that substrates are unable to efficiently reach DIS3 in the absence of MPP6 even though a considerable MTR4-core interaction is sustained by RRP6-MTR4 binding [Supplementary Figure S8D, compare between (v) MPP6 KD DIS3*^endo-exo^^−^* and (ix) DIS3*^endo-exo^^−^*; poly(A)^+^ substrates are not tethered to DIS3 in MPP6 KD DIS3*^endo-exo^^−^* cells, allowing them to be decayed by RRP6, although not fully decayed in the absence of MPP6]. These results illuminate the significance of MPP6 for tying MTR4 and DIS3 into a functional form.

We also tried to clarify the extent that RRP6-bound MTR4 contributes to the substrate decay executed by RRP6. Even though MTR4 binding to MPP6 is still essential for the optimal function of RRP6, mild poly(A)^+^ substrate accumulation was observed in both MPP6/DIS3 KD U2OS and MPP6 KD 4M-expressing cells, thereby indicating that RRP6-bound MTR4 can somewhat support the poly(A)^+^ substrate decay conducted by RRP6 without the aid of MPP6 (Figure 3A–C, Supplementary Figure S4A and C). To confirm the functional significance of the MTR4-core complex bridged via RRP6 in RRP6-executed substrate decay, we assessed the activity of the ΔN75 MTR4 mutant, an N-terminal truncated MTR4 that fails to interact with RRP6. U2OS Flp-In T-REx cell lines expressing FLAG-fused and RNAi-resistant MTR4 of either wild-type, a helicase-null Q144A mutant, or ΔN75 were established (Figure 7P–R, Supplementary Figure S8J and K) (98). As demonstrated in a related U2OS cell line (Figure 2A–C), remarkable nuclear poly(A)^+^ RNA accumulation was induced by the depletion of endogenous MTR4 in U2OS parental Flp-In T-REx cells. The ΔN75 mutant restored nuclear poly(A)^+^ RNA accumulation derived from the depletion of endogenous MTR4 as efficiently as wild-type MTR4 did, while Q144A rather enhanced the phenotype, indicating that this truncation does not significantly disturb the helicase activity of MTR4. These results also confirm the dispensability of RRP6-bound MTR4 for substrate decay when the path from MTR4 to DIS3 supported by MPP6 is available. MTR4/DIS3 KD in ΔN75-expressing cells provoked mild nuclear poly(A)^+^ RNA accumulation, while the same knockdown in wild-type MTR4 expressing cells resulted in no obvious accumulation, suggesting that RRP6-conducted poly(A)^+^ substrate decay is attenuated when the RRP6–MTR4 interaction is absent [Figure 7P-R, Supplementary Figure S8D, (vi) DIS3 KD *Δ*N75 MTR4]. Knocking down of MPP6 in MTR4/DIS3-depleted ΔN75-expressing cells further enhanced poly(A)^+^ substrate accumulation, indicating that considerable activity is indeed sustained by MTR4 recruitment through MPP6 even without the RRP6-MTR4 interaction. Thus, our data demonstrate that RRP6 requires MTR4 to be bound to both RRP6 and MPP6 for optimal function. Summarily, these data suggest a nonequivalence of MTR4 recruited by RRP6 to MPP6-bound MTR4.

### Genome-wide analysis of exosome substrates using a highly efficient RNA extraction method

**Figure 8.**
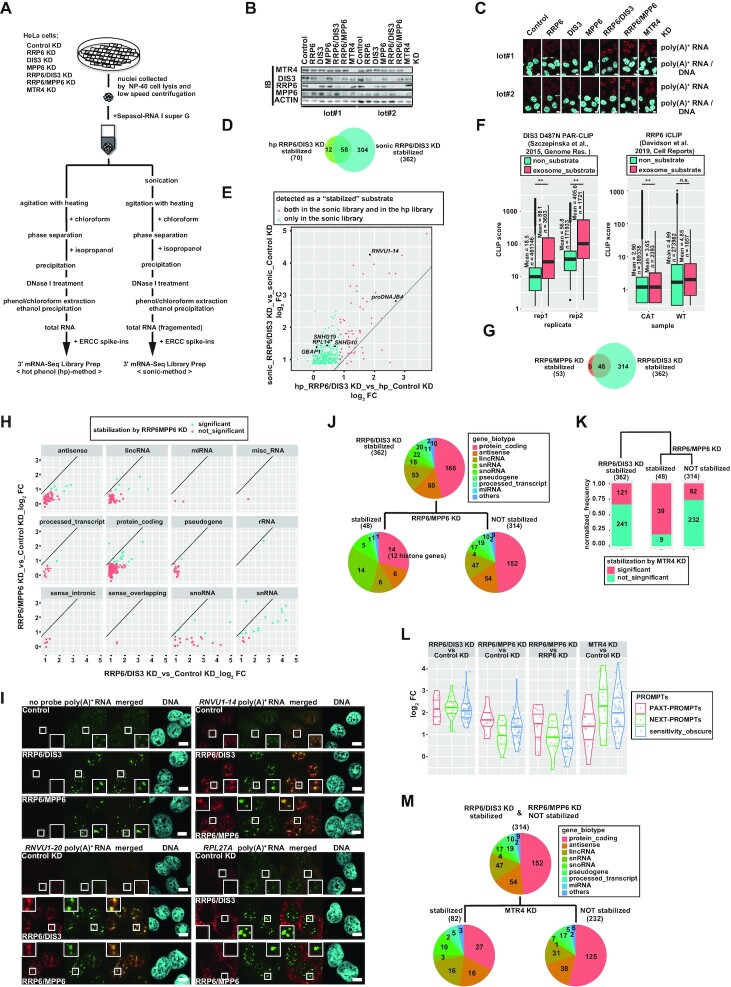
Genome-wide analysis of poly(A)^+^ substrates redundantly decayed by RRP6 and MPP6. (**A**–**C**) Preparation of NGS samples. (**D**, **E**, **G**, **H**, **J**–**M**) Analyses of the exosome substrates based on their extractability and sensitivity to the exosome components. (**F**) Reanalysis of published CLIP data. (**I**) Visualization of the substrates using specific FISH probes. (A) Schematic diagram of the preparation of NGS samples. (B, C) Checking NGS samples by immunoblotting in (B) and by poly(A)^+^ FISH in (C). (D, G) Venn diagram view to compare the stabilized substrates between the hot-phenol (hp)- and the sonic-RRP6/DIS3 KD samples in (D), and between the RRP6/DIS3 KD and the RRP6/MPP6 KD sonic samples in (G). Numeric values indicate the number of substrates in each category. (E, H) Plots of log_2_ fold change (log_2_ FC) for each substrate among the sonic RRP6/DIS3 KD stabilized substrates. In (E), log_2_ FC induced by RRP6/DIS3 KD in the hp samples was plotted to that in the sonic samples. Read coverages to the regions of the substrates noted in black letters are shown in supplementary Figure S9H. In (H), log_2_ FC induced by RRP6/DIS3 KD was plotted against that induced by RRP6/MPP6 KD in the sonic samples, for each gene-biotype category. The line in the figure shows *y* = *x*. (F) Published DIS3 PAR-CLIP and RRP6 iCLIP data were compared between the sonic RRP6/DIS3 KD stabilized substrates and sonic RRP6/DIS3 KD NOT stabilized substrates, i.e. between ‘exosome substrate’ and ‘non-substrate’. CLIP scores were calculated per gene as described in the MATERIAL AND METHODS section. For DIS3 PAR-CLIP data, scores were compared within each replicate, rep1 and rep2. CAT and WT indicate the catalytically inert and wild-type RRP6, respectively. For CAT and WT, replicates are shown in the merged data. Statistical analysis was conducted using the Wilcoxon rank sum test with continuity correction. (**I**) *RNVU1-14*, *RNVU1-20* and *RPL27A* were visualized using DIG-labeled RNA probes simultaneously with poly(A)^+^ RNA. Factors depleted are described in panels. Magnified pictures are shown in the insets. Scale bar = 10 μm. (J, M) Pie chart showing the breakdown of gene-biotype. In (J), the entire RRP6/DIS3 KD stabilized substrates and its derived subsets, the RRP6/MPP6 KD stabilized and NOT stabilized, were dissected. In (M), the RRP6/MPP6 KD NOT stabilized subset extracted from the RRP6/DIS3 KD stabilized substrates and its derived subsets, the MTR4 KD stabilized and NOT stabilized, were dissected. Others represent the sum of the genes categorized as encoding sense intronic RNAs, sense overlapping RNAs, miscellaneous RNAs (misc RNAs), and ribosomal RNAs (rRNAs). Numeric values indicate the number of substrates in each category. (K) Sensitivity to MTR4 of the RRP6/MPP6 KD stabilized and the NOT stabilized subclasses. Numeric values indicate the number of substrates in each category. (L) Effect of RRP6/MPP6 KD on the stabilization of PAXT- and NEXT-PROMPTs. Log_2_ FC induced for each PROMPT under the conditions described at the top of the panels is shown in violin plots. Only those PROMPTs whose expression was stabilized in our RRP6/DIS3 KD samples were analyzed.

Although our results so far have proven an MPP’s stimulatory function on the nuclear exosome, whether its function covers whole substrates of the nuclear exosome or is limited to a subset with a specific feature remains to be elucidated. To answer this question, we next performed a genome-wide analysis on nuclear poly(A)^+^ RNAs under Control KD, RRP6 KD, DIS3 KD, MPP6 KD, RRP6/DIS3 KD, RRP6/MPP6 KD and MTR4 KD conditions (Figure 8A–C). Since poly(A)^+^ foci emerged upon exosome perturbation were tightly associated with PABPN1 and were reminiscent of persistent aggregates with poor extractability—as observed for disease-causing PABPN1-aggregates—for the first step of this experiment, we modified the RNA extraction and cDNA library preparation methods to achieve thorough transcriptome profiling (Supplementary Figure S2E) (138,139).

The acid guanidinium thiocyanate–phenol–chloroform (AGPC) extraction method with heating has been reported to improve the efficiency of extracting RNA from cells, especially for nuclear body-associated architectural noncoding RNAs (140). However, it was not yet known whether this method is also sufficient for extracting transcripts within poly(A)^+^ aggregates induced by exosome inhibition. Thus, we first tried a harsh extraction method, employing sonication and heating (hereafter the sonic-method), and examined whether this method further elevated the efficiency of extracting representative substrates of the nuclear exosome (Supplementary Figure S9A and B). As previously reported, a heating-employing AGPC method [hereafter the hot-phenol (hp) method] greatly enhanced the efficiency of extracting *NEAT1_2*, an architectural transcript associated with paraspeckles (Supplementary Figure S9B) (140). This method seemed also to be effective for extracting *SNHG19* and *SNHG10*, substrates of the exosome, albeit to a much milder extent (47,65–68). When samples underwent sonication in addition to heating, the relative abundance of *SHNG19* and *SNHG10* within the total RNA was further elevated, suggesting that sonication is effective for transcripts with extremely low extractability (Supplementary Figure S9A).

Despite enhanced efficiency in extracting substrates, we found that the sonication step led to the fragmentation of extracted transcripts, which manifested as a low RNA integrity number (Supplementary Figure S9C, left panels), and a decrease in the abundance of *NEAT1_2* and *PGK1* (Supplementary Figure S9A and B). To minimize the effect of such fragmentation on the preparation of cDNA libraries, and hence to avoid biasing the resulting quantification, we generated a cDNA library of sequences limited to narrow regions upstream of the 3’ A-tail. The size distributions of the resulting cDNA libraries were similar between libraries, including one prepared from intact total RNA and one prepared from total RNA fragmented by sonication (Supplementary Figure S9C, right panels).

Synthesized cDNA libraries were sequenced and acquired sequences were trimmed and mapped to hg19. Because of the narrow window for our cDNA libraries, it is inappropriate to normalize read counts using gene length. However, counting reads using ‘gene’ features that includes both introns and exons can easily detect false-positive substrates derived from long introns covered with low-density reads (Supplementary Figure S9D–F). Therefore, we focused on ‘exon’ features in this study to avoid the noise and simplify interpretation. Counting data of mapped reads were then subject to DESeq2 differential expression analysis (Supplementary Figure S9G) (119). ENSEMBL GRCh37.75 genes that showed adjusted *P*-values <0.05 and log_2_ FC >0.85 were classified as significantly upregulated genes whose products were stabilized (hereafter, referred to as ‘stabilized substrates’). Most of the identified RRP6/DIS3 KD-induced stabilized substrates (RRP6/DIS3 KD vs Control KD stabilized: RRP6/DIS3 KD stabilized) in samples prepared using the hp-method were also identified as RRP6/DIS3 KD stabilized substrates in sonic-method samples (Figure 8D, Supplementary Figure S9H, *proDNAJB4* and *RNVU1-14*). Additionally, the number of stabilized substrates significantly increased in samples prepared using the sonic-method compared to those prepared using the hp-method (Figure 8D and E, Supplementary Figure S9H, *SNHG19*, *SNHG10*, *GBAP1* and *RPL14*). Moreover, for the substrates stabilized in both the hp- and sonic-method samples, the fold increase estimates obtained from sonic-method samples tended to be greater than those from the hp-method samples (Figure 8E, Supplementary Figure S9H, *RNVU1-14* and *proDNAJB4*). These results suggest that sonication can strongly improve the extraction efficiency of transcripts stabilized under impaired nuclear exosome conditions.

By analyzing the samples prepared using the sonic-method, we classified RRP6/DIS3 KD stabilized substrates, corresponding to the entire exosome poly(A)^+^ substrates within the scope of our analysis, following the sensitivity to either RRP6 or DIS3. Substrates were sorted into four classes as follows: class1: substrates specifically degraded by RRP6, class2: substrates specifically degraded by DIS3, class3: substrates stabilized by the sole depletion of either RRP6 or DIS3, and class4: substrates that RRP6 and DIS3 redundantly degrade in a manner similar to the observed degradation of bulk poly(A)^+^ substrates in our experiments (Supplementary Figure S9I, Supplementary Table S2). Most substrates (357 of 362) were sorted into class4, suggesting a pervasive functional redundancy between RRP6 and DIS3 in nuclear substrate decay. Relatively few substrates were sorted into class1 and class2 and none were sorted into class3 with our applied filter. In class1, we identified *RNA45SN5*, which is transcribed into 47S rRNA precursors, as observed in previous analyses (34–36). Substrates sorted into class2 were also identified as nucleoplasmic targets for DIS3 in a previous study (Supplementary Figure S9J) (36), although we do not yet know why these substrates are not susceptible to RRP6 in the absence of DIS3. Most of the substrates reported in previous analyses were enriched in class4 (including *SNHG*s, *proDNAJB4*, *vU1snRNA*s, *RNU5E*s, pseudo *U2snRNA*s, *U4ATAC*, *PCF11* IPA transcripts, *DNAJC30*, *c12orf57*, *MIR17HG*, *SNORD83A*, *LINC01311*, *RNF139-AS*, etc., Supplementary Table S2) (47,65,67,68,76,80,81,86,141,142). Also, reanalyzing the previous CLIP data revealed a significant physical association of DIS3 to our identified substrates, although the binding of RRP6 to the substrates was less obvious (Figure 8F, see also DISCUSSION section) (36,37). The similarities of the identified substrates to those found in previous analyses increase our confidence in the validity of our analysis.

### MPP6 acts on a specific subset of exosome-sensitive substrates

Concerning RRP6/MPP6 KD stabilized substrates, most did not fall into a class where stabilization occurred by depleting only one of RRP6 or MPP6 (48 out of 53 substrates were stabilized only when both RRP6 and MPP6 were depleted, Supplementary Figure S9K), suggesting that these substrates are prone to be degraded in an RRP6 and MPP6 redundant manner. More specifically, we infer from our experimental data that the decay of these substrates is redundantly conducted by MPP6-assisted RRP6 and DIS3, albeit that the decay by RRP6 does not depend exclusively on MPP6. Actually, most of these substrates were also stabilized by RRP6/DIS3 KD (48 out of 53 RRP6/MPP6 KD stabilized, Figure 8G). On the other hand, RRP6/MPP6 KD stabilized substrates accounted for only a limited subset of RRP6/DIS3 KD stabilized ones corresponding to the entire poly(A)^+^ exosome substrates in our analysis window (48 out of 362 RRP6/DIS3 KD stabilized, Figure 8G). The overall RNA stabilization tended to be smaller in RRP6/MPP6 KD samples than in RRP6/DIS3 KD samples. However, at the same time, we observed that some substrates were greatly stabilized by both RRP6/DIS3 KD and RRP6/MPP6 KD, while others were strongly stabilized by RRP6/DIS3 KD but not by RRP6/MPP6 KD. Such a discrepancy in the stabilization between the RRP6/DIS3 KD and RRP6/MPP6 KD conditions was most prominent between snRNAs and snoRNAs (Figure 8H). Thus, a poor variety in RRP6/MPP6 KD stabilized substrates did not seem to be necessarily caused by a smaller stabilization in general, but rather reflected the specificity of RRP6 and MPP6 for their substrates.

The robust nuclear poly(A)^+^ RNA accumulation induced by RRP6/MPP6 KD implies that this specific RRP6/MPP6 KD-susceptible subset is prone to form poly(A)^+^ aggregates. Furthermore, poly(A)^+^ substrates that are stabilized not only under RRP6/DIS3 KD condition but also under RRP6/MPP6 KD condition are plausible components of poly(A)^+^ aggregates elicited by exosome inhibition. Importantly, most of the identified RRP6/MPP6 KD stabilized substrates met this criterion (Supplementary Figure S9L). In addition, low extractability was observed across the entire substrates, but was particularly pronounced for RRP6/MPP6 KD stabilized subsets (Supplementary Figure S9M). Furthermore, we visualized several exosome substrates identified in our NGS analysis and observed that RRP6/MPP6 KD stabilized substrates, *RNVU1-14* and *RNVU1-20*, co-localized with poly(A)^+^ foci either in RRP6/DIS3 KD or RRP6/MPP6 KD cells, whereas as *RPL27A*, an RRP6/MPP6 KD NOT stabilized substrate, did not (Figure 8I, Supplementary Table S2). The incorporation within the exosome-KD derived poly(A)^+^ aggregates was also demonstrated for other RRP6/MPP6 KD stabilized substrates, such as *SNHG19* and *proDNAJB4*, in previous studies (83). These findings support the hypothesis that the RRP6/MPP6 KD-susceptible transcripts comprise a subset of exosome substrates that readily generate poly(A)^+^ aggregates when stabilized.

### Features for RRP6/MPP6 KD stabilized substrates

Next, we attempted to identify the characteristics common to this limited subset of substrates. The analysis of the gene-biotypes of RRP6/MPP6 KD stabilized substrates revealed that noncoding genes were ubiquitously relative to all the identified exosome substrates (Figure 8J). This category includes representative substrates such as *SNHG19, vU1snRNA*, and *proDNAJB4* (Supplementary Table S2). The most concentrated gene-biotype was snRNA. Many of these were annotated as variant snRNAs rather than as authentic snRNAs (13 out of 14; 10 as variant snRNAs and 3 as pseudo snRNA genes). Furthermore, when taking a closer look at protein-coding genes, 12 out of 14 identified genes were histone-coding genes. It has been shown that authentic snRNAs and histone mRNAs, when properly processed, arise as transcripts with non-adenylated 3' ends; however, variant snRNAs and 3’ extended histone mRNAs undergo polyadenylation and enter a distinct pathway from the authentic transcripts (61,80,81,141,143,144). Variant snRNAs and histone-coding genes accounted for more than half of the gene-biotypes found in RRP6/MPP6 KD stabilized substrates. Although ALY/REF, a general mRNA export adaptor recruiting NXF1 exporter onto RNA transcripts, has been shown to facilitate proper 3’ end processing and the nuclear export of histone mRNAs (145), our observed abnormal polyadenylated forms of histone mRNAs were retained in the nucleus to be degraded by the exosome and may follow a different fate from matured ones even after release from tethering mechanisms.

Finally, when RRP6/MPP6 KD stabilized substrates were further sorted based on their sensitivity to MTR4, most exhibited MTR4 KD susceptibility, suggesting that the substrate decay executed redundantly by MPP6-assisted RRP6 and DIS3 is strongly linked to MTR4 activity (Figure 8K). In contrast, MTR4-sensitivity was less characteristic of the whole substrates and of the substrates less susceptible to RRP6/MPP6 KD (RRP6/MPP6 KD NOT stabilized), although it cannot be ruled out that the contribution of MTR4 to the decay of these substrates may have been underestimated because the nuclear substrate retention system in HeLa cells used in these experiments seems not to be so stringent under MTR4 KD condition (Figure 2A-C) (82,83).

Despite such a strong association between MPP6 function and MTR4, a dominant cofactor for the nuclear exosome, a limited range of RRP6/MPP6 KD stabilized substrates implicates some MPP6-irrelevant exosome activities. To examine which cofactor-supported degradation processes MPP6 is involved in, we used the information from PAXT- and NEXT-sensitive annotated PROMPTs provided in the previous report (85). Here our analysis was limited to the PROMPTs that were significantly detected as exosome substrates in our libraries (Figure 8L, 88 out of 5582 total LiftOvered PROMPTs). While it was true that the number of our evaluated PROMPTs was so limited that no statistically significant differences were detected, PAXT-specific PROMPTs (PAXT-RPOMPTs), rather than the NEXT-specific ones (NEXT-RPOMPTs), tended to exhibit a higher sensitivity to RRP6/MPP6 KD (Figure 8L). On the other hand, we did not find such a tendency in RRP6/DIS3 KD or MTR4 KD samples. These results, and the efficient stabilization observed in the decay of *SNHG19*, a typical PAXT-dependent substrate, in RRP6/MPP6 KD cells (Figure 3H, Figure 6G, Supplementary Figure S2J), suggest that MPP6 generally supports PAXT-dependent decay.

In these NGS analyses, we detected some stabilized NEXT-PROMPTs in their adenylated forms. Since these PROMPTs have been annotated as NEXT-sensitive PROMPTs due to their sensitivity to NEXT KD and insensitivity to PAXT KD, regardless of the functional redundancy between NEXT and PAXT to degrade them, such NEXT-PROMPTs may become polyadenylated and subsequently decayed by the PAXT-employed backup system when the NEXT fails to guide them to be degraded (85). That may explain why these NEXT-PROMPTs were detected in their adenylated forms under exosome-inhibited conditions, especially when MTR4, a factor involved in both NEXT and PAXT function, was depleted. We also identified snoRNA as the particularly concentrated gene-biotype in a class that was significantly stabilized by MTR4 KD, but not by RRP6/MPP6KD (Figure 8M). Recently, it was reported that prolonged inhibition of NEXT-exosome activity stabilizes the intron-encoded snoRNAs in an adenylated state, and our RRP6/DIS3 KD experimental condition, as well as MTR4 KD condition, would correspond to this (146). Although adenylated forms of these PROMPTs and snoRNAs might no longer be true NEXT-sensitive targets, the fact that they were less stabilized under RRP6/MPP6 KD conditions than in RRP6/DIS3 KD or MTR4 KD conditions, implies that the NEXT substantially functions to eliminate these adenylated transcripts even when RRP6 and MPP6 are knocked down, by degrading them directly or by promoting an upstream decay process that prevents their appearance.

**Figure 9.**
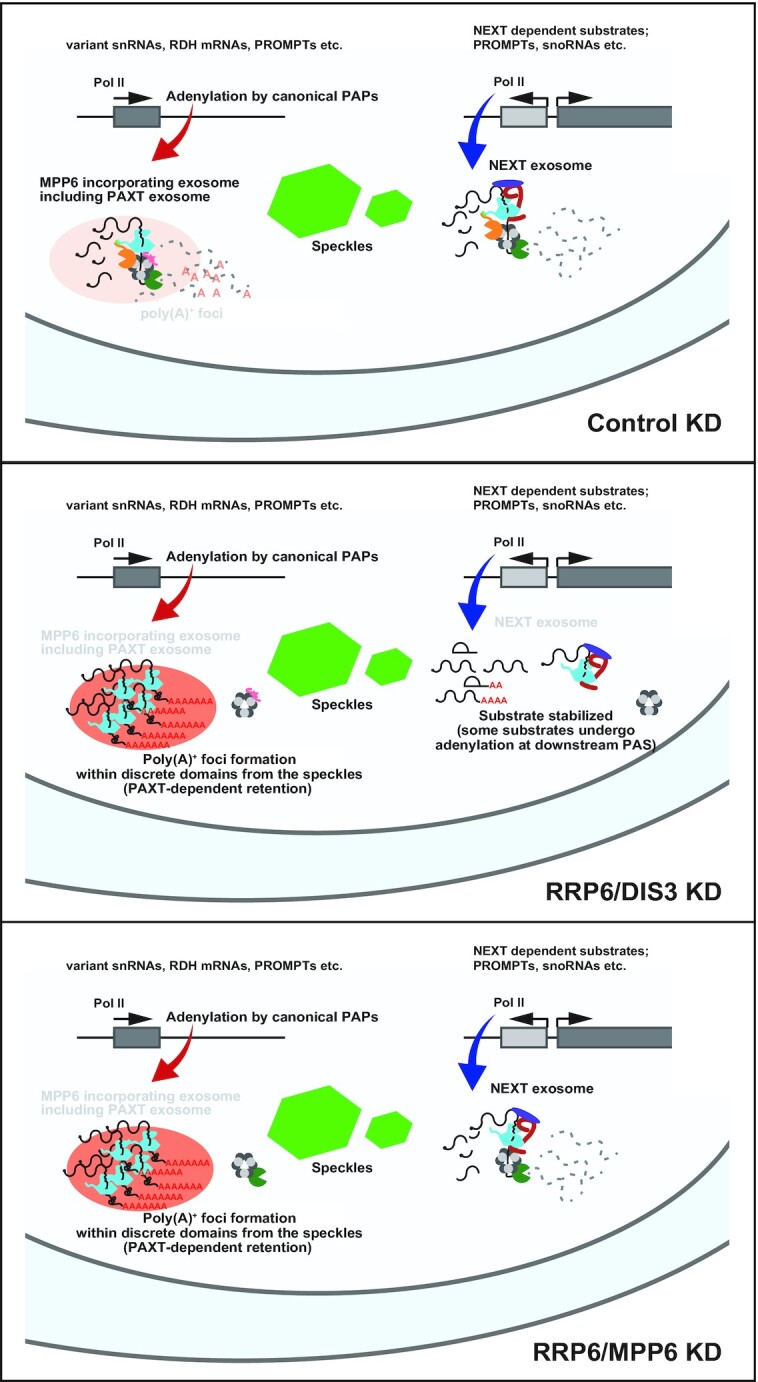
Model of how MPP6 functions in MTR4-dependent poly(A)^+^ substrate decay in the human nucleus. (Control KD) In the control KD nucleus, MPP6 incorporating exosome complexes (including PAXT bound exosome) degrade the adenylated form of transcripts generated via ARS2-mediated transcription termination of short transcription units (TUs), such as variant snRNAs and replication-dependent histone (RDH) encoding mRNAs (see also the DISCUSSION section). MPP6-independent exosome complexes also exist to function in the degradation of other substrates. As a representative example, we describe NEXT associating exosome, which functions in the decay of intron-encoded snoRNAs and NEXT-PROMPTs. (RRP6/DIS3 KD) RRP6/DIS3 KD stabilizes the entire exosome substrates. Poly(A)^+^ substrates, which are decayed under normal conditions by MPP6-incorporating exosome complexes, are stabilized and form MTR4-associated poly(A)^+^ foci that are retained at discrete domains from the nuclear speckles in a PAXT-dependent manner. Some of the NEXT substrates become adenylated when stabilized, probably at polyadenylation signal (PAS) embedded in their 3’ extended sequences. (RRP6/MPP6 KD) Under the RRP6/MPP6 KD condition, poly(A)^+^ clumps similar to those observed in the nucleus of RRP6/DIS3 KD cells emerge, as observed in the RRP6/DIS3 KD nucleus, whereas NEXT-dependent decay remains active.

The preferential action on PAXT-sensitive substrates and the poor involvement in NEXT-dependent substrate decay suggest that the MPP6-incorporated exosome is a specific device for distinct substrate subsets rather than the prevalent complex form (Figure 9).

## DISCUSSION

Although the role of MPP6 in the physical interaction of the exosome core with its critical cofactor, MTR4, has been explicitly proven *in vit*ro (99–102), its actual biological impact on the complex formation and substrate decay has been unclear. In this study, we recapitulate the involvement of MPP6 in the MTR4-core interaction that excellently agrees with the previous *in vitro* analyses, and identify the distinct functions between the MTR4s recruited by RRP6 and MPP6 that have not been captured in those experiments. This study also suggests that MPP6, by forming a specific MTR4-containing exosome complex, conducts the decay of a certain portion of the entire exosome substrate.

The results indicate that MPP6 stimulates both RRP6 and DIS3, two nuclear exosome-relevant nucleases, to redundantly degrade these substrates, albeit to different degrees. Previous studies have demonstrated that DIS3 primarily degrades nucleoplasmic exosome substrates (36,37). The reanalyses of the previous data on our identified substrates indicate the stabilization of these substrates by the rapid DIS3 depletion and a significant binding of our identified substrates to DIS3 but not to RRP6, suggesting that DIS3 decays these transcripts under normal conditions (Supplementary Figure S10A, Figure 8F) (36,37). Therefore, RRP6 seems to function on these nucleoplasmic substrates only when DIS3 is inhibited. Such a conditional compensation of DIS3 by RRP6 was also observed in a previous study, albeit only for several PROMPT molecules, and explained by RRP6 re-localization to the nucleoplasm from the nucleolus (37). Although such a drastic change in RRP6 localization was undetected under our experimental conditions, the expression of other exosome components, including RRP6, was somewhat increased in the absence of DIS3; this may account for the activity of the RRP6-incorporated exosome complex in the nucleoplasm (Supplementary Figure S10B, Figure 1A), as well as a subtle but significant decrease in the nuclear/cytoplasmic poly(A)^+^ RNA intensity ratio often observed under experimental DIS3 KD conditions (e.g. Figure 1B and C, Supplementary Figure S3A and B, Figure 6A and B).

MPP6 simultaneously binds to MTR4 and the core with distinct regions, just as previous structural analyses demonstrate (99–102). MTR4 recruitment to the core by MPP6 is essential for its stimulating activity on both DIS3 and RRP6. Specifically, MPP6-bound MTR4 dominantly sustains DIS3-executed poly(A)^+^ substrate decay, and RRP6-recruited MTR4 cannot efficiently support this process. In experiments using the 4M channel-occluding mutant, the substrate stabilization by MPP6 KD in 4M-expressing cells was larger than that in the wild-type RRP41-expressing cells, which can mean that, to some extent, RRP6-recruited MTR4 supports the substrate path to DIS3 (Supplementary Figure S3A and B). However, it is also possible that this effect is due to the latent effect of the introduced 4M-mutation on the upper channel surface functioning in the substrate supply to RRP6, which is only manifested in the absence of MPP6. The failure of catalytically inert RRP6s to support substrate decay in the absence of MPP6, and the mitigation of DIS3*^endo-exo^^−^*-linked substrate accumulation by MPP6 KD, led us to conclude that MPP6-bound MTR4 is essential for DIS3 to efficiently access the substrates. Additionally, poly(A)^+^ substrate accumulation was more pronounced in MPP6 KD cells although the diminishment of MTR4–core complexes was greater under the RRP6 KD condition than under the MPP6 KD condition (Figure 5A–D, Figure 3A–C). This highlights the functional significance of the proper arrangement of MTR4 within the exosome complex and/or the appropriate cofactor composition of the MTR4-bound exosome, rather than the mere abundance of bound MTR4. Although our results indicate that MPP6-recruited MTR4 has a function that cannot be substituted, at least regarding the degradation of a certain subset of substrates, it is also probable that MTR4, which RRP6 recruits to the core, maintains the decay process conducted by DIS3 for other substrates, as reported in previous *in vitro* analyses (99–102).

Our genome-wide survey indicates that MPP6 conducts in the decay of a limited subset among the entire nuclear exosome substrates. When stabilized, they manifest as a robust poly(A)^+^ RNA accumulation, despite their narrow range. Nuclear poly(A)^+^ RNA accumulation is also used as a marker of mRNA transport defects. However, no obvious effect of exosome inhibition on the nuclear export of transiently expressed reporter mRNAs (Supplementary Fig. S10C) (87) nor any significant correlation between the nuclear poly(A)^+^RNA profiles in the exosome-inhibited and the mRNA nuclear export-perturbed cells was observed (Supplementary Figure S10D). Therefore, the accumulated poly(A)^+^ RNAs reflects stabilized poly(A)^+^ substrates that would normally be degraded by the exosome and is probably not the result of compromised mRNA transport in general. Indeed, several of our identified substrates co-localized with poly(A)^+^ foci when stabilized. How is it possible to achieve such a remarkable phenotype with such a limited substrate? We observed a significantly higher expression level for the exosome substrates over the non-substrates, and for the RRP6/MPP6 KD stabilized over the RRP6/MPP6 KD NOT stabilized substrates under the RRP6/DIS3 KD condition (Supplementary Figure S10E). However, considering the overwhelming variety in the non-substrate subset, it is difficult to conclude that the high abundance of the substrates alone can explain the significant poly(A)^+^ signal. Although it is currently very difficult to conclude the decision for the pronounced poly(A)^+^ accumulating phenotype, other RNA characteristics may contribute to this prominent phenotype, such as the extension of poly(A)^+^ tail length and/or the localization of diverse transcripts to a constrained space due to aggregation (66,83,147,148). These characteristics are also related to those of the substrates we have identified. Namely, the substantially higher PAXT sensitivity, which may result in the extension of their poly(A)^+^ tails, as previously demonstrated (66), and the lower extractability, which indicates their inclusion in persistent aggregates (140). Additionally, it is also possible that a number of 3' adenylated ends mapped outside the exon features that were unaddressed in this analysis may largely contribute to the poly(A)^+^ accumulating phenotype (approximately up to half of the mapped reads were within ‘exon’ features, Supplementary Figure S10F), although their sensitivity to each exosome component or their adenylation status is unknown thus far (e.g. whether canonical PAPs polyadenylate them or not). Carefully dissecting these unaddressed 3’ ends, as well as further analysis including the information about the global poly(A)^+^ tail status, which was missing from our NGS libraries, would help to clarify the cause of such a pronounced poly(A)^+^ aggregates.

Among RRP6/MPP6 KD stabilized substrates, we have identified many transcripts from the relatively short transcription units (TUs), including variant snRNAs and replication-dependent histone (RDH)-coding RNAs. Although we have not probed if this short length is a determinant of MPP6-sensitivity, several lines of evidence support the finding of a concentration of short TU transcripts among RRP6/MPP6 KD stabilized substrates. It has been reported that ARS2 achieves early transcription termination of short TUs, prompting us to assess the contribution of poly(A)^+^ ends generated via ARS2 function in forming the exosome KD-derived poly(A)^+^ aggregates (90,141). ARS2 depletion significantly restored nuclear poly(A)^+^ RNA accumulation (Supplementary Figure S10G and H). Additionally, when cells were treated with Leptomycin B (LMB), an inhibitor of the CRM1 transporter (149), the extent of nuclear poly(A)^+^ RNA accumulation in both HeLa and A549 cells deprived of MTR4 was significantly enhanced, implying that CRM1 has a function linked to the transport of these substrates to the cytoplasm under MTR4 KD conditions (Supplementary Figure S10I and J). Since it has been demonstrated that PHAX/CRM1, an adaptor and transporter complex, functions in the nuclear export of short length Pol II-transcribed transcripts (150), this result also supports that RRP6/MPP6 KD UP substrates are enriched in short transcripts. Thus, the short length of these transcripts or some features associated with the mechanism underlying their generation might confer them with increased susceptibility to MPP6.

MPP6 appears to be considerably involved in the PAXT-dependent substrate decay. However, bulk poly(A)^+^ RNAs and several individual RRP6/MPP6 KD stabilized substrates, including *vU1snRNA* and *proDNAJB4*, were not markedly stabilized by the depletion of either PAXT components (ZFC3H1 or PABPN1) (Supplementary Figure S2C and H–J). With the simultaneous knockdown of RRP45 with these factors, poly(A)^+^ addition to the substrate occurs even in the absence of ZFC3H1 and PABPN1, indicating that the lesser degree of substrate stabilization observed under ZFC3H1- or PABPN1-depleted conditions may not be due to the poor adenylation of substrates, but to the small amount of substrate present. Hence, PAXT seems not to dominate the degradation of these substrates. It is unelucidated whether it is the distinctive dependency on a specific cofactor or the significant redundancy between cofactors to recruit MTR4 that functions to decay them. In line with the poor involvement of MPP6 in the NEXT-dependent substrate decay observed in our analyses, previous structural analyses have suggested that ZCCHC8, a component of the NEXT complex, competes with MPP6-bound MTR4 for binding to the exosome core (103). Such an MPP6- irrelevant function of NEXT-exosome has been also proposed by a very recent study showing that the exclusively NEXT-dependent trimming of the 3’ ends of snoRNAs can somehow progress in the absence of RRP40, a component of the exosome core that provides the main surface for binding to MPP6 (99,101). Although the contribution of core-independent exonucleases, namely PARN and RRP6, to this trimming process was suggested in that study, some residual NEXT-exosome activity may also be involved, since RRP40 depletion seems to have only a minor effect on the abundance of other core components and cofactors in their experiments. We only dissected the adenylated substrates in this study, hence we could not evaluate the precise impact of RRP6/MPP6 KD on NEXT-exosome function. Again, scrutiny over a wider range of substrates to elucidate their sensitivities to various cofactors including PAXT and NEXT, as well as their adenylated/non-adenylated states should allow us to further understand how distinct exosome complexes functions *in vivo*.

## DATA AVAILABILITY

RNA-seq data reported in this study have been deposited with GEO under accession number GSE184274 and with JGA under accession number JGAS000294.

## Supplementary Material

gkac559_Supplemental_Files

## Notes

Present address: Naoko Fujiwara, Graduate School of Frontier Biosciences, Osaka University, Suita, Osaka 565-0871, Japan.

## References

[1] Birney E. , Stamatoyannopoulos J.A., Dutta A., Guigó R., Gingeras T.R., Margulies E.H., Weng Z., Snyder M., Dermitzakis E.T., Thurman R.E. et al. Identification and analysis of functional elements in 1% of the human genome by the ENCODE pilot project. Nature. 2007; 447:799–816. 17571346 10.1038/nature05874 PMC2212820

[2] Djebali S. , Davis C.A., Merkel A., Dobin A., Lassmann T., Mortazavi A., Tanzer A., Lagarde J., Lin W., Schlesinger F. et al. Landscape of transcription in human cells. Nature. 2012; 489:101–108. 22955620 10.1038/nature11233 PMC3684276

[3] Nair L. , Chung H., Basu U. Regulation of long non-coding RNAs and genome dynamics by the RNA surveillance machinery. Nat. Rev. Mol. Cell Biol. 2020; 21:123–136. 32020081 10.1038/s41580-019-0209-0 PMC7107043

[4] Ogami K. , Chen Y., Manley J.L. RNA surveillance by the nuclear RNA exosome: mechanisms and significance. Non-coding RNA. 2018; 4:8. 29629374 10.3390/ncrna4010008 PMC5886371

[5] Łabno A. , Tomecki R., Dziembowski A. Cytoplasmic RNA decay pathways - enzymes and mechanisms. Biochim. Biophys. Acta - Mol. Cell Res. 2016; 1863:3125–3147. 10.1016/j.bbamcr.2016.09.023 27713097

[6] Kilchert C. , Wittmann S., Vasiljeva L. The regulation and functions of the nuclear RNA exosome complex. Nat. Rev. Mol. Cell Biol. 2016; 17:227–239. 26726035 10.1038/nrm.2015.15

[7] Doma M.K. , Parker R. RNA quality control in eukaryotes. Cell. 2007; 131:660–668. 18022361 10.1016/j.cell.2007.10.041

[8] Zinder J.C. , Lima C.D. Targeting RNA for processing or destruction by the eukaryotic RNA exosome and its cofactors. Genes Dev. 2017; 31:88–100. 28202538 10.1101/gad.294769.116 PMC5322736

[9] Lorentzen E. , Walter P., Fribourg S., Evguenieva-Hackenberg E., Klug G., Conti E. The archaeal exosome core is a hexameric ring structure with three catalytic subunits. Nat. Struct. Mol. Biol. 2005; 12:575–581. 15951817 10.1038/nsmb952

[10] Dziembowski A. , Lorentzen E., Conti E., Séraphin B. A single subunit, dis3, is essentially responsible for yeast exosome core activity. Nat. Struct. Mol. Biol. 2007; 14:15–22. 17173052 10.1038/nsmb1184

[11] Liu Q. , Greimann J.C., Lima C.D. Reconstitution, activities, and structure of the eukaryotic RNA exosome. Cell. 2006; 127:1223–1237. 17174896 10.1016/j.cell.2006.10.037

[12] Lorentzen E. , Basquin J., Conti E. Structural organization of the RNA-degrading exosome. Curr. Opin. Struct. Biol. 2008; 18:709–713. 18955140 10.1016/j.sbi.2008.10.004

[13] Wang H.-W. , Wang J., Ding F., Callahan K., Bratkowski M.A., Butler J.S., Nogales E., Ke A. Architecture of the yeast rrp44 exosome complex suggests routes of RNA recruitment for 3’ end processing. Proc. Natl. Acad. Sci. U.S.A. 2007; 104:16844–16849. 17942686 10.1073/pnas.0705526104 PMC2040474

[14] Lebreton A. , Tomecki R., Dziembowski A., Séraphin B. Endonucleolytic RNA cleavage by a eukaryotic exosome. Nature. 2008; 456:993–996. 19060886 10.1038/nature07480

[15] Schaeffer D. , Tsanova B., Barbas A., Reis F.P., Dastidar E.G., Sanchez-Rotunno M., Arraiano C.M., van Hoof A. The exosome contains domains with specific endoribonuclease, exoribonuclease and cytoplasmic mRNA decay activities. Nat. Struct. Mol. Biol. 2009; 16:56–62. 19060898 10.1038/nsmb.1528 PMC2615074

[16] Schneider C. , Leung E., Brown J., Tollervey D. The N-terminal PIN domain of the exosome subunit rrp44 harbors endonuclease activity and tethers rrp44 to the yeast core exosome. Nucleic Acids Res. 2009; 37:1127–1140. 19129231 10.1093/nar/gkn1020 PMC2651783

[17] Bonneau F. , Basquin J., Ebert J., Lorentzen E., Conti E. The yeast exosome functions as a macromolecular cage to channel RNA substrates for degradation. Cell. 2009; 139:547–559. 19879841 10.1016/j.cell.2009.08.042

[18] Schaeffer D. , Reis F.P., Johnson S.J., Arraiano C.M., van Hoof A. The CR3 motif of rrp44p is important for interaction with the core exosome and exosome function. Nucleic Acids Res. 2012; 40:9298–9307. 22833611 10.1093/nar/gks693 PMC3467083

[19] Tomecki R. , Kristiansen M.S., Lykke-Andersen S., Chlebowski A., Larsen K.M., Szczesny R.J., Drazkowska K., Pastula A., Andersen J.S., Stepien P.P. et al. The human core exosome interacts with differentially localized processive RNases: hDIS3 and hDIS3L. EMBO J. 2010; 29:2342–2357. 20531386 10.1038/emboj.2010.121 PMC2910271

[20] Staals R.H.J. , Bronkhorst A.W., Schilders G., Slomovic S., Schuster G., Heck A.J.R., Raijmakers R., Pruijn G.J.M. Dis3-like 1: a novel exoribonuclease associated with the human exosome. EMBO J. 2010; 29:2358–2367. 20531389 10.1038/emboj.2010.122 PMC2910272

[21] Lorentzen E. , Basquin J., Tomecki R., Dziembowski A., Conti E. Structure of the active subunit of the yeast exosome core, rrp44: diverse modes of substrate recruitment in the RNase II nuclease family. Mol. Cell. 2008; 29:717–728. 18374646 10.1016/j.molcel.2008.02.018

[22] Chang H.-M. , Triboulet R., Thornton J.E., Gregory R.I. A role for the perlman syndrome exonuclease dis3l2 in the Lin28–let-7 pathway. Nature. 2013; 497:244–248. 23594738 10.1038/nature12119 PMC3651781

[23] Malecki M. , Viegas S.C., Carneiro T., Golik P., Dressaire C., Ferreira M.G., Arraiano C.M. The exoribonuclease dis3l2 defines a novel eukaryotic RNA degradation pathway. EMBO J. 2013; 32:1842–1854. 23503588 10.1038/emboj.2013.63 PMC3981172

[24] Lubas M. , Damgaard C.K., Tomecki R., Cysewski D., Jensen T.H., Dziembowski A. Exonuclease hDIS3L2 specifies an exosome-independent 3′-5′ degradation pathway of human cytoplasmic mRNA. EMBO J. 2013; 32:1855–1868. 23756462 10.1038/emboj.2013.135 PMC3981170

[25] Briggs M.W. , Burkard K.T.D., Butler J.S. Rrp6p, the yeast homologue of the human PM-Scl 100-kDa autoantigen, is essential for efficient 5.8 s rRNA 3′ end formation. J. Biol. Chem. 1998; 273:13255–13263. 9582370 10.1074/jbc.273.21.13255

[26] Allmang C. , Petfalski E., Podtelejnikov A., Mann M., Tollervey D., Mitchell P. The yeast exosome and human PM-Scl are related complexes of 3’ right-arrow 5’ exonucleases. Genes Dev. 1999; 13:2148–2158. 10465791 10.1101/gad.13.16.2148 PMC316947

[27] Januszyk K. , Liu Q., Lima C.D. Activities of human RRP6 and structure of the human RRP6 catalytic domain. RNA. 2011; 17:1566–1577. 21705430 10.1261/rna.2763111 PMC3153979

[28] Makino D.L. , Baumgärtner M., Conti E. Crystal structure of an RNA-bound 11-subunit eukaryotic exosome complex. Nature. 2013; 495:70–75. 23376952 10.1038/nature11870

[29] Wasmuth E.V , Januszyk K., Lima C.D. Structure of an Rrp6–RNA exosome complex bound to poly(A) RNA. Nature. 2014; 511:435–439. 25043052 10.1038/nature13406 PMC4310248

[30] Makino D.L. , Schuch B., Stegmann E., Baumgärtner M., Basquin C., Conti E. RNA degradation paths in a 12-subunit nuclear exosome complex. Nature. 2015; 524:54–58. 26222026 10.1038/nature14865

[31] Zinder J.C. , Wasmuth E.V., Lima C.D. Nuclear RNA exosome at 3.1 Å reveals substrate specificities, RNA paths, and allosteric inhibition of rrp44/dis3. Mol. Cell. 2016; 64:734–745. 27818140 10.1016/j.molcel.2016.09.038 PMC5115963

[32] Schneider C. , Kudla G., Wlotzka W., Tuck A., Tollervey D. Transcriptome-wide analysis of exosome targets. Mol. Cell. 2012; 48:422–433. 23000172 10.1016/j.molcel.2012.08.013 PMC3526797

[33] Schilders G. MPP6 is an exosome-associated RNA-binding protein involved in 5.8S rRNA maturation. Nucleic Acids Res. 2005; 33:6795–6804. 16396833 10.1093/nar/gki982 PMC1310903

[34] Sloan K.E. , Mattijssen S., Lebaron S., Tollervey D., Pruijn G.J.M., Watkins N.J. Both endonucleolytic and exonucleolytic cleavage mediate ITS1 removal during human ribosomal RNA processing. J. Cell Biol. 2013; 200:577–588. 23439679 10.1083/jcb.201207131 PMC3587827

[35] Kobyłecki K. , Drążkowska K., Kuliński T.M., Dziembowski A., Tomecki R. Elimination of 01/A′–A0 pre-rRNA processing by-product in human cells involves cooperative action of two nuclear exosome-associated nucleases: RRP6 and DIS3. RNA. 2018; 24:1677–1692. 30266864 10.1261/rna.066589.118 PMC6239190

[36] Davidson L. , Francis L., Cordiner R.A., Eaton J.D., Estell C., Macias S., Cáceres J.F., West S. Rapid depletion of DIS3, EXOSC10, or XRN2 reveals the immediate impact of exoribonucleolysis on nuclear RNA metabolism and transcriptional control. Cell Rep. 2019; 26:2779–2791. 30840897 10.1016/j.celrep.2019.02.012 PMC6403362

[37] Szczepińska T. , Kalisiak K., Tomecki R., Labno A., Borowski L.S., Kulinski T.M., Adamska D., Kosinska J., Dziembowski A. DIS3 shapes the RNA polymerase II transcriptome in humans by degrading a variety of unwanted transcripts. Genome Res. 2015; 25:1622–1633. 26294688 10.1101/gr.189597.115 PMC4617959

[38] Weick E.-M. , Lima C.D. RNA helicases are hubs that orchestrate exosome-dependent 3’-5’ decay. Curr. Opin. Struct. Biol. 2021; 67:86–94. 33147539 10.1016/j.sbi.2020.09.010 PMC8087718

[39] Kadaba S. , Krueger A., Trice T., Krecic A.M., Hinnebusch A.G., Anderson J. Nuclear surveillance and degradation of hypomodified initiator tRNAMet in s. cerevisiae. Genes Dev. 2004; 18:1227–1240. 15145828 10.1101/gad.1183804 PMC420349

[40] LaCava J. , Houseley J., Saveanu C., Petfalski E., Thompson E., Jacquier A., Tollervey D. RNA degradation by the exosome is promoted by a nuclear polyadenylation complex. Cell. 2005; 121:713–724. 15935758 10.1016/j.cell.2005.04.029

[41] Wyers F. , Rougemaille M., Badis G., Rousselle J.-C., Dufour M.-E., Boulay J., Régnault B., Devaux F., Namane A., Séraphin B. et al. Cryptic pol II transcripts are degraded by a nuclear quality control pathway involving a new poly(a) polymerase. Cell. 2005; 121:725–737. 15935759 10.1016/j.cell.2005.04.030

[42] Vaňáčová Š. , Wolf J., Martin G., Blank D., Dettwiler S., Friedlein A., Langen H., Keith G., Keller W. A new yeast poly(a) polymerase complex involved in RNA quality control. PLoS Biol. 2005; 3:e189. 15828860 10.1371/journal.pbio.0030189 PMC1079787

[43] Delan-Forino C. , Spanos C., Rappsilber J., Tollervey D. Substrate specificity of the TRAMP nuclear surveillance complexes. Nat. Commun. 2020; 11:3122. 32561742 10.1038/s41467-020-16965-4 PMC7305330

[44] Vasiljeva L. , Buratowski S. Nrd1 interacts with the nuclear exosome for 3′ processing of RNA polymerase II transcripts. Mol. Cell. 2006; 21:239–248. 16427013 10.1016/j.molcel.2005.11.028

[45] Tudek A. , Porrua O., Kabzinski T., Lidschreiber M., Kubicek K., Fortova A., Lacroute F., Vanacova S., Cramer P., Stefl R. et al. Molecular basis for coordinating transcription termination with noncoding RNA degradation. Mol. Cell. 2014; 55:467–481. 25066235 10.1016/j.molcel.2014.05.031 PMC4186968

[46] Lubas M. , Christensen M.S., Kristiansen M.S., Domanski M., Falkenby L.G., Lykke-Andersen S., Andersen J.S., Dziembowski A., Jensen T.H. Interaction profiling identifies the human nuclear exosome targeting complex. Mol. Cell. 2011; 43:624–637. 21855801 10.1016/j.molcel.2011.06.028

[47] Meola N. , Domanski M., Karadoulama E., Chen Y., Gentil C., Pultz D., Vitting-Seerup K., Lykke-Andersen S., Andersen J.S., Sandelin A. et al. Identification of a nuclear exosome decay pathway for processed transcripts. Mol. Cell. 2016; 64:520–533. 27871484 10.1016/j.molcel.2016.09.025

[48] Shcherbik N. , Wang M., Lapik Y.R., Srivastava L., Pestov D.G. Polyadenylation and degradation of incomplete RNA polymerase i transcripts in mammalian cells. EMBO Rep. 2010; 11:106–111. 20062005 10.1038/embor.2009.271 PMC2828747

[49] Sudo H. , Nozaki A., Uno H., Ishida Y., Nagahama M. Interaction properties of human TRAMP-like proteins and their role in pre-rRNA 5′ETS turnover. FEBS Lett. 2016; 590:2963–2972. 27434818 10.1002/1873-3468.12314

[50] Tseng C.-K. , Wang H.-F., Burns A.M., Schroeder M.R., Gaspari M., Baumann P. Human telomerase RNA processing and quality control. Cell Rep. 2015; 13:2232–2243. 26628367 10.1016/j.celrep.2015.10.075

[51] Nguyen D. , Grenier St-Sauveur V., Bergeron D., Dupuis-Sandoval F., Scott M.S., Bachand F. A polyadenylation-dependent 3′ end maturation pathway is required for the synthesis of the human telomerase RNA. Cell Rep. 2015; 13:2244–2257. 26628368 10.1016/j.celrep.2015.11.003

[52] Shukla S. , Schmidt J.C., Goldfarb K.C., Cech T.R., Parker R. Inhibition of telomerase RNA decay rescues telomerase deficiency caused by dyskerin or PARN defects. Nat. Struct. Mol. Biol. 2016; 23:286–292. 26950371 10.1038/nsmb.3184 PMC4830462

[53] Rammelt C. , Bilen B., Zavolan M., Keller W. PAPD5, a noncanonical poly(A) polymerase with an unusual RNA-binding motif. RNA. 2011; 17:1737–1746. 21788334 10.1261/rna.2787011 PMC3162338

[54] Berndt H. , Harnisch C., Rammelt C., Stohr N., Zirkel A., Dohm J.C., Himmelbauer H., Tavanez J.-P., Huttelmaier S., Wahle E. Maturation of mammalian H/ACA box snoRNAs: PAPD5-dependent adenylation and PARN-dependent trimming. RNA. 2012; 18:958–972. 22442037 10.1261/rna.032292.112 PMC3334704

[55] Schmidt M. , Norbury C.J. Polyadenylation and beyond: emerging roles for noncanonical poly(A) polymerases. Wiley Interdiscip. Rev. RNA. 2010; 1:142–151. 21956911 10.1002/wrna.16

[56] Topalian S.L. , Kaneko S., Gonzales M.I., Bond G.L., Ward Y., Manley J.L. Identification and functional characterization of neo-poly(a) polymerase, an RNA processing enzyme overexpressed in human tumors. Mol. Cell. Biol. 2001; 21:5614–5623. 11463842 10.1128/MCB.21.16.5614-5623.2001 PMC87282

[57] Shi Y. , Manley J.L. The end of the message: multiple protein–RNA interactions define the mRNA polyadenylation site. Genes Dev. 2015; 29:889–897. 25934501 10.1101/gad.261974.115 PMC4421977

[58] Lim J. , Kim D., Lee Y., Ha M., Lee M., Yeo J., Chang H., Song J., Ahn K., Kim V.N. Mixed tailing by TENT4A and TENT4B shields mRNA from rapid deadenylation. Science. 2018; 361:701–704. 30026317 10.1126/science.aam5794

[59] Tseng C.-K. , Wang H.-F., Schroeder M.R., Baumann P. The H/ACA complex disrupts triplex in hTR precursor to permit processing by RRP6 and PARN. Nat. Commun. 2018; 9:5430. 30575725 10.1038/s41467-018-07822-6 PMC6303318

[60] Gable D.L. , Gaysinskaya V., Atik C.C., Talbot C.C., Kang B., Stanley S.E., Pugh E.W., Amat-Codina N., Schenk K.M., Arcasoy M.O. et al. ZCCHC8, the nuclear exosome targeting component, is mutated in familial pulmonary fibrosis and is required for telomerase RNA maturation. Genes Dev. 2019; 33:1381–1396. 31488579 10.1101/gad.326785.119 PMC6771387

[61] Lubas M. , Andersen P.R., Schein A., Dziembowski A., Kudla G., Jensen T.H. The human nuclear exosome targeting complex is loaded onto newly synthesized RNA to direct early ribonucleolysis. Cell Rep. 2015; 10:178–192. 25578728 10.1016/j.celrep.2014.12.026

[62] Hrossova D. , Sikorsky T., Potesil D., Bartosovic M., Pasulka J., Zdrahal Z., Stefl R., Vanacova S. RBM7 subunit of the NEXT complex binds U-rich sequences and targets 3′-end extended forms of snRNAs. Nucleic Acids Res. 2015; 43:4236–4248. 25852104 10.1093/nar/gkv240 PMC4417160

[63] Falk S. , Finogenova K., Melko M., Benda C., Lykke-Andersen S., Jensen T.H., Conti E. Structure of the RBM7–ZCCHC8 core of the NEXT complex reveals connections to splicing factors. Nat. Commun. 2016; 7:13573. 27905398 10.1038/ncomms13573 PMC5146272

[64] Banerjee A. , Apponi L.H., Pavlath G.K., Corbett A.H. PABPN1: molecular function and muscle disease. FEBS J. 2013; 280:4230–4250. 23601051 10.1111/febs.12294 PMC3786098

[65] Beaulieu Y.B. , Kleinman C.L., Landry-Voyer A.-M., Majewski J., Bachand F. Polyadenylation-Dependent control of long noncoding RNA expression by the poly(a)-binding protein nuclear 1. PLoS Genet. 2012; 8:e1003078. 23166521 10.1371/journal.pgen.1003078 PMC3499365

[66] Bresson S.M. , Conrad N.K. The human nuclear poly(a)-binding protein promotes RNA hyperadenylation and decay. PLoS Genet. 2013; 9:e1003893. 24146636 10.1371/journal.pgen.1003893 PMC3798265

[67] Bresson S.M. , Hunter O.V., Hunter A.C., Conrad N.K. Canonical poly(a) polymerase activity promotes the decay of a wide variety of mammalian nuclear RNAs. PLoS Genet. 2015; 11:e1005610. 26484760 10.1371/journal.pgen.1005610 PMC4618350

[68] Muniz L. , Davidson L., West S. Poly(A) polymerase and the nuclear poly(a) binding protein, PABPN1, coordinate the splicing and degradation of a subset of human Pre-mRNAs. Mol. Cell. Biol. 2015; 35:2218–2230. 25896913 10.1128/MCB.00123-15 PMC4456446

[69] Yamanaka S. , Yamashita A., Harigaya Y., Iwata R., Yamamoto M. Importance of polyadenylation in the selective elimination of meiotic mRNAs in growing s. pombe cells. EMBO J. 2010; 29:2173–2181. 20512112 10.1038/emboj.2010.108 PMC2905246

[70] St-André O. , Lemieux C., Perreault A., Lackner D.H., Bähler J., Bachand F. Negative regulation of meiotic gene expression by the nuclear Poly(a)-binding protein in fission yeast. J. Biol. Chem. 2010; 285:27859–27868. 20622014 10.1074/jbc.M110.150748 PMC2934653

[71] Chen H.-M. , Futcher B., Leatherwood J. The fission yeast RNA binding protein mmi1 regulates meiotic genes by controlling intron specific splicing and polyadenylation coupled RNA turnover. PLoS One. 2011; 6:e26804. 22046364 10.1371/journal.pone.0026804 PMC3203177

[72] Zhou Y. , Zhu J., Schermann G., Ohle C., Bendrin K., Sugioka-Sugiyama R., Sugiyama T., Fischer T. The fission yeast MTREC complex targets CUTs and unspliced pre-mRNAs to the nuclear exosome. Nat. Commun. 2015; 6:7050. 25989903 10.1038/ncomms8050 PMC4455066

[73] Kaida D. , Berg M.G., Younis I., Kasim M., Singh L.N., Wan L., Dreyfuss G. U1 snRNP protects pre-mRNAs from premature cleavage and polyadenylation. Nature. 2010; 468:664–668. 20881964 10.1038/nature09479 PMC2996489

[74] Chiu A.C. , Suzuki H.I., Wu X., Mahat D.B., Kriz A.J., Sharp P.A. Transcriptional pause sites delineate stable nucleosome-associated premature polyadenylation suppressed by U1 snRNP. Mol. Cell. 2018; 69:648–663. 29398447 10.1016/j.molcel.2018.01.006 PMC6175280

[75] So B.R. , Di C., Cai Z., Venters C.C., Guo J., Oh J.-M., Arai C., Dreyfuss G. A complex of U1 snRNP with cleavage and polyadenylation factors controls telescripting, regulating mRNA transcription in human cells. Mol. Cell. 2019; 76:590–599. 31522989 10.1016/j.molcel.2019.08.007 PMC6874754

[76] Kamieniarz-Gdula K. , Gdula M.R., Panser K., Nojima T., Monks J., Wiśniewski J.R., Riepsaame J., Brockdorff N., Pauli A., Proudfoot N.J. Selective roles of vertebrate PCF11 in premature and full-length transcript termination. Mol. Cell. 2019; 74:158–172. 30819644 10.1016/j.molcel.2019.01.027 PMC6458999

[77] Almada A.E. , Wu X., Kriz A.J., Burge C.B., Sharp P.A. Promoter directionality is controlled by U1 snRNP and polyadenylation signals. Nature. 2013; 499:360–363. 23792564 10.1038/nature12349 PMC3720719

[78] Ntini E. , Järvelin A.I., Bornholdt J., Chen Y., Boyd M., Jørgensen M., Andersson R., Hoof I., Schein A., Andersen P.R. et al. Polyadenylation site–induced decay of upstream transcripts enforces promoter directionality. Nat. Struct. Mol. Biol. 2013; 20:923–928. 23851456 10.1038/nsmb.2640

[79] Andersen P.R. , Domanski M., Kristiansen M.S., Storvall H., Ntini E., Verheggen C., Schein A., Bunkenborg J., Poser I., Hallais M. et al. The human cap-binding complex is functionally connected to the nuclear RNA exosome. Nat. Struct. Mol. Biol. 2013; 20:1367–1376. 24270879 10.1038/nsmb.2703 PMC3923317

[80] Lardelli R.M. , Lykke-Andersen J. Competition between maturation and degradation drives human snRNA 3′ end quality control. Genes Dev. 2020; 34:989–1001. 32499401 10.1101/gad.336891.120 PMC7328512

[81] Kawamoto T. , Yoshimoto R., Taniguchi I., Kitabatake M., Ohno M. 2021) ISG20 and nuclear exosome promote destabilization of nascent transcripts for spliceosomal u snRNAs and U1 variants. Genes Cells. 26:18–30. 33147372 10.1111/gtc.12817

[82] Ogami K. , Richard P., Chen Y., Hoque M., Li W., Moresco J.J., Yates J.R., Tian B., Manley J.L. An mtr4/zfc3h1 complex facilitates turnover of unstable nuclear RNAs to prevent their cytoplasmic transport and global translational repression. Genes Dev. 2017; 31:1257–1271. 28733371 10.1101/gad.302604.117 PMC5558927

[83] Silla T. , Karadoulama E., Mąkosa D., Lubas M., Jensen T.H. The RNA exosome adaptor ZFC3H1 functionally competes with nuclear export activity to retain target transcripts. Cell Rep. 2018; 23:2199–2210. 29768216 10.1016/j.celrep.2018.04.061 PMC5972229

[84] Silla T. , Schmid M., Dou Y., Garland W., Milek M., Imami K., Johnsen D., Polak P., Andersen J.S., Selbach M. et al. The human ZC3H3 and RBM26/27 proteins are critical for PAXT-mediated nuclear RNA decay. Nucleic Acids Res. 2020; 48:2518–2530. 31950173 10.1093/nar/gkz1238 PMC7049725

[85] Wu G. , Schmid M., Rib L., Polak P., Meola N., Sandelin A., Jensen T.H. A two-layered targeting mechanism underlies nuclear RNA sorting by the human exosome. Cell Rep. 2020; 30:2387–2401. 32075771 10.1016/j.celrep.2020.01.068

[86] Fan J. , Kuai B., Wu G., Wu X., Chi B., Wang L., Wang K., Shi Z., Zhang H., Chen S. et al. Exosome cofactor hMTR4 competes with export adaptor ALYREF to ensure balanced nuclear RNA pools for degradation and export. EMBO J. 2017; 36:2870–2886. 28801509 10.15252/embj.201696139 PMC5623860

[87] Fan J. , Kuai B., Wang K., Wang L., Wang Y., Wu X., Chi B., Li G., Cheng H. mRNAs are sorted for export or degradation before passing through nuclear speckles. Nucleic Acids Res. 2018; 46:8404–8416. 30032211 10.1093/nar/gky650 PMC6144872

[88] Weir J.R. , Bonneau F., Hentschel J., Conti E. Structural analysis reveals the characteristic features of mtr4, a DExH helicase involved in nuclear RNA processing and surveillance. Proc. Natl. Acad. Sci. U.S.A. 2010; 107:12139–12144. 20566885 10.1073/pnas.1004953107 PMC2901443

[89] Jackson R.N. , Klauer A.A., Hintze B.J., Robinson H., van Hoof A., Johnson S.J. The crystal structure of mtr4 reveals a novel arch domain required for rRNA processing. EMBO J. 2010; 29:2205–2216. 20512111 10.1038/emboj.2010.107 PMC2905245

[90] Hallais M. , Pontvianne F., Andersen P.R., Clerici M., Lener D., Benbahouche N.E.H., Gostan T., Vandermoere F., Robert M.-C., Cusack S. et al. CBC–ARS2 stimulates 3′-end maturation of multiple RNA families and favors cap-proximal processing. Nat. Struct. Mol. Biol. 2013; 20:1358–1366. 24270878 10.1038/nsmb.2720

[91] Giacometti S. , Benbahouche N.E.H., Domanski M., Robert M.-C., Meola N., Lubas M., Bukenborg J., Andersen J.S., Schulze W.M., Verheggen C. et al. Mutually exclusive CBC-containing complexes contribute to RNA fate. Cell Rep. 2017; 18:2635–2650. 28297668 10.1016/j.celrep.2017.02.046 PMC5368414

[92] Schulze W.M. , Stein F., Rettel M., Nanao M., Cusack S. Structural analysis of human ARS2 as a platform for co-transcriptional RNA sorting. Nat. Commun. 2018; 9:1701. 29703953 10.1038/s41467-018-04142-7 PMC5923425

[93] Lykke-Andersen S. , Rouvière J.O., Jensen T.H. ARS2/SRRT: at the nexus of RNA polymerase II transcription, transcript maturation and quality control. Biochem. Soc. Trans. 2021; 49:1325–1336. 34060620 10.1042/BST20201008

[94] Wang J. , Chen J., Wu G., Zhang H., Du X., Chen S., Zhang L., Wang K., Fan J., Gao S. et al. NRDE2 negatively regulates exosome functions by inhibiting MTR4 recruitment and exosome interaction. Genes Dev. 2019; 33:536–549. 30842217 10.1101/gad.322602.118 PMC6499326

[95] Thoms M. , Thomson E., Baßler J., Gnädig M., Griesel S., Hurt E. The exosome is recruited to RNA substrates through specific adaptor proteins. Cell. 2015; 162:1029–1038. 26317469 10.1016/j.cell.2015.07.060

[96] Lingaraju M. , Johnsen D., Schlundt A., Langer L.M., Basquin J., Sattler M., Heick Jensen T., Falk S., Conti E. The MTR4 helicase recruits nuclear adaptors of the human RNA exosome using distinct arch-interacting motifs. Nat. Commun. 2019; 10:3393. 31358741 10.1038/s41467-019-11339-x PMC6662825

[97] Stead J.A. , Costello J.L., Livingstone M.J., Mitchell P. The PMC2NT domain of the catalytic exosome subunit rrp6p provides the interface for binding with its cofactor rrp47p, a nucleic acid-binding protein. Nucleic Acids Res. 2007; 35:5556–5567. 17704127 10.1093/nar/gkm614 PMC2018643

[98] Schuch B. , Feigenbutz M., Makino D.L., Falk S., Basquin C., Mitchell P., Conti E. The exosome-binding factors rrp6 and rrp47 form a composite surface for recruiting the mtr4 helicase. EMBO J. 2014; 33:2829–2846. 25319414 10.15252/embj.201488757 PMC4282559

[99] Falk S. , Bonneau F., Ebert J., Kögel A., Conti E. Mpp6 incorporation in the nuclear exosome contributes to RNA channeling through the mtr4 helicase. Cell Rep. 2017; 20:2279–2286. 28877463 10.1016/j.celrep.2017.08.033 PMC5603729

[100] Wasmuth E.V. , Zinder J.C., Zattas D., Das M., Lima C.D. Structure and reconstitution of yeast Mpp6-nuclear exosome complexes reveals that mpp6 stimulates RNA decay and recruits the mtr4 helicase. Elife. 2017; 6:e29062. 28742025 10.7554/eLife.29062 PMC5553935

[101] Weick E.-M. , Puno M.R., Januszyk K., Zinder J.C., DiMattia M.A., Lima C.D. Helicase-dependent RNA decay illuminated by a Cryo-EM structure of a human nuclear RNA exosome-mtr4 complex. Cell. 2018; 173:1663–1677. 29906447 10.1016/j.cell.2018.05.041 PMC6124691

[102] Gerlach P. , Schuller J.M., Bonneau F., Basquin J., Reichelt P., Falk S., Conti E. Distinct and evolutionary conserved structural features of the human nuclear exosome complex. Elife. 2018; 7:e38686. 30047866 10.7554/eLife.38686 PMC6072439

[103] Puno M.R. , Lima C.D. Structural basis for MTR4–ZCCHC8 interactions that stimulate the MTR4 helicase in the nuclear exosome-targeting complex. Proc. Natl. Acad. Sci. U.S.A. 2018; 115:E5506–E5515. 29844170 10.1073/pnas.1803530115 PMC6004480

[104] Milligan L. , Decourty L., Saveanu C., Rappsilber J., Ceulemans H., Jacquier A., Tollervey D. A yeast exosome cofactor, mpp6, functions in RNA surveillance and in the degradation of noncoding RNA transcripts. Mol. Cell. Biol. 2008; 28:5446–5457. 18591258 10.1128/MCB.00463-08 PMC2519741

[105] Feigenbutz M. , Jones R., Besong T.M.D., Harding S.E., Mitchell P. Assembly of the yeast exoribonuclease rrp6 with its associated cofactor rrp47 occurs in the nucleus and is critical for the controlled expression of rrp47. J. Biol. Chem. 2013; 288:15959–15970. 23580640 10.1074/jbc.M112.445759 PMC3668751

[106] Feigenbutz M. , Garland W., Turner M., Mitchell P. The exosome cofactor rrp47 is critical for the stability and normal expression of its associated exoribonuclease rrp6 in saccharomyces cerevisiae. PLoS One. 2013; 8:e80752. 24224060 10.1371/journal.pone.0080752 PMC3818262

[107] Kim K. , Heo D., Kim I., Suh J.-Y., Kim M. Exosome cofactors connect transcription termination to RNA processing by guiding terminated transcripts to the appropriate exonuclease within the nuclear exosome. J. Biol. Chem. 2016; 291:13229–13242. 27076633 10.1074/jbc.M116.715771 PMC4933236

[108] Fujiwara N. , Yoshikawa M., Yamazaki T., Kambe T., Nagao M., Masuda S. A screening method tuned for mRNA processing factors in human cells by evaluation of the luciferase reporter activity and the subcellular distribution of bulk poly(a) + RNA. Biosci. Biotechnol. Biochem. 2010; 74:1512–1516. 20622428 10.1271/bbb.100363

[109] Valencia P. , Dias A.P., Reed R. Splicing promotes rapid and efficient mRNA export in mammalian cells. Proc. Natl. Acad. Sci. U.S.A. 2008; 105:3386–3391. 18287003 10.1073/pnas.0800250105 PMC2265164

[110] McQuin C. , Goodman A., Chernyshev V., Kamentsky L., Cimini B.A., Karhohs K.W., Doan M., Ding L., Rafelski S.M., Thirstrup D. et al. CellProfiler 3.0: Next-generation image processing for biology. PLoS Biol. 2018; 16:e2005970. 29969450 10.1371/journal.pbio.2005970 PMC6029841

[111] Dao D. , Fraser A.N., Hung J., Ljosa V., Singh S., Carpenter A.E. CellProfiler analyst: interactive data exploration, analysis and classification of large biological image sets. Bioinformatics. 2016; 32:3210–3212. 27354701 10.1093/bioinformatics/btw390 PMC5048071

[112] Folco E.G. , Lei H., Hsu J.L., Reed R. Small-scale nuclear extracts for functional assays of gene-expression machineries. J. Vis. Exp. 2012; 64:e4140. 10.3791/4140 PMC3471294 22782264

[113] Masuda S. , Das R., Cheng H., Hurt E., Dorman N., Reed R. Recruitment of the human TREX complex to mRNA during splicing. Genes Dev. 2005; 19:1512–1517. 15998806 10.1101/gad.1302205 PMC1172058

[114] Jones D.T. Protein secondary structure prediction based on position-specific scoring matrices. J. Mol. Biol. 1999; 292:195–202. 10493868 10.1006/jmbi.1999.3091

[115] Corpet F. Multiple sequence alignment with hierarchical clustering. Nucleic Acids Res. 1988; 16:10881–10890. 2849754 10.1093/nar/16.22.10881 PMC338945

[116] Dobin A. , Davis C.A., Schlesinger F., Drenkow J., Zaleski C., Jha S., Batut P., Chaisson M., Gingeras T.R. STAR: ultrafast universal RNA-seq aligner. Bioinformatics. 2013; 29:15–21. 23104886 10.1093/bioinformatics/bts635 PMC3530905

[117] Danecek P. , Bonfield J.K., Liddle J., Marshall J., Ohan V., Pollard M.O., Whitwham A., Keane T., McCarthy S.A., Davies R.M. et al. Twelve years of SAMtools and BCFtools. Gigascience. 2021; 10:giab008. 33590861 10.1093/gigascience/giab008 PMC7931819

[118] Anders S. , Pyl P.T., Huber W. HTSeq–a python framework to work with high-throughput sequencing data. Bioinformatics. 2015; 31:166–169. 25260700 10.1093/bioinformatics/btu638 PMC4287950

[119] Love M.I. , Huber W., Anders S. Moderated estimation of fold change and dispersion for RNA-seq data with DESeq2. Genome Biol. 2014; 15:550. 25516281 10.1186/s13059-014-0550-8 PMC4302049

[120] Wickham H. ggplot2: Elegant Graphics for Data Analysis. 2016; New York Springer-Verlag.

[121] Kurtenbach S. , Harbour J.W. SparK: A Publication-quality NGS Visualization Tool. 2019;

[122] Ramírez F. , Ryan D.P., Grüning B., Bhardwaj V., Kilpert F., Richter A.S., Heyne S., Dündar F., Manke T. deepTools2: a next generation web server for deep-sequencing data analysis. Nucleic Acids Res. 2016; 44:W160–W165. 27079975 10.1093/nar/gkw257 PMC4987876

[123] Yu G. , Wang L.G., He Q.Y. ChIP seeker: an R/Bioconductor package for ChIP peak annotation, comparison and visualization. Bioinformatics. 2015; 31:2382–2383. 25765347 10.1093/bioinformatics/btv145

[124] Yamazaki T. , Souquere S., Chujo T., Kobelke S., Chong Y.S., Fox A.H., Bond C.S., Nakagawa S., Pierron G., Hirose T. Functional domains of NEAT1 architectural lncRNA induce paraspeckle assembly through phase separation. Mol. Cell. 2018; 70:1038–1053. 29932899 10.1016/j.molcel.2018.05.019

[125] Ilık İ.A. , Malszycki M., Lübke A.K., Schade C., Meierhofer D., Aktaş T. Son and srrm2 are essential for nuclear speckle formation. Elife. 2020; 9:e60579. 33095160 10.7554/eLife.60579 PMC7671692

[126] Tieg B. , Krebber H. Dbp5 - from nuclear export to translation. Biochim. Biophys. Acta - Gene Regul. Mech. 2013; 1829:791–798. 10.1016/j.bbagrm.2012.10.010 23128325

[127] Malet H. , Topf M., Clare D.K., Ebert J., Bonneau F., Basquin J., Drazkowska K., Tomecki R., Dziembowski A., Conti E. et al. RNA channelling by the eukaryotic exosome. EMBO Rep. 2010; 11:936–942. 21072061 10.1038/embor.2010.164 PMC2999861

[128] Wasmuth E.V. , Lima C.D. Exo- and endoribonucleolytic activities of yeast cytoplasmic and nuclear RNA exosomes are dependent on the noncatalytic core and central channel. Mol. Cell. 2012; 48:133–144. 22902556 10.1016/j.molcel.2012.07.012 PMC3472098

[129] Drążkowska K. , Tomecki R., Stoduś K., Kowalska K., Czarnocki-Cieciura M., Dziembowski A. The RNA exosome complex central channel controls both exonuclease and endonuclease dis3 activities in vivo and in vitro. Nucleic Acids Res. 2013; 41:3845–3858. 23404585 10.1093/nar/gkt060 PMC3616716

[130] Liu J.-J. , Bratkowski M.A., Liu X., Niu C.-Y., Ke A., Wang H.-W. Visualization of distinct substrate-recruitment pathways in the yeast exosome by eM. Nat. Struct. Mol. Biol. 2014; 21:95–102. 24336220 10.1038/nsmb.2736 PMC3976988

[131] Han J. , van Hoof A. The RNA exosome channeling and direct access conformations have distinct in vivo functions. Cell Rep. 2016; 16:3348–3358. 27653695 10.1016/j.celrep.2016.08.059 PMC5044803

[132] Delan-Forino C. , Schneider C., Tollervey D. RNA substrate length as an indicator of exosome interactions in vivo. Wellcome Open Res. 2017; 2:34. 28748221 10.12688/wellcomeopenres.10724.2 PMC5500899

[133] Delan-Forino C. , Schneider C., Tollervey D. Transcriptome-wide analysis of alternative routes for RNA substrates into the exosome complex. PLoS Genet. 2017; 13:e1006699. 28355211 10.1371/journal.pgen.1006699 PMC5389853

[134] Brouwer R. , Allmang C., Raijmakers R., van Aarssen Y., Egberts W.V., Petfalski E., van Venrooij W.J., Tollervey D., Pruijn G.J.M. Three novel components of the human exosome. J. Biol. Chem. 2001; 276:6177–6184. 11110791 10.1074/jbc.M007603200

[135] Yoshikatsu Y. , Ishida Y., Sudo H., Yuasa K., Tsuji A., Nagahama M. NVL2, a nucleolar AAA-ATPase, is associated with the nuclear exosome and is involved in pre-rRNA processing. Biochem. Biophys. Res. Commun. 2015; 464:780–786. 26166824 10.1016/j.bbrc.2015.07.032

[136] Chen C.-Y. , Gherzi R., Ong S.-E., Chan E.L., Raijmakers R., Pruijn G.J.M., Stoecklin G., Moroni C., Mann M., Karin M. AU binding proteins recruit the exosome to degrade ARE-Containing mRNAs. Cell. 2001; 107:451–464. 11719186 10.1016/s0092-8674(01)00578-5

[137] Tomecki R. , Drazkowska K., Kucinski I., Stodus K., Szczesny R.J., Gruchota J., Owczarek E.P., Kalisiak K., Dziembowski A. Multiple myeloma-associated hDIS3 mutations cause perturbations in cellular RNA metabolism and suggest hDIS3 PIN domain as a potential drug target. Nucleic Acids Res. 2014; 42:1270–1290. 24150935 10.1093/nar/gkt930 PMC3902924

[138] Tavanez J.P. , Calado P., Braga J., Lafarga M., Carmo-Fonseca M. In vivo aggregation properties of the nuclear poly(A)-binding protein PABPN1. RNA. 2005; 11:752–762. 15811916 10.1261/rna.7217105 PMC1370760

[139] Klein P. , Oloko M., Roth F., Montel V., Malerba A., Jarmin S., Gidaro T., Popplewell L., Perie S., Lacau St Guily J. et al. Nuclear poly(A)-binding protein aggregates misplace a pre-mRNA outside of SC35 speckle causing its abnormal splicing. Nucleic Acids Res. 2016; 44:10929–10945. 27507886 10.1093/nar/gkw703 PMC5159528

[140] Chujo T. , Yamazaki T., Kawaguchi T., Kurosaka S., Takumi T., Nakagawa S., Hirose T. Unusual semi-extractability as a hallmark of nuclear body-associated architectural noncoding RNAs. EMBO J. 2017; 36:1447–1462. 28404604 10.15252/embj.201695848 PMC5430218

[141] Iasillo C. , Schmid M., Yahia Y., Maqbool M.A., Descostes N., Karadoulama E., Bertrand E., Andrau J.-C., Jensen T.H. ARS2 is a general suppressor of pervasive transcription. Nucleic Acids Res. 2017; 45:10229–10241. 28973446 10.1093/nar/gkx647 PMC5622339

[142] Wang R. , Zheng D., Wei L., Ding Q., Tian B. Regulation of intronic polyadenylation by PCF11 impacts mRNA expression of long genes. Cell Rep. 2019; 26:2766–2778. 30840896 10.1016/j.celrep.2019.02.049 PMC6428223

[143] Gruber J.J. , Olejniczak S.H., Yong J., La Rocca G., Dreyfuss G., Thompson C.B. Ars2 promotes proper replication-dependent histone mRNA 3′ end formation. Mol. Cell. 2012; 45:87–98. 22244333 10.1016/j.molcel.2011.12.020 PMC3269315

[144] Narita T. , Yung T.M.C., Yamamoto J., Tsuboi Y., Tanabe H., Tanaka K., Yamaguchi Y., Handa H. NELF interacts with CBC and participates in 3′ end processing of replication-dependent histone mRNAs. Mol. Cell. 2007; 26:349–365. 17499042 10.1016/j.molcel.2007.04.011

[145] Fan J. , Wang K., Du X., Wang J., Chen S., Wang Y., Shi M., Zhang L., Wu X., Zheng D. et al. ALYREF links 3’-end processing to nuclear export of non-polyadenylated mRNAs. EMBO J. 2019; 38:e99910. 30858280 10.15252/embj.201899910 PMC6484419

[146] Gockert M. , Schmid M., Jakobsen L., Jens M., Andersen J.S., Jensen T.H. Rapid factor depletion highlights intricacies of nucleoplasmic RNA degradation. Nucleic Acids Res. 2022; 50:1583–1600. 35048984 10.1093/nar/gkac001 PMC8860595

[147] Hilleren P. , McCarthy T., Rosbash M., Parker R., Jensen T.H. Quality control of mRNA 3′-end processing is linked to the nuclear exosome. Nature. 2001; 413:538–542. 11586364 10.1038/35097110

[148] Wang Y. , Fan J., Wang J., Zhu Y., Xu L., Tong D., Cheng H. 2021) ZFC3H1 prevents RNA trafficking into nuclear speckles through condensation. Nucleic Acids Res. 49:10630–10643. 34530450 10.1093/nar/gkab774 PMC8501945

[149] Kudo N. , Matsumori N., Taoka H., Fujiwara D., Schreiner E.P., Wolff B., Yoshida M., Horinouchi S. Leptomycin b inactivates CRM1/exportin 1 by covalent modification at a cysteine residue in the central conserved region. Proc. Natl. Acad. Sci. U.S.A. 1999; 96:9112–9117. 10430904 10.1073/pnas.96.16.9112 PMC17741

[150] McCloskey A. , Taniguchi I., Shinmyozu K., Ohno M. hnRNP c tetramer measures RNA length to classify RNA polymerase II transcripts for export. Science. 2012; 335:1643–1646. 22461616 10.1126/science.1218469

