# Supplementary material for: MPP6 stimulates both RRP6 and DIS3 to degrade a specified subset of MTR4-sensitive substrates in the human nucleus
Source: Nucleic Acids Res. 2022 Jul 29;50(15):8779–806. doi: 10.1093/nar/gkac559 (PMC9410898; doi:10.1093/nar/gkac559)
Supplement: gkac559_Supplemental_Files [file gkac559_supplemental_files.zip › Captions(Table S1 and S2) by N. Fujiwara et al.docx]

Table S1; Materials used in this study

Table S2; Classification of RRP6/DIS3 KD stabilized substrates according to their sensitivities to each nuclease
