# Supplementary material for: MPP6 stimulates both RRP6 and DIS3 to degrade a specified subset of MTR4-sensitive substrates in the human nucleus
Source: Nucleic Acids Res. 2022 Jul 29;50(15):8779–806. doi: 10.1093/nar/gkac559 (PMC9410898; doi:10.1093/nar/gkac559)
Supplement: gkac559_Supplemental_Files [file gkac559_supplemental_files.zip › Figure S1,S2.pdf]

Figure S1

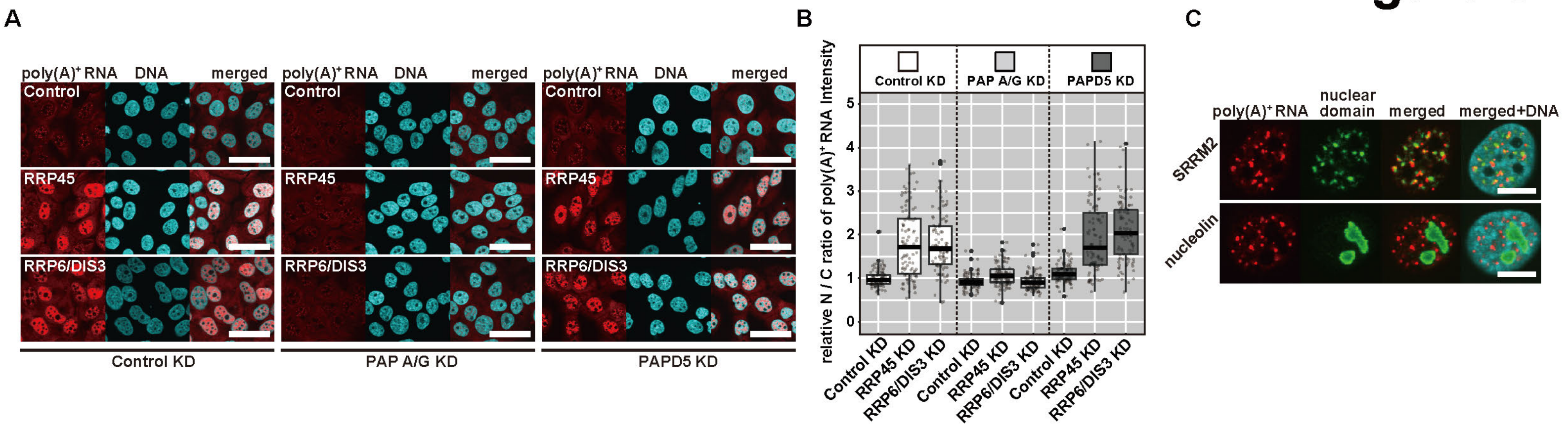
A
poly(A)+ RNA
DNA
merged
Control
RRP45
RRP6/DIS3
Control KD
PAP A/G KD
PAPD5 KD
B
Control KD
PAP A/G KD
PAPD5 KD
relative N / C ratio of poly(A)+ RNA Intensity
0
1
2
3
4
5
Control KD
RRP45 KD
RRP6/DIS3 KD
C
poly(A)+ RNA
nuclear domain
merged
merged+DNA
SRRM2
nucleolin

Figure S1. Nuclear specific inhibition of the exosome leads to a prominent accumulation of poly(A)$^+$ RNA in the human nucleoplasm. (A), (B) Exosome-inhibition derived nuclear accumulation of poly(A)$^+$ RNAs are restored by the PAP A/G KD , but not by PAPD5 KD. (C) Exclusion of RRP6/DIS3 KD-provoked poly(A)$^+$ foci from the nucleolus. (A) Poly(A)$^+$ FISH experiments. Depleted factors are indicated in panels and at the bottom. Scale bar = 50 μm. (B) Quantification of (A). Relative nuclear/cytoplasmic (N/C) ratio of poly(A)$^+$ FISH signal intensity normalized by the mean value of Control KD/Control KD sample are shown. *n* = 100. (C) SRRM2 was stained as a speckle marker and nucleolin as a nucleolar marker. Scale bar = 10 μm.

Figure S2

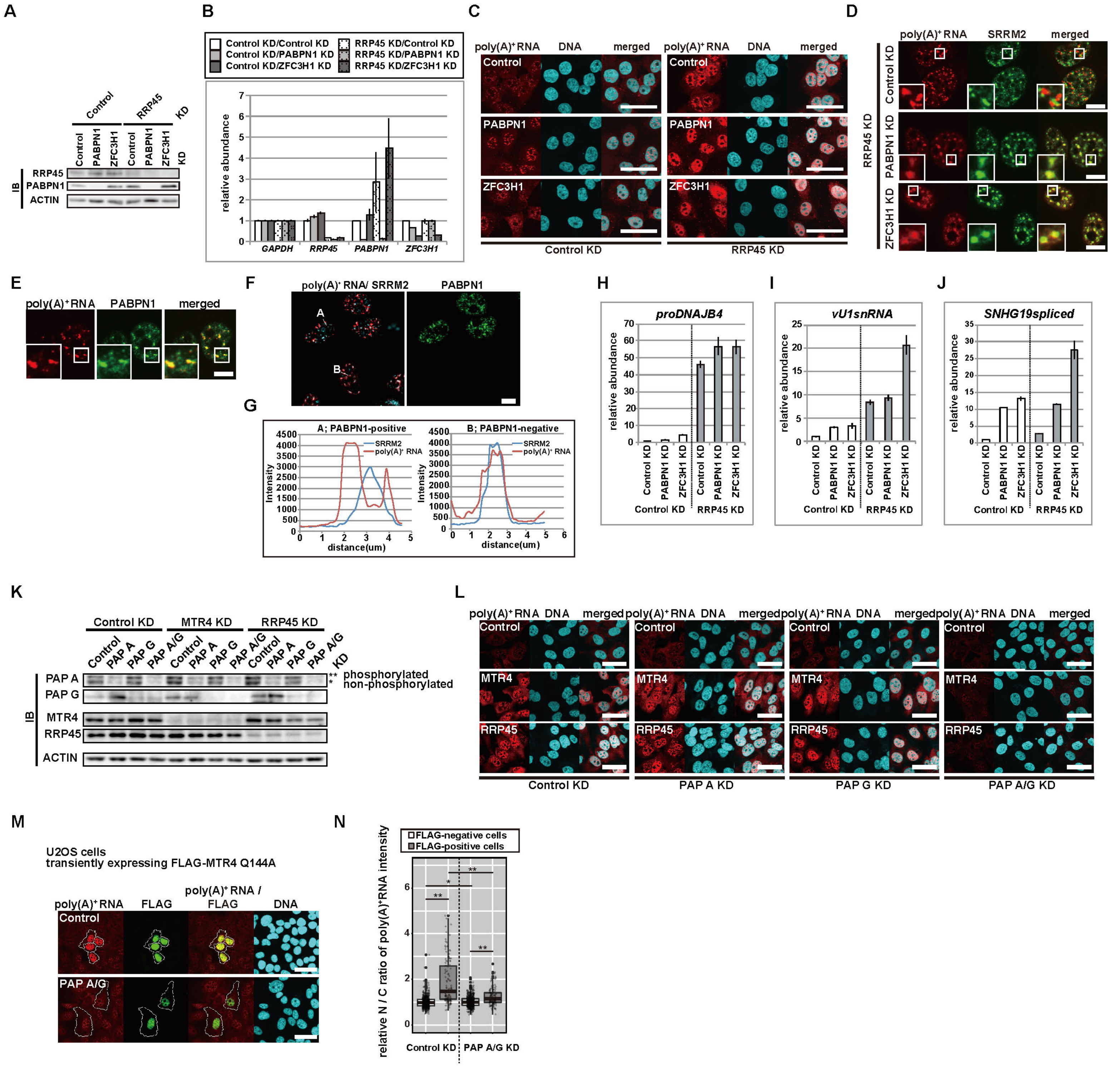

A
B
C
D
E
F
G
H
I
J
K
L
M
N
Control KD/Control KD
Control KD/PABPN1 KD
Control KD/ZFC3H1 KD
RRP45 KD/Control KD
RRP45 KD/PABPN1 KD
RRP45 KD/ZFC3H1 KD
relative abundance
GAPDH
RRP45
PABPN1
ZFC3H1
poly(A)⁺ RNA
DNA
merged
SRRM2
Control KD
RRP45 KD
PABPN1 KD
ZFC3H1 KD
poly(A)⁺ RNA/ SRRM2
PABPN1
A; PABPN1-positive
B; PABPN1-negative
Intensity
distance(um)
proDNAJB4
vU1snRNA
SNHG19spliced
MTR4 KD
PAP A
PAP G
PAP A/G
phosphorylated
non-phosphorylated
IB
ACTIN
RRP45
MTR4
PAP A KD
PAP G KD
PAP A/G KD
U2OS cells
transiently expressing FLAG-MTR4 Q144A
poly(A)⁺ RNA / FLAG
FLAG
FLAG-negative cells
FLAG-positive cells
relative N / C ratio of poly(A)⁺RNA intensity

Figure S2. Properties of exosome poly(A)$^+$ substrates in U2OS cells upon PAXT depletion. (A)-(J) The effect of ZFC3H1 KD and PABPN1 KD on the poly(A)$^+$ substrate decay. (K)-(N) Poly(A)$^+$ substrates stabilized by MTR4 dysfunction are poly(A)$^+$ tailed by PAP A/G. (A), (K) Immunoblot analysis to confirm specific depletion of factors indicated at the top of the panels. In (K), the asterisk (*) indicates signals from non-phosphorylated PAP A and a double asterisk (**) indicates signals from phosphorylated PAP A. (B) RT-qPCRs to confirm specific depletion of factors noted at the bottom. Bars are color-coded according to the KD conditions, as described in the inset. Relative abundances of each transcript normalized to *GAPDH* and the value of Control KD/Control KD sample are shown. Bars and error bars represent mean values ± *SD*. *n* = 3. PCRs were performed on cDNA synthesized using dT_{25} to total RNAs extracted from whole cells. (C), (L) Poly(A)$^+$ FISH experiments. Knocked down factors are listed in the panels and at the bottom. Scale bar = 50 μm. (D) PAXT depletion changes the localization of poly(A)$^+$ RNA stabilized by exosome inhibition to the speckles. Nuclear speckles and poly(A)$^+$ RNAs were visualized simultaneously. Factors depleted are stated to the left of the panels. Magnified pictures are shown in insets. Scale bar = 10 μm. (E) Accumulation of PABPN1 to poly(A)$^+$ foci in U2OS cells derived from RRP45 KD. PABPN1 was visualized by immunostaining using a specific antibody along with poly(A)$^+$ RNAs. Magnified pictures are shown in insets. Scale bar = 10 μm. (F) Localization of RRP45 KD-derived poly(A)$^+$ RNA was compared between PABPN1-positive and PABPN1-negative U2OS cells. The emergence of PABPN1-positive and PABPN1-negative RRP45 KD cells was achieved by the second transfection of siRNA against PABPN1 into cells that had been previously depleted of RRP45. The second round siRNA transfection was at 6 h after the first siRNA transfection and cells were fixed at 72 h after the first transfection. PABPN1 was visualized along with poly(A)$^+$ RNAs. Scale bar = 10 μm. (G) Line profiles of signal intensities from poly(A)$^+$ FISH signal and from SRRM2 immunofluorescence signal along the drawn line in (F). Line A represents the poly(A)$^+$ aggregates in PABPN1-positive cells and line B represents those in PABPN1-negative cells. (H)-(J) RT-qPCRs were performed using dT_{25} primed cDNA derived from total RNA extracted from whole cells to quantify (H) *proDNAJB4*, (I) *vU1snRNA* and (J) spliced *SNHG19*. Factors depleted from cells are described at the bottom. Presented values are relative abundances of each transcript normalized to *GAPDH* and the value of Control KD/Control KD sample. Bars and error bars denote mean values ± *SD*. *n* = 3. (L) Simultaneous depletion of PAP A/G restored the nuclear poly(A)$^+$ RNA accumulating phenotype derived from MTR4 KD in U2OS cells. Depleted factors are shown in the panel and at the bottom. Scale bar = 50 μm. (M), (N) Subcellular localization of poly(A)$^+$

RNAs. Conditions of cell lines, expressed proteins and depleted factors are stated at the top of and in panels. Transiently expressed FLAG-MTR4 proteins were visualized by FLAG-staining, and cells were categorized as either “FLAG-positive” or “FLAG-negative” cells using the Random Forest classifier in CellProfiler Analyst. White lines outline “FLAG-positive” cells. Scale bar = 50 μm. (N) Quantification of (M). Nuclear/cytoplasmic (N/C) ratios of poly(A)$^+$ FISH signal were normalized by the mean value of Control KD “FLAG-negative” cells. Statistical analysis was performed within each cell line using Steel-Dwass test following Kruskal-Wallis test. $^{**}p < 0.01$, ns : not significant, FLAG-negative Control KD; $n = 157$, FLAG-positive Control KD; $n = 126$, FLAG-negative PAP A/G KD; $n = 669$, FLAG-positive PAP A/G KD; $n = 131$.
