# Supplementary material for: MPP6 stimulates both RRP6 and DIS3 to degrade a specified subset of MTR4-sensitive substrates in the human nucleus
Source: Nucleic Acids Res. 2022 Jul 29;50(15):8779–806. doi: 10.1093/nar/gkac559 (PMC9410898; doi:10.1093/nar/gkac559)
Supplement: gkac559_Supplemental_Files [file gkac559_supplemental_files.zip › Figure S10.pdf]

Figure S10

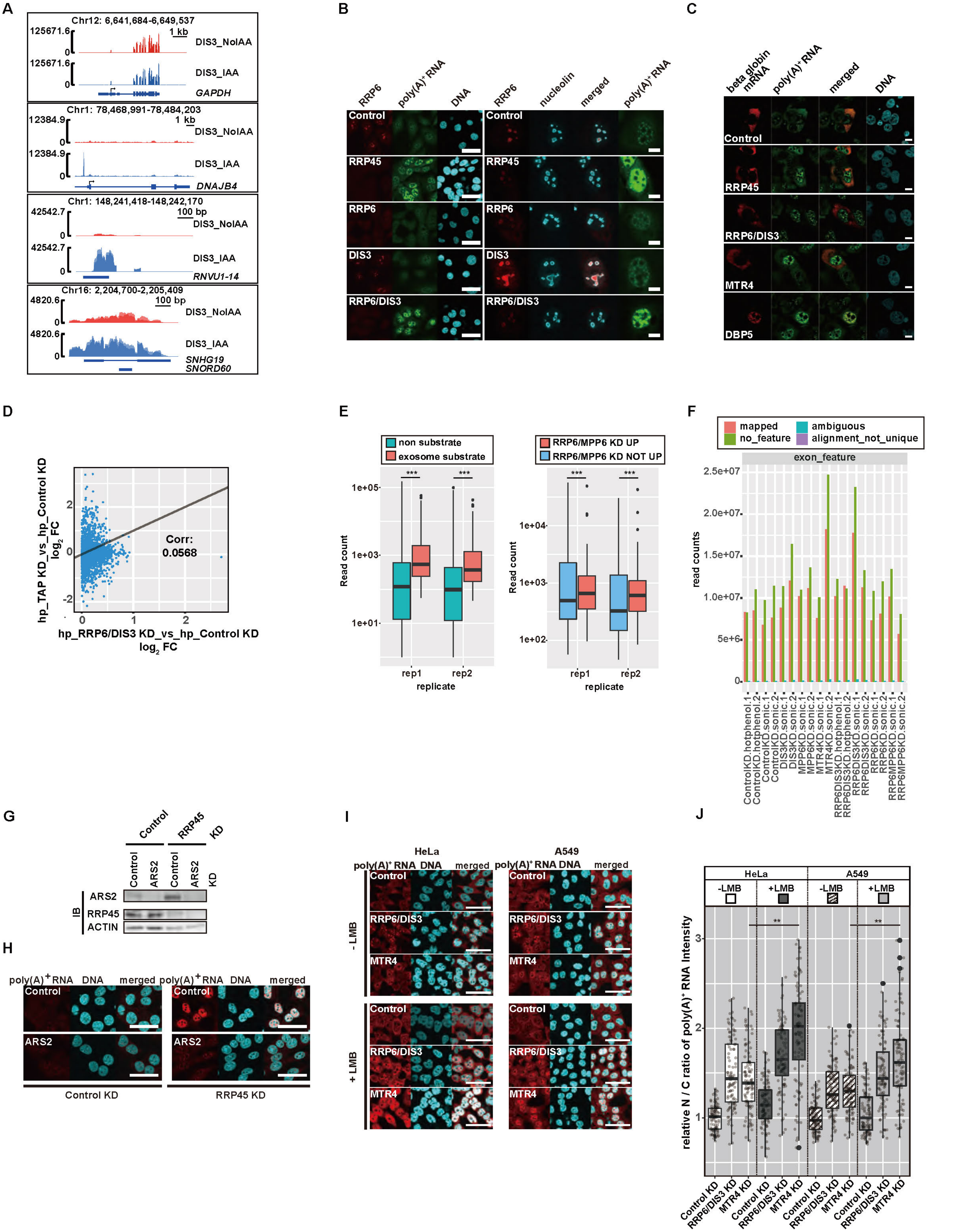

A
Chr12: 6,641,684-6,649,537
1 kb
DIS3_NoIAA
DIS3_IAA
GAPDH
Chr1: 78,468,991-78,484,203
DNAJB4
Chr1: 148,241,418-148,242,170
100 bp
RNVU1-14
Chr16: 2,204,700-2,205,409
SNHG19
SNORD60
B
RRP6
poly(A)+ RNA
DNA
nucleolin
merged
Control
RRP45
RRP6
DIS3
RRP6/DIS3
C
beta globin mRNA
poly(A)+ RNA
merged
DNA
MTR4
DBP5
D
hp_TAP KD_vs_hp_Control KD log2 FC
hp_RRP6/DIS3 KD_vs_hp_Control KD log2 FC
Corr: 0.0568
E
non substrate
exosome substrate
RRP6/MPP6 KD UP
RRP6/MPP6 KD NOT UP
Read count
replicate
rep1
rep2
F
mapped
no_feature
ambiguous
alignment_not_unique
exon_feature
read counts
G
Control
RRP45
KD
ARS2
IB
RRP45
ACTIN
H
poly(A)+ RNA
DNA
merged
Control KD
RRP45 KD
I
HeLa
A549
- LMB
+ LMB
J
relative N / C ratio of poly(A)+ RNA Intensity
-LMB
+LMB
Control KD
RRP6/DIS3 KD
MTR4 KD

Figure S10. Vigorous role of DIS3 in nucleoplasmic substrate decay, possible causes of poly(A)$^+$ aggregates and enrichment of short TU transcripts among exosome poly(A)$^+$ substrates. (A) Stabilization of the substrates identified in this study upon rapid DIS3 depletion. (B) The effect of depleting exosome components on the subcellular localization of RRP6. (C), (D) Exosome inhibition does not compromise global mRNA transport. (E) Read counts per gene in each substrate class under RRP6/DIS3 KD condition. (F) The proportion of reads subjected to differential expression analysis. (G), (H) ARS2 KD mitigates the nuclear poly(A)$^+$ RNA accumulating phenotype derived from exosome inhibition. (I), (J) Leptomycin B (LMB) treatment significantly increases the nuclear/cytoplasmic (N/C) ratio of poly(A)$^+$ RNA distribution in MTR4 KD HeLa and A549 cells. (A) Genome browser views of the identified substrates. The location in the genome is noted at the top of the tracks, with the gene name and structure at the bottom. Read counts are shown at the left of the tracks. DIS3_NoIAA denotes control samples, and DIS3_IAA indicates DIS3 depleted samples. Overlays of the two replicates are shown. *GAPDH* was used as a control. (B) Endogenous RRP6 was simultaneously visualized with poly(A)$^+$ RNAs in the left panels. On the right panels, nucleolin was also visualized. Conditions of transfected siRNAs are indicated in the panels. Scale bar = 50 μm in the left panels and 10μm in the right panels. (C) Beta-globin mRNAs and poly(A)$^+$ RNAs were visualized using the Alexa594 conjugated specific probe and the Alexa488 dT_{45} probe, respectively. Depleted factors are stated in each panel. Scale bar = 10 μm. (D) A poor correlation between the nuclear poly(A)$^+$ RNA profiles of the exosome-inhibited cells and the mRNA transport-impaired cells. (D) Log_2 FC induced by RRP6/DIS3 KD was plotted against that induced by TAP KD. Spearman's rank correlation coefficients are stated in the panels. The line in the figure shows y = x. (E) Read counts were compared between the RRP6/DIS3 KD and the RRP6/DIS3 KD NOT stabilized substrates, i.e., between ”exosome substrate” and ”non-substrate” in the left panel, and between the RRP6/MPP6 KD stabilized and NOT stabilized subclasses on the right. Counts were compared within each replicate (rep1 and rep2). Statistical analysis was conducted using the Wilcoxon rank-sum test with continuity correction. (F) Bar plot of the read count output from the HT-seq exon feature count. Read counts mapped to the "exon" feature (mapped) and those mapped outside this feature (no_featurer, ambiguous and alignment_not_unique) were summed by each category. Because we applied a unique mapping, no alignment_not_unique reads were obtained. (G) Immunoblot analysis to confirm the specific KD of factors indicated at the top of the panels. (H) Poly(A)$^+$ FISH analysis of U2OS cells deprived of factors stated in the panels and at the bottom. Scale bar = 50 μm. (I) Poly(A)$^+$ FISH analysis. The conditions of the LMB treatment

are shown on the left and depleted factors in the panels. Scale bar = 50 µm. (J) Quantification of (I). Presented values are relative N/C ratios of poly(A)$^+$ FISH signals normalized within each cell line by Control KD samples without LMB treatment. Statistical analysis was conducted using the Kruskal-Wallis test followed by the Steel-Dwass test. **$p < 0.01$, $n = 100$.
