# Supplementary material for: MPP6 stimulates both RRP6 and DIS3 to degrade a specified subset of MTR4-sensitive substrates in the human nucleus
Source: Nucleic Acids Res. 2022 Jul 29;50(15):8779–806. doi: 10.1093/nar/gkac559 (PMC9410898; doi:10.1093/nar/gkac559)
Supplement: gkac559_Supplemental_Files [file gkac559_supplemental_files.zip › Figure S3.pdf]

Figure S3

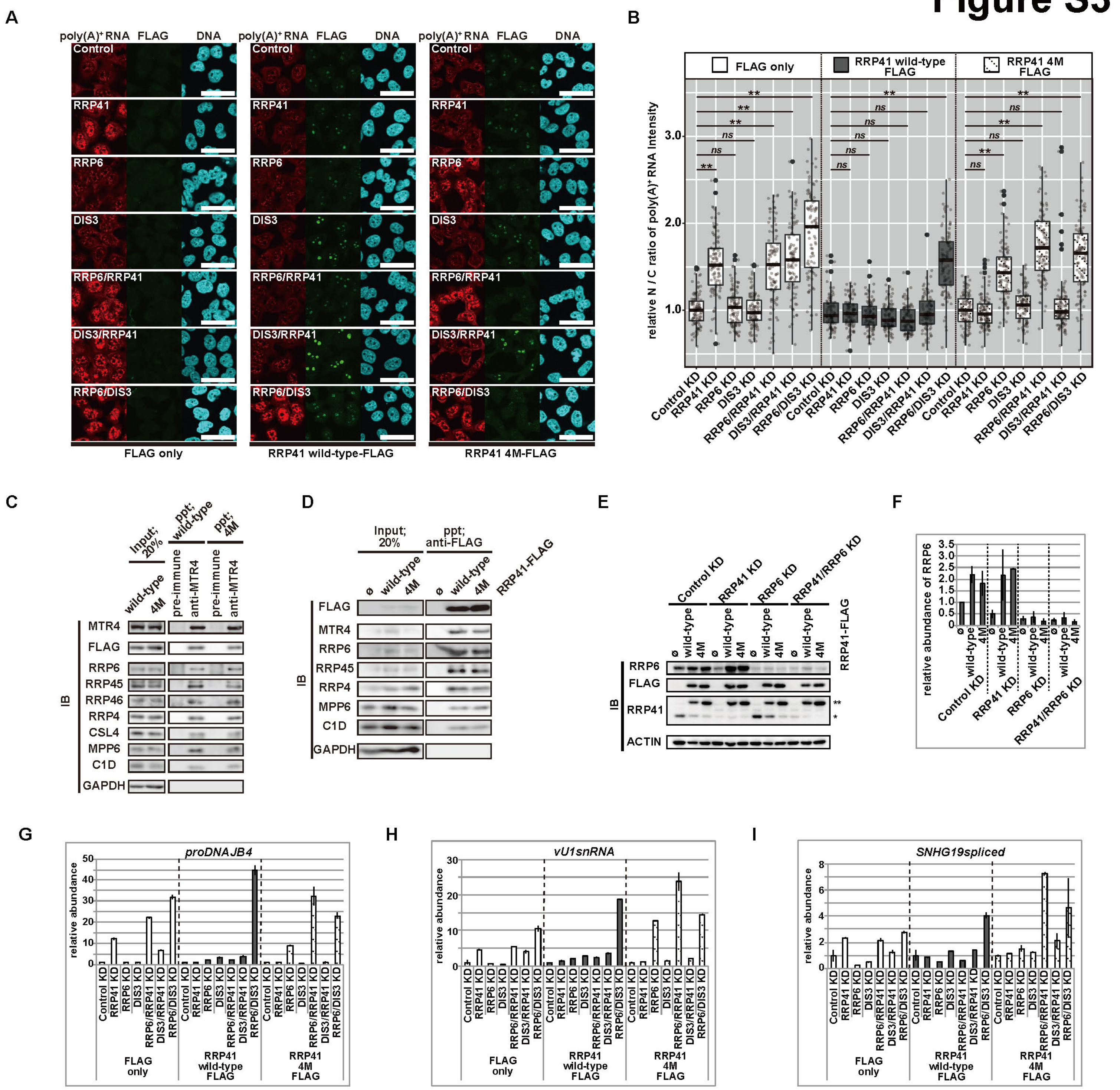
A
poly(A)+ RNA FLAG DNA
Control
RRP41
RRP6
DIS3
RRP6/RRP41
DIS3/RRP41
RRP6/DIS3
FLAG only
RRP41 wild-type-FLAG
RRP41 4M-FLAG
B
FLAG only
RRP41 wild-type FLAG
RRP41 4M FLAG
relative N / C ratio of poly(A)+ RNA Intensity
Control KD
RRP41 KD
RRP6 KD
DIS3 KD
RRP6/RRP41 KD
DIS3/RRP41 KD
RRP6/DIS3 KD
C
Input; 20%
ppt; wild-type
ppt; 4M
wild-type
4M
pre-immune
anti-MTR4
IB
MTR4
FLAG
RRP6
RRP45
RRP46
RRP4
CSL4
MPP6
C1D
GAPDH
D
Input; 20%
ppt; anti-FLAG
RRP41-FLAG
ø
wild-type
4M
E
Control KD
RRP41 KD
RRP6 KD
RRP41/RRP6 KD
RRP41-FLAG
ACTIN
F
relative abundance of RRP6
G
proDNAJB4
relative abundance
H
vU1snRNA
I
SNHG19spliced
FLAG only
RRP41 wild-type FLAG
RRP41 4M FLAG

Figure S3. Poly(A)$^+$ substrates reach DIS3 through the central channel.

(A), (B) The subcellular distribution of poly(A)$^+$ RNAs was visualized by *in situ* hybridization using Alexa594 labeled dT_{45} probe [poly(A)$^+$ FISH]. (C), (D) Immunoprecipitation analysis to confirm that exogenously expressed RRP41-FLAGs are efficiently incorporated into the exosome complex. (E), (F) Immunoblot analysis of the nuclear extracts to confirm the specific depletion of each exosome component. (G)-(I) RT-qPCRs to quantify known representative substrates of the nuclear exosome. (A) Expressed proteins were simultaneously visualized with poly(A)$^+$ RNAs by FLAG-staining. Conditions of transfected siRNAs and used cell lines are indicated at the bottom of and in the panels. Scale bar = 50 μm. (B) Quantification of the nuclear/cytoplasmic (N/C) ratio of poly(A)$^+$ FISH signal intensity from the experiment in (A). Relative values normalized by the mean value of Control KD within each cell line are shown. Statistical analysis was performed using Kruskal-Wallis test followed by Steel test. *$p < 0.05$, **$p < 0.01$, *ns*: not significant, $n = 100$. (C), (D) Immunoprecipitation experiments were performed using (C) anti-MTR4 antiserum and (D) anti-FLAG antibody. Rabbit pre-immune serum and anti-HA antibody were used as controls in (C) and (D), respectively. (E), (F) Immunoblot analysis. Conditions of transfected siRNAs and used cell lines are as indicated in the upper notes. In (E), the asterisk (*) indicates signals from endogenous RRP41 and a double asterisk (**) indicates signals from exogenously expressed RRP41-FLAG. A significant reduction in endogenous RRP41 protein level was observed when either wild-type or 4M RRP41-FLAG was expressed, indicating the autoregulation of RRP41 abundance. (F) Quantification of two independent Immunoblot analyses including (E). The relative abundance of RRP6 was normalized to that of ACTIN. Values were also normalized to that of FLAG-only expressing Control KD cells. Cell lines and conditions are indicated in the bottom notes. Bars and error bars denote mean values ± *SD*. (G)-(I) PCRs were performed on cDNA synthesized using dT_{25} to total RNAs extracted from whole cells. Cell lines and conditions are indicated at the bottom. The relative abundance of (G) *proDNAJB4*, (H) *vU1snRNA*, (I) spliced *SNHG19*, normalized by that of *GAPDH* and by the value of Control KD sample within each cell line, are shown. Bars and error bars denote mean values ± *SD*. $n = 3$.
