# Supplementary material for: MPP6 stimulates both RRP6 and DIS3 to degrade a specified subset of MTR4-sensitive substrates in the human nucleus
Source: Nucleic Acids Res. 2022 Jul 29;50(15):8779–806. doi: 10.1093/nar/gkac559 (PMC9410898; doi:10.1093/nar/gkac559)
Supplement: gkac559_Supplemental_Files [file gkac559_supplemental_files.zip › Figure S4.pdf]

Figure S4

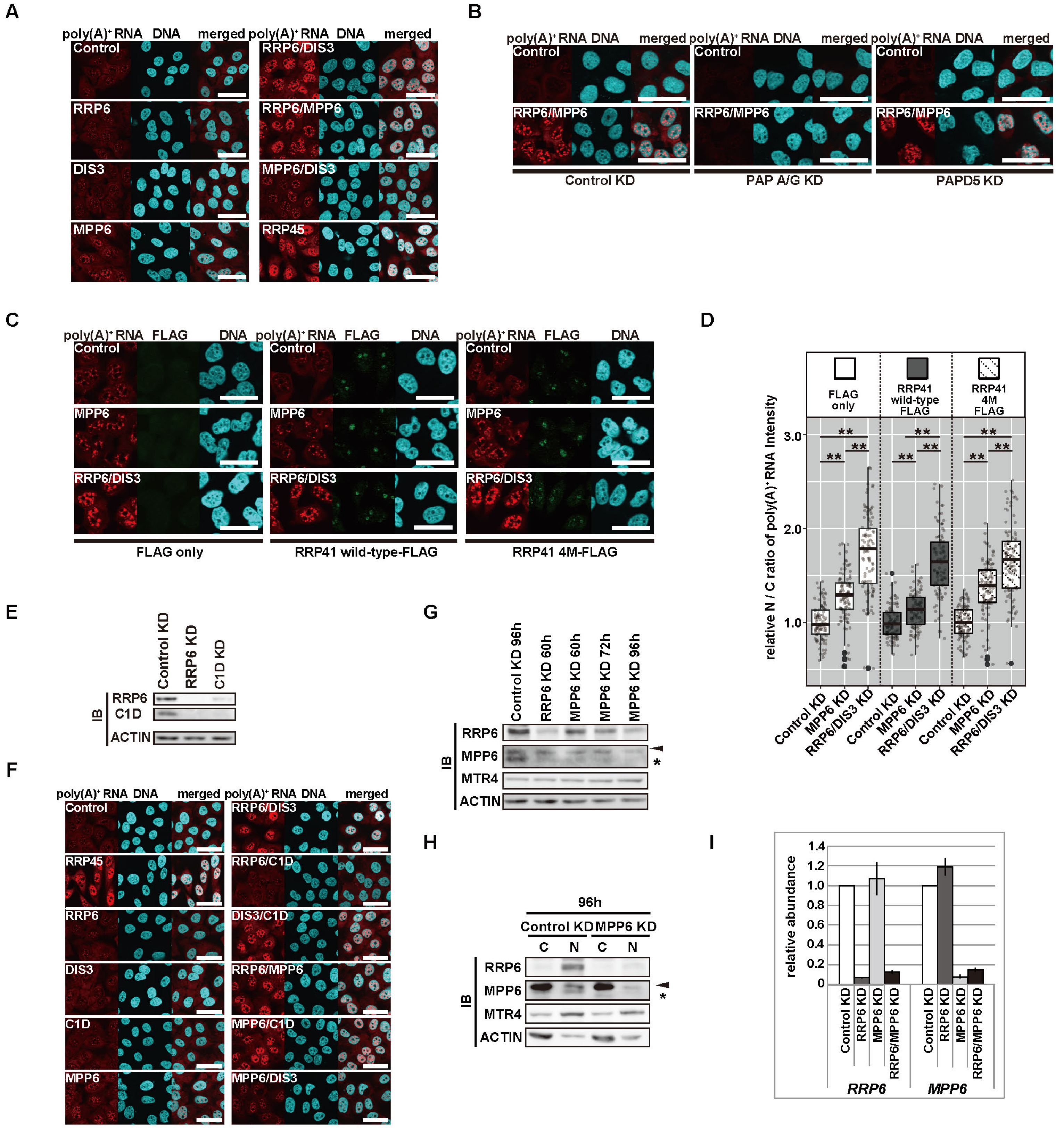
A
poly(A)+ RNA DNA merged
Control
RRP6
DIS3
MPP6
RRP6/DIS3
RRP6/MPP6
MPP6/DIS3
RRP45
B
Control
RRP6/MPP6
Control KD
PAP A/G KD
PAPD5 KD
C
poly(A)+ RNA FLAG DNA
FLAG only
RRP41 wild-type-FLAG
RRP41 4M-FLAG
D
FLAG only
RRP41 wild-type FLAG
RRP41 4M FLAG
relative N / C ratio of poly(A)+ RNA intensity
3.0
2.0
1.0
Control KD
MPP6 KD
RRP6/DIS3 KD
E
Control KD
RRP6 KD
C1D KD
IB
RRP6
C1D
ACTIN
F
RRP6/C1D
DIS3/C1D
C1D
MPP6/C1D
MPP6/DIS3
G
Control KD 96h
RRP6 KD 60h
MPP6 KD 60h
MPP6 KD 72h
MPP6 KD 96h
MTR4
H
96h
Control KD
MPP6 KD
C N C N
I
relative abundance
1.4
1.2
1.0
0.8
0.6
0.4
0.2
0
RRP6/MPP6 KD
RRP6
MPP6

Figure S4. MPP6 functions redundantly with RRP6 to degrade nucleoplasmic poly(A)$^+$ substrates. (A) The effect of MPP6 depletion on bulk poly(A)$^+$ substrate decay in HeLa cells. (B) PAP A/G, but not PAPD5, adenylate substrates responsible for poly(A)$^+$ foci formation in RRP6/MPP6 KD cells. (C), (D) The decay of poly(A)$^+$ substrates by RRP6 is not completely, but partially attenuated upon MPP6 KD. (E), (F) Depletion of C1D simultaneously with MPP6 also led to the poly(A)$^+$ substrate stabilization. (G)-(I) Post-transcriptional down-regulation of MPP6 under the RRP6 KD condition and *vice versa*. (A), (B), (C), (F) Poly(A)$^+$ FISH experiments. In (C), expressed proteins were visualized by FLAG-staining. Conditions of knockdown and cell lines are stated in and at the bottom of the panels. Scale bar = 50 μm. (D) Quantification of (C). Relative nuclear/cytoplasmic (N/C) ratio of poly(A)$^+$ FISH signal normalized to the mean value of Control KD cells within each cell line. Statistical analysis was performed using Steel-Dwass test following Kruskal-Wallis test. **$p < 0.01$, $n = 100$. (E) Interdependent expression of RRP6 and C1D. Immunoblot analysis was performed to the nuclear extracts from RRP6 KD U2OS cells and from C1D KD U2OS cells. (F) Poly(A)$^+$ FISH under conditions of C1D depletion solely or simultaneously with RRP6, DIS3 or MPP6. Factors depleted in U2OS cells are stated in the panels. Scale bar = 50 μm. (G), (H) Immunoblot analysis was performed on the nuclear extracts in (G) and to both the cytoplasmic and the nuclear extracts in (H) [noted at the top of the panel in (H) as "C" and "N", respectively]. The notes at the top of the panel also indicate the depleted factors and the time elapsed since siRNA transfection. The asterisk (*) denotes signals from endogenous MPP6. Arrowhead denotes a non-specific band. In (H), the higher distribution of MTR4 in the nucleus and ACTIN in the cytoplasm validated a proper cellular fractionation. (I) MPP6 KD has little effect on the transcription of RRP6 and *vice versa*. RT-qPCRs were performed on random9-primed cDNAs synthesized using total RNAs from cells deprived of factors listed below the bar chart for 96 hours, to quantify the transcripts described at the bottom, namely, *RRP6* and *MPP6*. Values are shown as the relative abundance of each transcript normalized by *GAPDH* and Control KD value. Bars and error bars denote mean values ± *SD*. n = 3.
