# Supplementary material for: MPP6 stimulates both RRP6 and DIS3 to degrade a specified subset of MTR4-sensitive substrates in the human nucleus
Source: Nucleic Acids Res. 2022 Jul 29;50(15):8779–806. doi: 10.1093/nar/gkac559 (PMC9410898; doi:10.1093/nar/gkac559)
Supplement: gkac559_Supplemental_Files [file gkac559_supplemental_files.zip › Figure S5,S6.pdf]

Figure S5

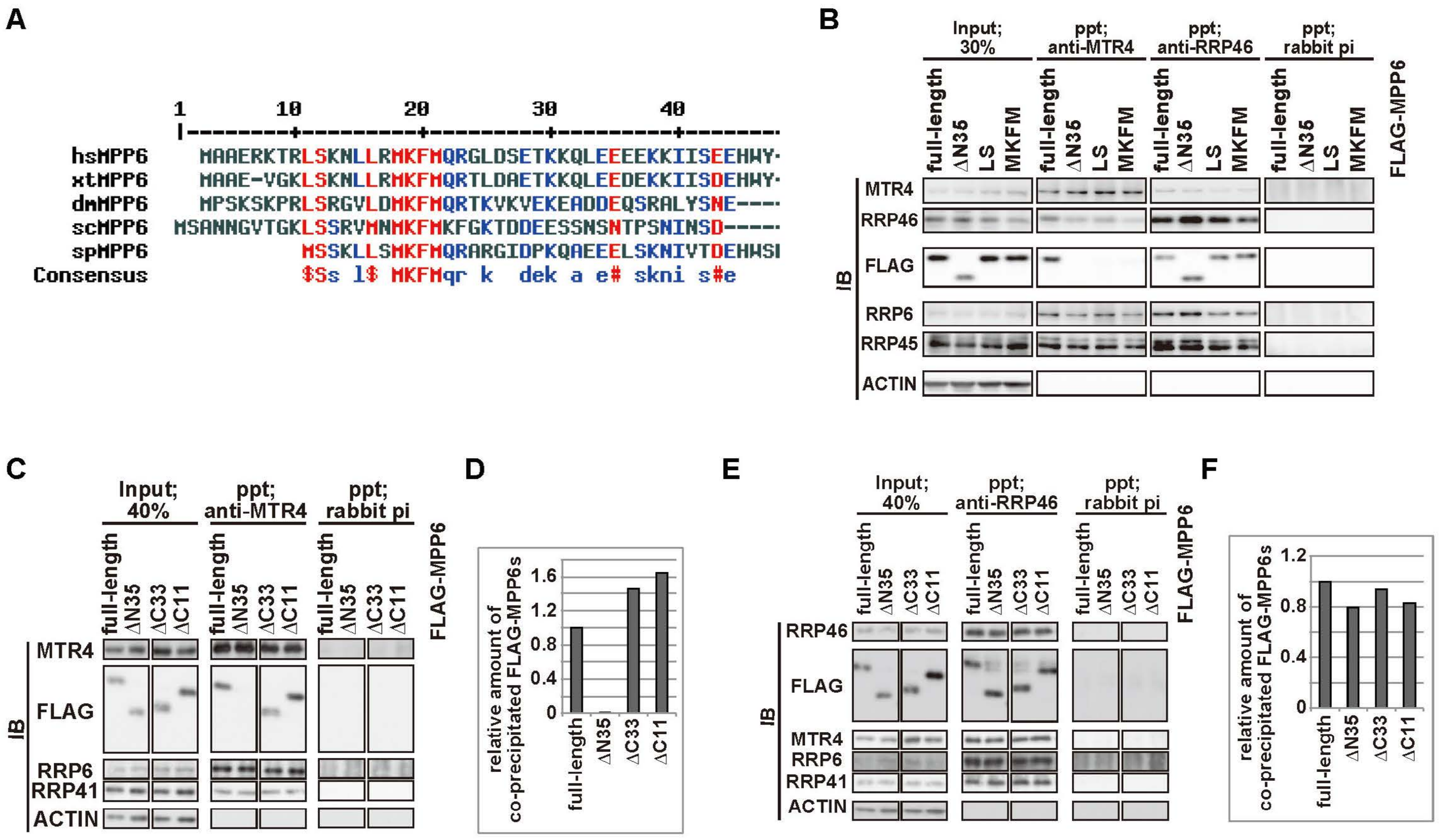
A
hsMPP6 MAAERKTRLSKNLLRMKFMQRGLDSETKKQLEEEEKKIISEEHWY
xtMPP6 MAAE-VGKLSKNLLRMKFMQRTLDAETKKQLEEDEKKIISDEHWY
dmMPP6 MPSKSKPRLSRGVLDMKFMQRTKVKVEKEADDEQSRALYSNE----
scMPP6 MSANNGVTGKLSSRVMNMKFMKFGKTDDEESSNSNTPSNINSD----
spMPP6 MSSKLLSMKFMQRARGIDPKQAEEELSKNIVTDEHWSI
Consensus $Ss l$ MKFMqr k dek a e# skni s#e
B
Input; 30%
ppt; anti-MTR4
ppt; anti-RRP46
ppt; rabbit pi
full-length
ΔN35
LS
MKFM
FLAG-MPP6
IB
MTR4
RRP46
FLAG
RRP6
RRP45
ACTIN
C
Input; 40%
ppt; anti-MTR4
ppt; rabbit pi
full-length
ΔN35
ΔC33
ΔC11
FLAG-MPP6
IB
MTR4
FLAG
RRP6
RRP41
ACTIN
D
relative amount of co-precipitated FLAG-MPP6s
0
0.4
0.8
1.2
1.6
full-length
ΔN35
ΔC33
ΔC11
E
Input; 40%
ppt; anti-RRP46
ppt; rabbit pi
full-length
ΔN35
ΔC33
ΔC11
FLAG-MPP6
IB
RRP46
FLAG
MTR4
RRP6
RRP41
ACTIN
F
relative amount of co-precipitated FLAG-MPP6s
0
0.4
0.8
1.2
full-length
ΔN35
ΔC33
ΔC11

Figure S5. Dissecting domains and residues within MPP6 responsible for binding with the exosome components. (A) Sequence conservation within the N-terminal region of MPP6. (B) Interaction of N-terminal MPP6 mutants with MTR4 and the exosome core. (C)-(F) Deleting the C-terminal region from MPP6 has little effect on its binding either to MTR4 or to the exosome core. (A) Alignment was carried out by Multalin v5.4.1. The sequences of MPP6 homologs in *hs*: *Homo sapiens*, *xt*: *Xenopus tropicalis*, *dm*: *Drosophila melanogaster*, *sc*: *Saccharomyces cerevisiae*, and *sp*: *Schizosaccharomyces pombe* were used as input. (B), (C), (E) Immunoprecipitation experiments to the nuclear extracts from HeLa Flp-In T-REx cells expressing FLAG-fused MPP6 mutants using anti-MTR4 antiserum in (B) and (C), and anti-RRP46 antibody in (B) and (E). Rabbit pre-immune antiserum was used as a control in the immunoprecipitation step. Cell lines are stated at the top of the panels. (D), (F) Quantification of (C) and (E), respectively. The ratio of FLAG-MPP6 either to MTR4 in (D) or to RRP46 in (F) in each precipitate was normalized by the respective ratio in each input extract and by the value of the FLAG-MPP6 full-length sample to obtain the relative precipitated amount of the corresponding FLAG-MPP6 mutant.

Figure S6

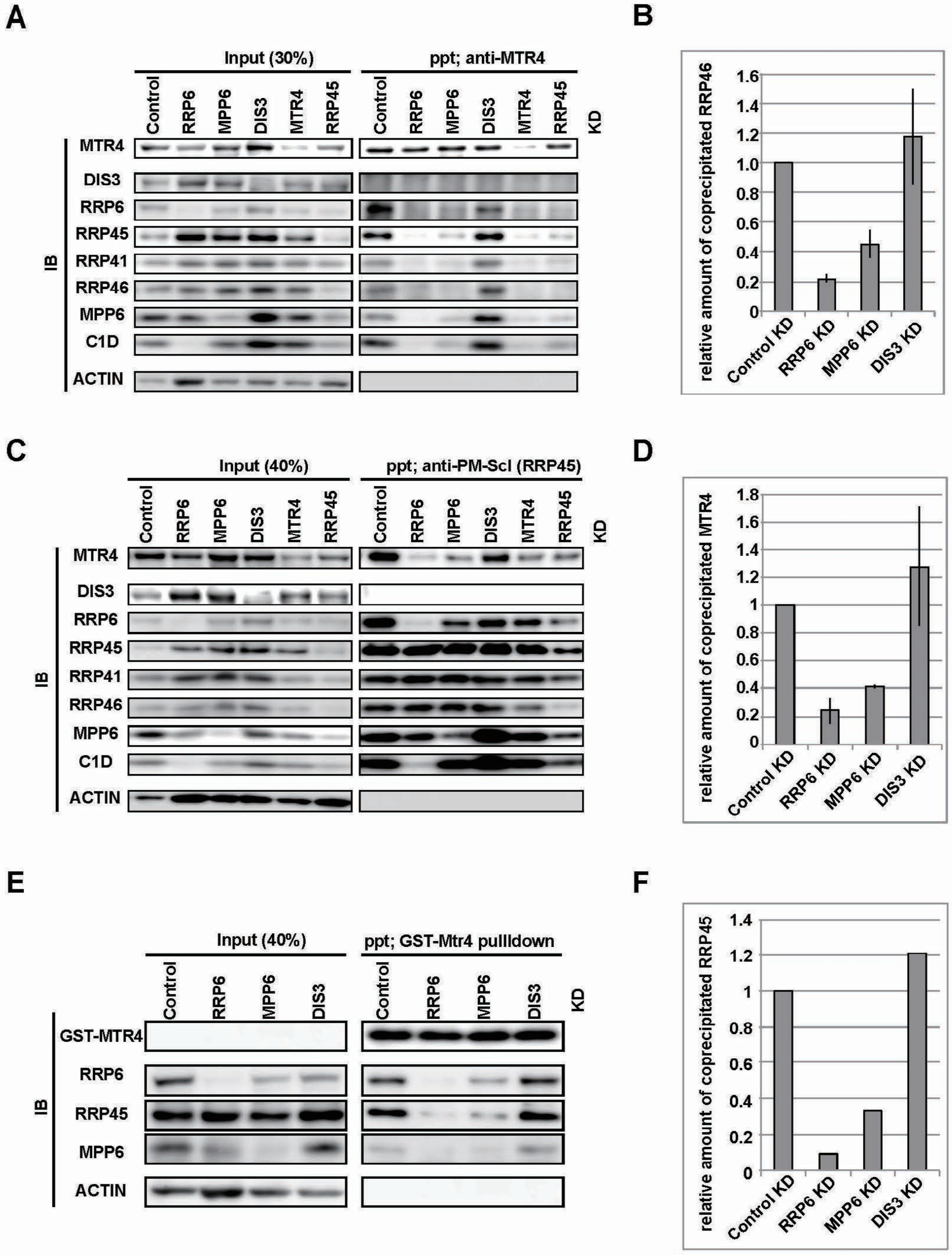
A
Input (30%)
ppt; anti-MTR4
Control
RRP6
MPP6
DIS3
MTR4
RRP45
KD
IB
MTR4
DIS3
RRP6
RRP45
RRP41
RRP46
MPP6
C1D
ACTIN
B
relative amount of coprecipitated RRP46
0
0.2
0.4
0.6
0.8
1.0
1.2
1.4
1.6
Control KD
RRP6 KD
MPP6 KD
DIS3 KD
C
Input (40%)
ppt; anti-PM-Scl (RRP45)
D
relative amount of coprecipitated MTR4
0
0.2
0.4
0.6
0.8
1
1.2
1.4
1.6
1.8
E
Input (40%)
ppt; GST-Mtr4 pulldown
GST-MTR4
F
relative amount of coprecipitated RRP45
0
0.2
0.4
0.6
0.8
1
1.2
1.4

Fig S6. Different degrees of contribution of RRP6, MPP6, and DIS3 to the MTR4-core interaction. (A)-(D) Immunoprecipitations (IPs) to evaluate the effect of depleting either RRP6, MPP6 or DIS3 on the MTR4-core interaction. (E), (F) Evaluation by pulldown assay. (A), (C) IPs were performed on nuclear extracts deprived of factors noted above the panels using anti-MTR4 antiserum in (A) and anti-PM-Scl antiserum in (C). (B), (D) Quantification of two independent anti-MTR4 IP experiments and anti-PM-Scl IP experiments including (A) and (C), respectively. The ratio of either RRP46 to MTR4 in (B) or of MTR4 to RRP45 in (D) in each precipitate was normalized by the respective ratio in each input extract and by the value of the Control KD sample to obtain the relative coprecipitated amount of the protein in each sample. Bars and error bars denote mean values ± *SD*. (E) GST-MTR4 pulldown assay to evaluate the contribution of either RRP6, MPP6 or DIS3 in supporting MTR4-core binding. Recombinantly expressed and purified GST-MTR4 was attached to glutathione Sepharose beads to be mixed with nuclear extracts from HeLa cells depleted of either RRP6, MPP6 or DIS3. (F) Quantification of (E). Values shown are relative amounts of precipitated RRP45 normalized by the amounts of GST-MTR4 in the precipitates and by the value of Control KD sample.
