# Supplementary material for: MPP6 stimulates both RRP6 and DIS3 to degrade a specified subset of MTR4-sensitive substrates in the human nucleus
Source: Nucleic Acids Res. 2022 Jul 29;50(15):8779–806. doi: 10.1093/nar/gkac559 (PMC9410898; doi:10.1093/nar/gkac559)
Supplement: gkac559_Supplemental_Files [file gkac559_supplemental_files.zip › Figure S7.pdf]

Figure S7

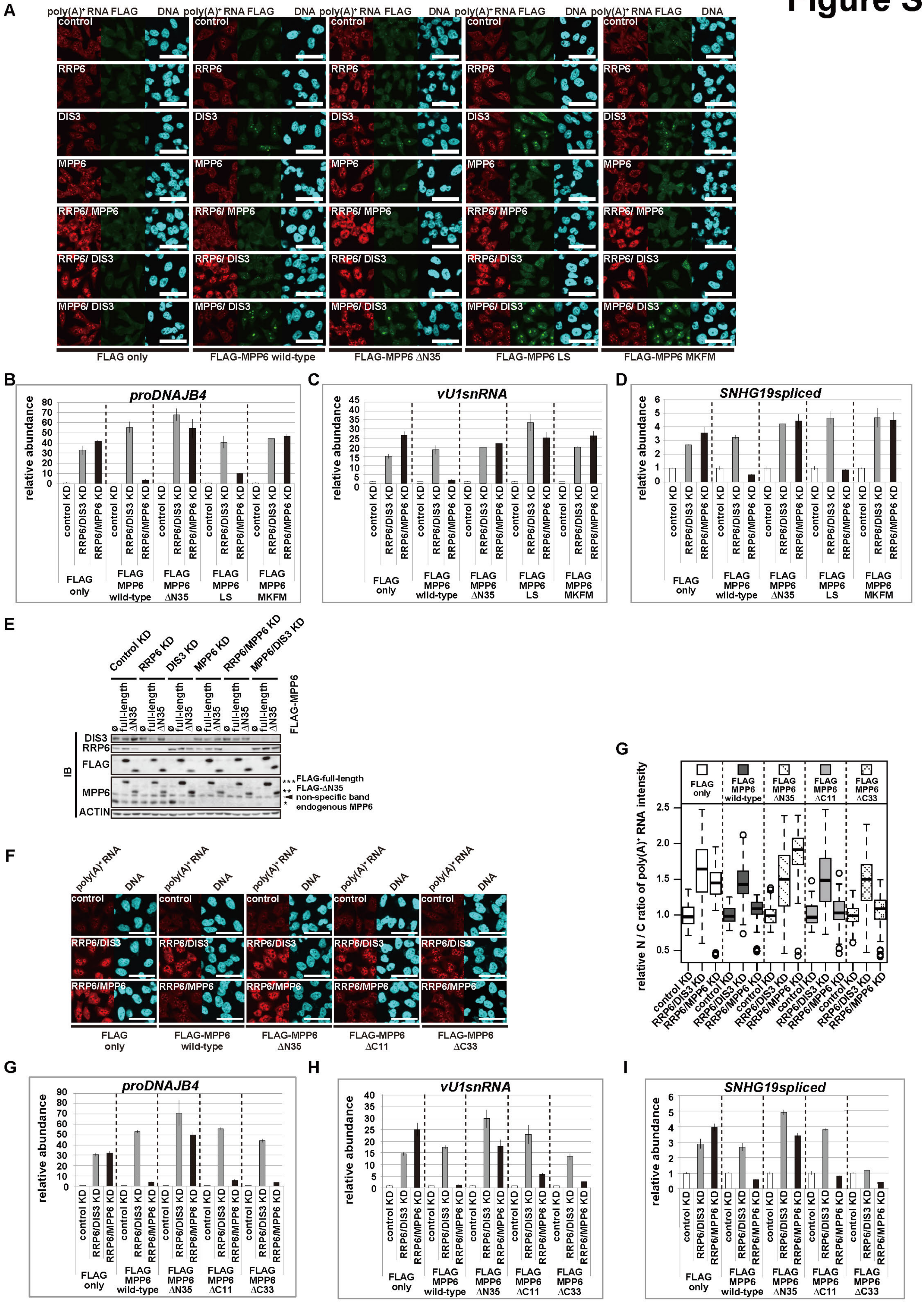
A
poly(A)+ RNA FLAG DNA
control
RRP6
DIS3
MPP6
RRP6/ MPP6
RRP6/ DIS3
MPP6/ DIS3
FLAG only
FLAG-MPP6 wild-type
FLAG-MPP6 ΔN35
FLAG-MPP6 LS
FLAG-MPP6 MKFM
B
proDNAJB4
C
vU1snRNA
D
SNHG19spliced
relative abundance
control KD
RRP6/DIS3 KD
RRP6/MPP6 KD
FLAG only
FLAG MPP6 wild-type
FLAG MPP6 ΔN35
FLAG MPP6 LS
FLAG MPP6 MKFM
E
Control KD
RRP6 KD
DIS3 KD
MPP6 KD
RRP6/MPP6 KD
MPP6/DIS3 KD
ø
full-length
ΔN35
FLAG-MPP6
IB
DIS3
RRP6
FLAG
MPP6
ACTIN
*** FLAG-full-length
** FLAG-ΔN35
non-specific band
* endogenous MPP6
F
poly(A)+ RNA
DNA
control
RRP6/DIS3
RRP6/MPP6
FLAG only
FLAG-MPP6 wild-type
FLAG-MPP6 ΔN35
FLAG-MPP6 ΔC11
FLAG-MPP6 ΔC33
G
relative N / C ratio of poly(A)+ RNA intensity
FLAG only
FLAG MPP6 wild-type
FLAG MPP6 ΔN35
FLAG MPP6 ΔC11
FLAG MPP6 ΔC33
G
proDNAJB4
H
vU1snRNA
I
SNHG19spliced

Figure S7. Functional importance of conserved N-terminal residues of MPP6 in degrading poly(A)$^+$ substrates by the exosome. (A)-(D) Defects in the poly(A)$^+$ substrate decay exhibited by N-terminal MPP6 mutants. (E) The expression of each FLAG-MPP6 mutant is maintained under the conditions of RRP6 depletion. (F)-(I) The negligible effect of C-terminal deleting mutations on MPP6 function in the poly(A)$^+$ substrate decay. (A), (F) Poly(A)$^+$ FISH analysis of HeLa Flp-In T-REx cell lines expressing FLAG-MPP6 mutants. Cell lines tested are stated at the bottom of the panels and depleted factors in the panels. In (A), expressed proteins were visualized by FLAG-staining. Scale bar = 50μm. (B)-(D), (G)-(I) RT-qPCRs to evaluate the function of MPP6 mutants in degrading individual poly(A)$^+$ substrates, namely (B),(G) *proDNAJB4*, (C),(H) *vU1snRNA* and (D),(I) spliced *SNHG19*. PCR was performed on dT_{25}-primed cDNA synthesized using total RNA from whole cells. Values shown are relative amounts of each transcript normalized by *GAPDH* and by the value of Control KD sample within each cell line. (E) Immunoblotting was performed to nuclear extracts from HeLa Flp-In T-REx cell lines expressing FLAG-MPP6 mutants. Extracts were prepared under various KD conditions as noted above the panels. (G) Quantification of (F). Relative N/C ratio of poly(A)$^+$ FISH signal normalized to the mean value of Control KD cells within each cell line. *n* = 100.
