# Supplementary material for: MPP6 stimulates both RRP6 and DIS3 to degrade a specified subset of MTR4-sensitive substrates in the human nucleus
Source: Nucleic Acids Res. 2022 Jul 29;50(15):8779–806. doi: 10.1093/nar/gkac559 (PMC9410898; doi:10.1093/nar/gkac559)
Supplement: gkac559_Supplemental_Files [file gkac559_supplemental_files.zip › Figure S8.pdf]

Figure S8

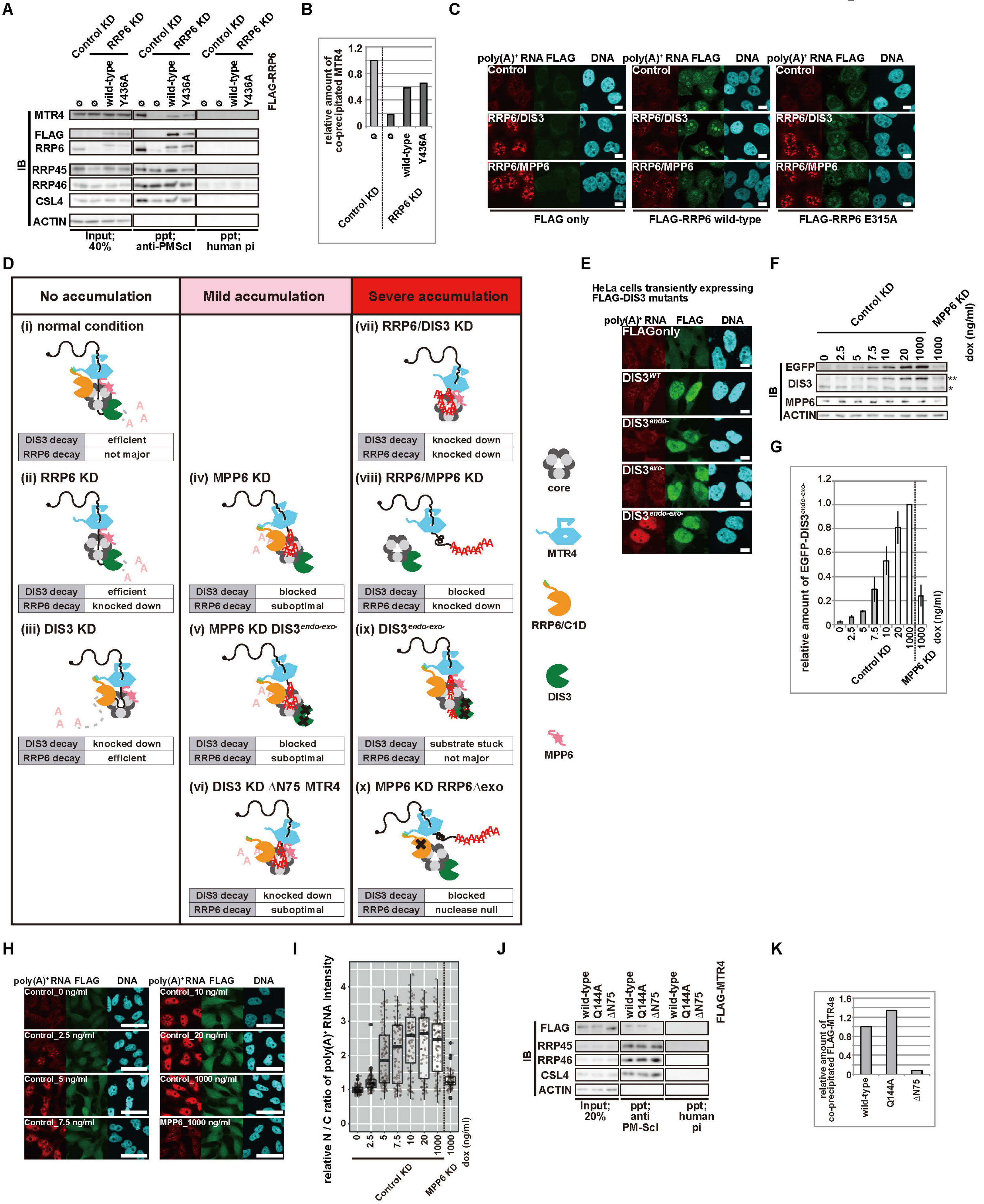
A
Control KD
RRP6 KD
wild-type
Y436A
FLAG-RRP6
MTR4
FLAG
RRP6
RRP45
RRP46
CSL4
ACTIN
IB
Input; 40%
ppt; anti-PMScl
ppt; human pi
B
relative amount of co-precipitated MTR4
Control KD
RRP6 KD
C
poly(A)⁺ RNA FLAG DNA
Control
RRP6/DIS3
RRP6/MPP6
FLAG only
FLAG-RRP6 wild-type
FLAG-RRP6 E315A
D
No accumulation
Mild accumulation
Severe accumulation
(i) normal condition
DIS3 decay efficient
RRP6 decay not major
(ii) RRP6 KD
DIS3 decay efficient
RRP6 decay knocked down
(iii) DIS3 KD
DIS3 decay knocked down
RRP6 decay efficient
(iv) MPP6 KD
DIS3 decay blocked
RRP6 decay suboptimal
(v) MPP6 KD DIS3endo-exo-
DIS3 decay blocked
RRP6 decay suboptimal
(vi) DIS3 KD ΔN75 MTR4
DIS3 decay knocked down
RRP6 decay suboptimal
(vii) RRP6/DIS3 KD
DIS3 decay knocked down
RRP6 decay knocked down
(viii) RRP6/MPP6 KD
DIS3 decay blocked
RRP6 decay knocked down
(ix) DIS3endo-exo-
DIS3 decay substrate stuck
RRP6 decay not major
(x) MPP6 KD RRP6Δexo
DIS3 decay blocked
RRP6 decay nuclease null
core
MTR4
RRP6/C1D
DIS3
MPP6
E
HeLa cells transiently expressing FLAG-DIS3 mutants
poly(A)⁺ RNA FLAG DNA
FLAGonly
DIS3WT
DIS3endo-
DIS3exo-
DIS3endo-exo-
F
Control KD
MPP6 KD
0 2.5 5 7.5 10 20 1000 1000
dox (ng/ml)
EGFP
DIS3
MPP6
ACTIN
IB
G
relative amount of EGFP-DIS3endo-exo-
dox (ng/ml)
Control KD
MPP6 KD
H
poly(A)⁺ RNA FLAG DNA
Control_0 ng/ml
Control_2.5 ng/ml
Control_5 ng/ml
Control_7.5 ng/ml
Control_10 ng/ml
Control_20 ng/ml
Control_1000 ng/ml
MPP6_1000 ng/ml
I
relative N / C ratio of poly(A)⁺ RNA Intensity
dox (ng/ml)
Control KD
MPP6 KD
J
wild-type
Q144A
ΔN75
FLAG-MTR4
FLAG
RRP45
RRP46
CSL4
ACTIN
IB
Input; 20%
ppt; anti PM-Scl
ppt; human pi
K
relative amount of co-precipitated FLAG-MTR4s
wild-type
Q144A
ΔN75

Figure S8. MTR4 recruited by RRP6 fails to efficiently support poly(A)$^+$ substrate decay mediated by DIS3 in the absence of MPP6. (A), (B) Y436A, a catalytically inert RRP6 mutant, is efficiently incorporated into the exosome complex. (C) E315A, another catalytically inert RRP6 mutant, failed to restore nuclear poly(A)$^+$ RNA accumulation derived from RRP6/MPP6 KD. (D) Graphical illustrations depicting the status of substrate decay by the exosome under each condition examined in our analysis. DIS3 mainly degrades poly(A)$^+$ substrates under normal conditions (i). Efficient substrate decay is achieved even when RRP6 is depleted (ii). Upon DIS3 KD, RRP6 compensates for the function of DIS3 (iii). In the absence of MPP6, substrates do not reach DIS3 efficiently (iv, v, x), hence MPP6 KD attenuates the poly(A)$^+$ accumulating phenotype induced by the expression of DIS3$^{endo\text{-}exo\text{-}}$ (v, ix). For optimal RRP6 activity, MTR4 binding to both RRP6 and MPP6 is essential (iv, v, vi). (E) Transient expression of the FLAG-DIS3 mutants and their effects on the accumulation of bulk poly(A)$^+$ substrates in the HeLa nucleoplasm. (F)-(I) Manipulation of EGFP-DIS3$^{endo\text{-}exo\text{-}}$ expression by adjusting the concentration of doxycycline (dox) in the media. (J), (K) RRP6-MTR4 interaction is indispensable for optimal poly(A)$^+$ substrate decay mediated by RRP6. (A), (J) Immunoprecipitation (IP) experiments using anti-PM-Scl antiserum. Human pre-immune (pi) serum was used as a control in the IP step. Cell lines and knockdown conditions are indicated at the top of the panels. Ø denotes FLAG only. (B), (K) Quantification of (A) and (J), respectively. Ratio of either MTR4 to RRP45 in (B) or FLAG-MTR4 to RRP45 in (K) in each precipitate was normalized by the corresponding ratio in each input extract. Values obtained were further normalized by the value of Control KD FLAG only expressing sample in (B) and by the value of FLAG-MTR4 wild-type expressing samples in (K). (C), (E), (H) Poly(A)$^+$ FISH analyses combined with FLAG-staining. Conditions of cell lines, expressed proteins, depleted factors and dox concentrations are stated at the bottom and in the panels. Scale bar = 10 μm in (C) and (E), 50μm in (H). (F) Immunoblot analysis to evaluate the amount of induced EGFP-DIS3$^{endo\text{-}exo\text{-}}$. Conditions are denoted at the top. (G) Quantification of (F). Values shown are relative abundances of EGFP-DIS3$^{endo\text{-}exo\text{-}}$ normalized by the ACTIN amount and the value of the 1000 ng/ml dox supplemented Control KD sample. Bars represent mean values of two technical replicates and error bars ± *SEM*. (I) Quantification of (H). Relative N/C ratio of poly(A)$^+$ FISH signal normalized to the mean value of the 0 ng/ml dox supplemented Control KD cells. The conditions that the cells were subjected to are shown at the bottom. n = 100.
