# Supplementary material for: MPP6 stimulates both RRP6 and DIS3 to degrade a specified subset of MTR4-sensitive substrates in the human nucleus
Source: Nucleic Acids Res. 2022 Jul 29;50(15):8779–806. doi: 10.1093/nar/gkac559 (PMC9410898; doi:10.1093/nar/gkac559)
Supplement: gkac559_Supplemental_Files [file gkac559_supplemental_files.zip › Figure S9.pdf]

Figure S9

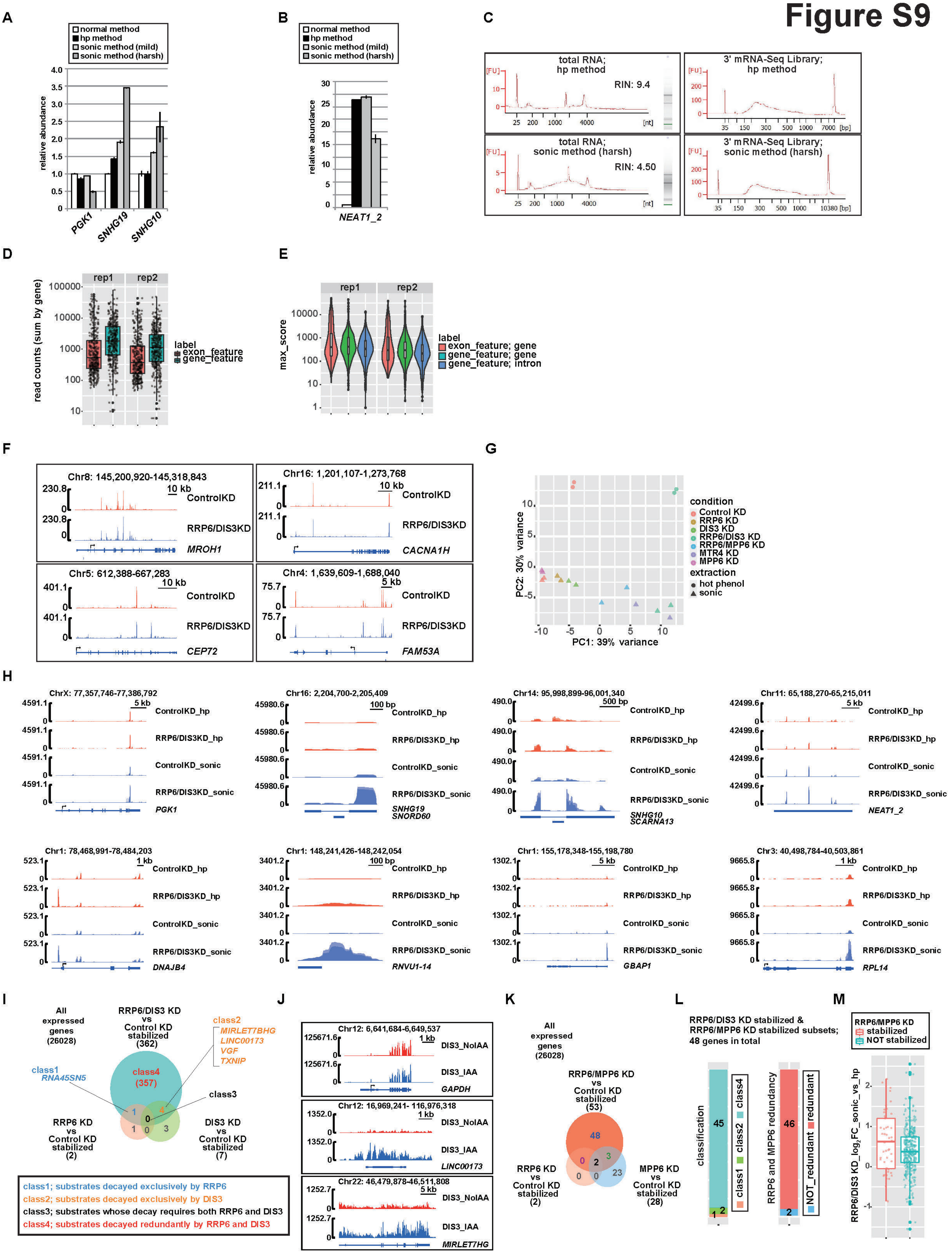
A
normal method
hp method
sonic method (mild)
sonic method (harsh)
relative abundance
PGK1
SNHG19
SNHG10
B
NEAT1_2
C
total RNA; hp method
RIN: 9.4
3' mRNA-Seq Library; hp method
total RNA; sonic method (harsh)
RIN: 4.50
3' mRNA-Seq Library; sonic method (harsh)
D
rep1
rep2
read counts (sum by gene)
label
exon_feature
gene_feature
E
max_score
exon_feature; gene
gene_feature; gene
gene_feature; intron
F
Chr8: 145,200,920-145,318,843
ControlKD
RRP6/DIS3KD
MROH1
Chr16: 1,201,107-1,273,768
CACNA1H
Chr5: 612,388-667,283
CEP72
Chr4: 1,639,609-1,688,040
FAM53A
G
PC1: 39% variance
PC2: 30% variance
condition
Control KD
RRP6 KD
DIS3 KD
RRP6/DIS3 KD
RRP6/MPP6 KD
MTR4 KD
MPP6 KD
extraction
hot phenol
sonic
H
ChrX: 77,357,746-77,386,792
ControlKD_hp
RRP6/DIS3KD_hp
ControlKD_sonic
RRP6/DIS3KD_sonic
PGK1
Chr16: 2,204,700-2,205,409
SNHG19
SNORD60
Chr14: 95,998,899-96,001,340
SNHG10
SCARNA13
Chr11: 65,188,270-65,215,011
NEAT1_2
Chr1: 78,468,991-78,484,203
DNAJB4
Chr1: 148,241,426-148,242,054
RNVU1-14
Chr1: 155,178,348-155,198,780
GBAP1
Chr3: 40,498,784-40,503,861
RPL14
I
All expressed genes (26028)
RRP6/DIS3 KD vs Control KD stabilized (362)
class2
MIRLET7BHG
LINC00173
VGF
TXNIP
class1
RNA45SN5
class4 (357)
class3
RRP6 KD vs Control KD stabilized (2)
DIS3 KD vs Control KD stabilized (7)
class1; substrates decayed exclusively by RRP6
class2; substrates decayed exclusively by DIS3
class3; substrates whose decay requires both RRP6 and DIS3
class4; substrates decayed redundantly by RRP6 and DIS3
J
Chr12: 6,641,684-6,649,537
DIS3_NoIAA
DIS3_IAA
GAPDH
Chr12: 16,969,241- 116,976,318
LINC00173
Chr22: 46,479,878-46,511,808
MIRLET7HG
K
All expressed genes (26028)
RRP6/MPP6 KD vs Control KD stabilized (53)
RRP6 KD vs Control KD stabilized (2)
MPP6 KD vs Control KD stabilized (28)
L
RRP6/DIS3 KD stabilized & RRP6/MPP6 KD stabilized subsets; 48 genes in total
classification
class4
class2
class1
RRP6 and MPP6 redundancy
redundant
NOT_redundant
M
RRP6/MPP6 KD
stabilized
NOT stabilized
RRP6/DIS3 KD_log2FC_sonic_vs_hp

Figure S9. Characterization of the exosome substrates and the MPP6-susceptible subclass. (A)-(C) The effect of sonication during the RNA extraction step. (D)-(F) Multiple false positives were detected in the analysis including the read counts of long introns covered with low-density reads. (G)-(M) NGS analyses and their supporting data. (A), (B) RT-qPCRs to quantify (A) *PGK1*, *SNHG19* and *SNHG10*, and (B) *NEAT1_2* extracted from RRP6/DIS3 KD HeLa nuclei. RNA extraction was performed by either the normal, the hot-phenol (hp) employing or the sonic employing method. The harsh sonic-method employed 20 repetitions of 1 sec of sonication followed by 1 sec of holding while the mild sonic method involved 10 repetitions. Extraction steps and RT-qPCRs were performed on two technical replicates. The values are relative abundances of each transcript normalized by *GAPDH* and by the value of the samples prepared by the normal extracting method. Bars and error bars denote mean values ± *SEM*. (C) Electropherograms to represent the size distribution of the total RNAs extracted from HeLa cells (left panels) and of their resulting 3' mRNA-seq cDNA libraries (right panels). The upper row shows data from samples of the hp-method and the lower row from sonic-method sample. The vertical axis indicates fluorescence, and the horizontal axis indicates the molecular size. (D) Read counts by gene were compared between the exosome substrates identified from the exon-feature counting (362 substrates) and those identified from the gene-feature counting (373 substrates). Reads uniquely mapped in RRP6/DIS3 KD samples were counted. Counts were compared within each replicate, rep1 and rep2. (E) Although the total number of reads from gene-feature counting overwhelms the results from exon counting, as shown in (D), the maximum read coverage scores in gene bodies and introns for the substrates identified by gene-feature counting remains small. Maximum read coverage scores for genes extracted with either exon-feature counting or gene-feature counting to RRP6/DIS3 KD samples were violin plotted. The max scores by gene confined to introns are also shown for gene-feature counting. (F) Genome browser views of multiple false-positive suspicions identified by gene-feature counting. The location in the genome is noted at the top of the tracks, with the gene name and structure at the bottom. Read counts are shown at the left of the tracks. The overlays of the two replicates are shown. Control KD samples are shown in red and RRP6/DIS3 KD samples in blue. (G) Principal component analysis (PCA) of our NGD samples. Using DESeq2, read counts were normalized with the ERCC spike-ins as controls between samples to obtain regularized log-transformed read counts (reference 119 in the main text). These normalized counts were then subjected to PCA. Conditions under which the samples were prepared are color-coded as described on the right. Extraction methods are labeled by the circles and by the triangles. (H)

Genome browser views within the regions of genes are noted in black letters in Figure 8F and of *PGK1* as a control. Data are described in the same way as in (F). Samples from the hp-method are described in red and those from the sonic-method in blue. (I) Venn diagram to extract the substrates decayed redundantly by RRP6 and DIS3 (Class4 corresponds to such substrates). Descriptions of the substrates in other classes are mentioned in the inset as well as in the main text. (J) Stabilization of *LINC00173* and *MIRLET7HG* upon rapid depletion of DIS3. *GAPDH* was used as a control. Genome browser views within these genes from the published data (reference 36 in main text) are described in the same way as in (F). DIS3_NoIAA denotes Control samples and DIS3_IAA indicates DIS3 depleted samples. (K) Venn diagram to extract the substrates decayed redundantly by RRP6 and MPP6. (L) Most of the RRP6/MPP6 KD stabilized substrates are redundantly degraded by RRP6 and DIS3, as well as by RRP6 and MPP6. RRP6/DIS3 KD stabilized substrates that were also stabilized by RRP6/MPP6 KD were sorted by substrate class determined in (I) (left panel, named as classification) as well as by the functional redundancy between RRP6 and MPP6 on them obtained from (K) (right panel, named as RRP6 and MPP6 redundancy). Bar charts are color-coded as described in the inset on the right of each panel. (M) The effect of sonication on the extraction efficiency of the transcripts from RRP6/DIS3 KD-sensitive substrates in either subclass of RRP6/MPP stabilized or of RRP6/MPP NOT stabilized. The $\log_2$ fold change of each substrate in the sonic samples against the hp samples was plotted.
